# Targeting macrophage circadian rhythms with microcurrent stimulation to activate cancer immunity through phagocytic defense

**DOI:** 10.7150/thno.100748

**Published:** 2025-01-01

**Authors:** Yuya Yoshida, Tomohito Tanihara, Keika Hamasaki, Fumiaki Tsurusaki, Taiki Fukuda, Satoka Adachi, Yuma Terada, Kaita Otsuki, Naoki Nishikawa, Kohei Fukuoka, Ryotaro Tsukamoto, Kengo Hamamura, Kosuke Oyama, Akito Tsuruta, Kouta Mayanagi, Satoru Koyanagi, Shigehiro Ohdo, Naoya Matsunaga

**Affiliations:** 1 Department of Clinical Pharmacokinetics, Faculty of Pharmaceutical Sciences, Kyushu University, 3-1-1 Maidashi Higashi-ku, Fukuoka 812-8582, Japan.; 2 Department of Pharmaceutics, Faculty of Pharmaceutical Sciences, Kyushu University, 3-1-1 Maidashi Higashi-ku, Fukuoka 812-8582, Japan.; 3 Department of Biological Science and Technology, Faculty of Advanced Engineering, Tokyo University of Science, 1-3 Kagurazaka, Shinjuku-ku, Tokyo 162-8601, Japan.; 4 Department of Drug Discovery Structural Biology, Faculty of Pharmaceutical Sciences, Kyushu University, 3-1-1 Maidashi Higashi-ku, Fukuoka 812-8582, Japan.

**Keywords:** cancer immunotherapy, macrophage, phagocytosis, circadian rhythm, microcurrent stimulation

## Abstract

**Rationale:** Macrophage phagocytosis plays a role in cancer immunotherapy. The phagocytic activity of macrophages, regulated by circadian clock genes, shows time-dependent variation. Intervening in the circadian clock machinery of macrophages is a potentially novel approach to cancer immunotherapy; however, data on this approach are scarce. Microcurrent stimulation (MCS) promotes inflammation, proliferation, and remodeling, suggesting its potential to modulate macrophage function; however, its application has been limited. In this study, we investigated the impact of MCS on macrophage phagocytosis of cancer cells using mouse/human macrophage cell lines and various mouse/human cancer cell lines.

**Methods:** Cells and mice received 300 µA, 400 Hz bidirectional pulsed MCS. Gene expression, protein expression, and phagocytosis activity were assessed in intraperitoneal macrophages collected from mice, as well as in RAW264.7, and THP-1 cells. Flow cytometry, population, phagocytosis activity, RNA-seq, and immunohistochemistry analyses were performed.

**Results:** Noninvasive MCS prevented time-dependent reduction in macrophage phagocytosis of cancer cells by modulating the circadian clock genes. MCS also enhanced phagocytosis in mouse RAW264.7 and human THP-1 cells across various cancer types by promoting actin polymerization; similar *in vivo* effects were observed in mice. This enhancement occurred in abdominal macrophages of both sexes and was mediated by changes in clock gene expression. Specifically, suppressing the clock gene *Per1* nullified the effects of MCS. Moreover, although macrophage phagocytosis typically declined during the dark period, MCS during the light period prevented this reduction. MCS also increased phagocytosis of peritoneally implanted cancer cells (4T1, ID8, and Hepa1-6) in mice, significantly reducing tumor engraftment and growth, and ultimately improving prognosis.

**Conclusions:** The findings of this study suggest that targeting macrophage circadian mechanisms via MCS could enhance cancer immunity, offering new avenues for cancer immunotherapy.

## Introduction

Cancer immunotherapy leverages the body's immune function to eradicate cancer cells and has become a pivotal treatment modality for malignant tumors. T-cell immune checkpoint inhibitors (ICIs), such as nivolumab, target tumor-T cell interactions mediated by PD-1 and PD-L1, offering therapeutic avenues for various malignancies, such as melanoma, lung cancer, and breast cancer 1-3. Beyond T cell checkpoints, the macrophage immune checkpoint, known as the "don't eat me" signal, constitutes a significant mechanism in cancer immunity 4. Macrophages eliminate cancer cells via phagocytosis while stimulating tumor growth inhibition through immune cell activation, including that of B, memory T, and killer T cells, facilitated by antigen presentation and cytokine production 5. The "don't eat me" signal, orchestrated by CD47 and SIRPα, inhibits macrophage phagocytosis, with anti-CD47 antibodies enhancing macrophage-mediated tumor shrinkage 6. The notable response rate for magrolimab in patients with acute myeloid leukemia underscores the potential of targeting macrophage phagocytosis as a novel cancer immunotherapy 7.

The development of T cell immune checkpoint therapy has seen slower progress in breast cancer, characterized by a tumor mutation burden lower than that of melanoma or lung cancer 8, with response rates in patients treated solely with T cell ICIs falling below 30% 9,10. Conversely, the increased expression of CD47 in breast cancer suggests the potential efficacy of therapies targeting the macrophage immune checkpoint or macrophage phagocytosis in this context 11.

Macrophage phagocytosis is modulated by SIRPα expressed on the plasma membrane and activated by various receptors, such as MerTK, CD36, and LRP 12. Extracellular cytokine stimulation alters the expression of these receptors, shaping macrophage subtypes. Macrophages with phagocytic activity encapsulate foreign substances by reorganizing their actin skeleton into phagosomes. Furthermore, macrophage phagocytosis is subject to regulation by the circadian clock machinery, which exhibits diurnal fluctuations 13-15. This conserved mechanism, present across most organisms, aligns physiological and behavioral functions with daily environmental changes 16. In mammals, this mechanism is regulated by a molecular oscillator governed by a transcription-translation feedback loop involving clock genes 17,18. The products of *clock* and *Arntl* (also known as *Bmal1*) form heterodimers that promote the transcription of period (Per) and cryptochrome (Cry) genes; when PER and CRY proteins reach critical levels, transcriptional activation via CLOCK/ARNTL is repressed. These clock genes modulate the circadian rhythms of various functional molecules, potentially exacerbating diverse diseases 19,20. Given the involvement of the macrophage circadian clock in disease progression, the expression of clock genes in macrophages correlates with various conditions 21,22, such as chronic kidney disease 23 and multiple sclerosis 24. Moreover, the macrophage circadian clock may influence the efficacy of ICIs. Diurnal variations in macrophage phagocytosis 25,26, along with the fact that the response rate of nivolumab depends on the time of administration and aligning ICI administration with the macrophage circadian clock enhances efficacy in tumor-bearing mice 27,28, underscore the role of the macrophage circadian clock in cancer immunotherapy resistance. Intervening in the circadian clock machinery of macrophages could thus represent a novel approach to cancer immunotherapy; however, only a few studies have explored this.

Efforts to modulate the circadian clock mechanism have been ongoing, yielding several agonists/antagonists for clock genes 29,30. However, the cyclic nature of the clock mechanism poses challenges for drug-based interventions, as existing methods typically exert permanent effects until metabolism. We have previously reported that the expression of the clock gene *Period1* (*Per1*) can be transiently altered with 15 min of microcurrent stimulation (MCS), an electrical stimulus within physiological limits (~500 μA), akin to endogenous neurotransmission 31. Widely used in wound healing, MCS promotes inflammation, proliferation, and remodeling 32-35, suggesting its potential to modulate macrophage function; however, only a few studies have explored this 32,36. Moreover, except for our previous study 31 and research on bacteria 37, the current literature on the interplay in the context of cancer immunity, MCS, and the circadian clock mechanism remains scant.

Therefore, in this study, we investigated the impact of MCS on macrophage phagocytosis against cancer cells using mouse/human macrophage cell lines and various mouse/human cancer cell lines. We explore the previously unexplored effects of current stimulation to highlight the efficacy of intervening with the macrophage circadian clock mechanism in cancer therapy. We evaluate the outcomes in a mouse model of carcinoma to further elucidate the relationship between MCS effects on macrophages and the circadian clock mechanism.

## Results

### MCS augments cancer cell phagocytosis by mouse/human macrophage cell lines

**Figure 1:**
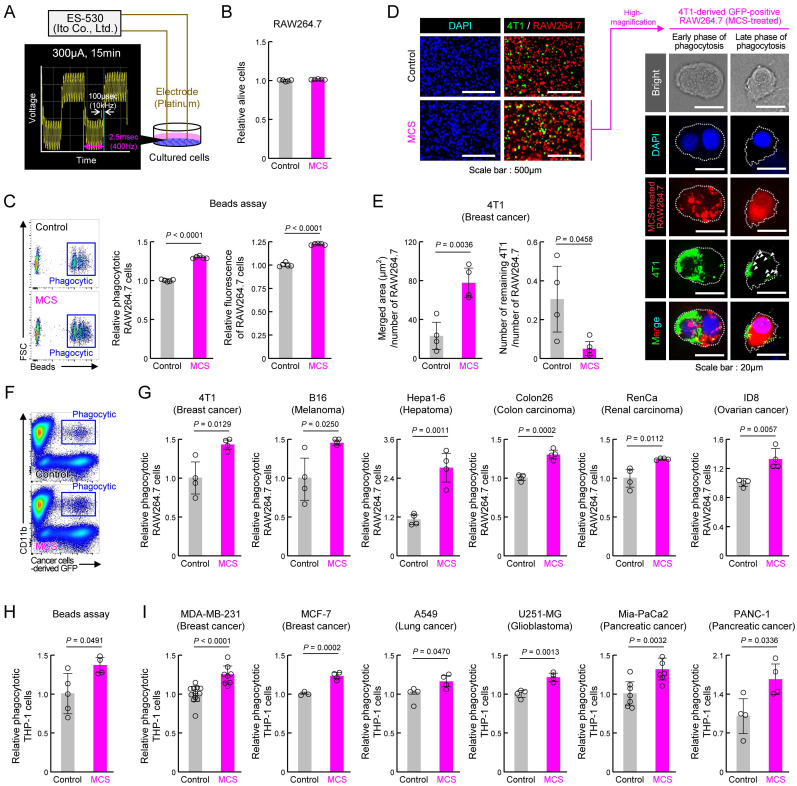
** Effect of MCS on cancer cell phagocytosis by RAW264.7 and THP-1 macrophages.** (**A**) Schematic illustration of the MCS treatment protocol for RAW264.7 and THP-1 cells. Phagocytosis was assessed 12 h post-MCS treatment. (**B**) Comparison of survival rates of RAW264.7 cells following 12 h of MCS treatment. (**C**) Effect of MCS treatment on phagocytic activity of RAW264.7 cells on opsonized beads. Left panels depict flow cytometric analysis of the phagocytosis assay. The middle panel illustrates the difference in the ratio of opsonized bead-derived FITC^+^ macrophage cell populations between non-MCS- and MCS-treated cells. The right panel shows the variance in phagocytosed beads per cell. Beads were added 12 h after MCS and co-incubated for another 3 h. The phagocytic activity was measured immediately after the addition of the beads. (**D,E**) Visualization of GFP-positive 4T1 cells (green) and Control or MCS-treated RAW264.7 cells (red) co-cultured for 3 h. For panel D, the representative GFP-positive RAW264.7 cells in the MCS treatment group on the left panel are shown enlarged on the right panel. The white arrow indicates a fragment of 4T1 cells phagocytosed by RAW264.7. For panel E, the comparison of the area of GFP derived from 4T1 that overlaps with RAW264.7 is shown on the left panel, and the comparison of the number of 4T1 cells that do not overlap with RAW264.7 is shown on the right panel. (**F**) Representative flow cytometry panel for assessing the phagocytosis capacity of RAW264.7 cells using GFP-positive 4T1. The gating strategy is outlined in **Figure S19**. (**G**) Relative number of RAW264.7 cells that have phagocytosed 4T1, B16, Hepa1-6, Colon26, RenCa, and ID8 cells. The phagocytic activity was measured immediately after the addition of each cancer cell 12 h after MCS and co-incubated for another 3 h. (**H**) Effect of MCS on phagocytic activity of THP-1 cells on opsonized beads. Cells were differentiated using PMA exposure for 48 h to evaluate phagocytosis. The phagocytic activity was measured immediately after beads were added 12 h after MCS and co-incubated for another 3 h. (**I**) Relative number of PMA-treated THP-1 cells that have phagocytosed MDA-MB-231, MCF-7, A549, U251-MG, Mia-PaCa2, and PANC-1 cells. The phagocytic activity was measured immediately after the addition of each cancer cell 12 h after MCS and co-incubated for another 3 h. Data are expressed as the mean ± S.D. (n = 4-10). The control value is normalized to 1.0. Statistical significance was determined using two-tailed Student's *t*-tests.* P*-values are shown in each graph. FITC: fluorescein isothiocyanate; MCS: microcurrent stimulation; PMA: phorbol 12-myristate 13-acetate; S.D.: standard deviation.

To assess the impact of MCS on macrophage phagocytosis, we first subjected the mouse macrophage cell line RAW264.7 to bidirectional pulsed MCS at 300 μA, 400 Hz for 15 min, following the protocol of a previous study 31 (Figures 1A, S1). Cell viability 12 h post-MCS resembled that of the control group (Figure 1B). Conversely, MCS significantly enhanced RAW264.7 phagocytic activity, as evidenced by increased phagocyte percentage and number of beads phagocytosed per cell, measured by the addition of fluorescent beads opsonized for phagocytosis to the medium (*P* < 0.01 for both, Figure 1C). To determine whether MCS similarly affects cancer cell phagocytosis, MCS-treated RAW264.7 or control cells were co-cultured with the mouse breast cancer cell line 4T1 for 3 h and observed under fluorescent microscopy (Figure 1D). In the magnified image of the binding and overlap between 4T1 (green) and RAW264.7 (red) cells in the MCS treatment group, 4T1 early-fragmentation with nuclei were observed in RAW264.7 cells, suggesting early phase of phagocytosis. Additionally, fragmented 4T1 were also observed in RAW264.7 cells, suggesting late phase of phagocytosis (Figure 1D; right). The area where the fluorescence from 4T1 overlapped with RAW264.7 was significantly higher in the MCS group (*P* < 0.01, Figure 1E; left), and the number of 4T1 cells not engulfed, i.e., that did not overlap with RAW264.7, was significantly lower in the MCS group (*P* < 0.05, Figure 1E; right). These results suggested that MCS led to an increase in the number of RAW264.7 cells that had phagocytosed 4T1 cells while simultaneously reducing the number of surviving 4T1 cells. Furthermore, the GFP-positive 4T1-derived fluorescence in RAW264.7 cells was also detected using flow cytometry (Figure 1F). Co-cultures of MCS-treated RAW264.7 cells with 4T1, mouse melanoma cell line B16, mouse liver cancer cell line Hepa1-6, mouse colon cancer cell line Colon2-6, mouse kidney cancer cell line RenCa, and mouse ovarian surface epithelial cell line ID8, showed significantly higher numbers of GFP-positive CD11b-positive cells, indicating increased cancer cell phagocytosis compared with that of control-treated RAW264.7 cells (*P* < 0.05 for 4T1, B16, and RenCa, *P* < 0.01 for Hepa1-6, Colon26, and ID8, Figure 1G). Notably, MCS did not affect the survival of non-cancer cell-derived mouse embryonic fibroblast (MEF) cell lines and mouse cultured astrocytes (Figure S2).

Furthermore, to evaluate the impact of MCS on human cells, we conducted a similar study using human macrophages derived from the human monocytic cell line THP-1 through PMA treatment 38. The results paralleled those of RAW264.7 cells, with MCS increasing opsonized bead phagocytosis by human macrophages (*P* < 0.05, Figure 1H). Co-cultures of human macrophages with the human breast cancer cell lines MDA-MB-231, MCF-7, human lung cancer cell line A549, human glioblastoma cell line U251-MG, and human pancreatic cancer cell lines Mia-PaCa2 and PANC-1 also elicited a significant increase in phagocytosis upon MCS treatment (*P* < 0.05 for A549 and PANC-1, *P* < 0.01 for MDA-MB-231, MCF-7, U251-MG, and Mia-PaCa2, Figure 1I). These findings suggest that MCS enhances macrophage phagocytic activity against cancer cells in both murine and human systems.

### MCS induces actin polymerization in mouse macrophages upon abdominal stimulation

**Figure 2:**
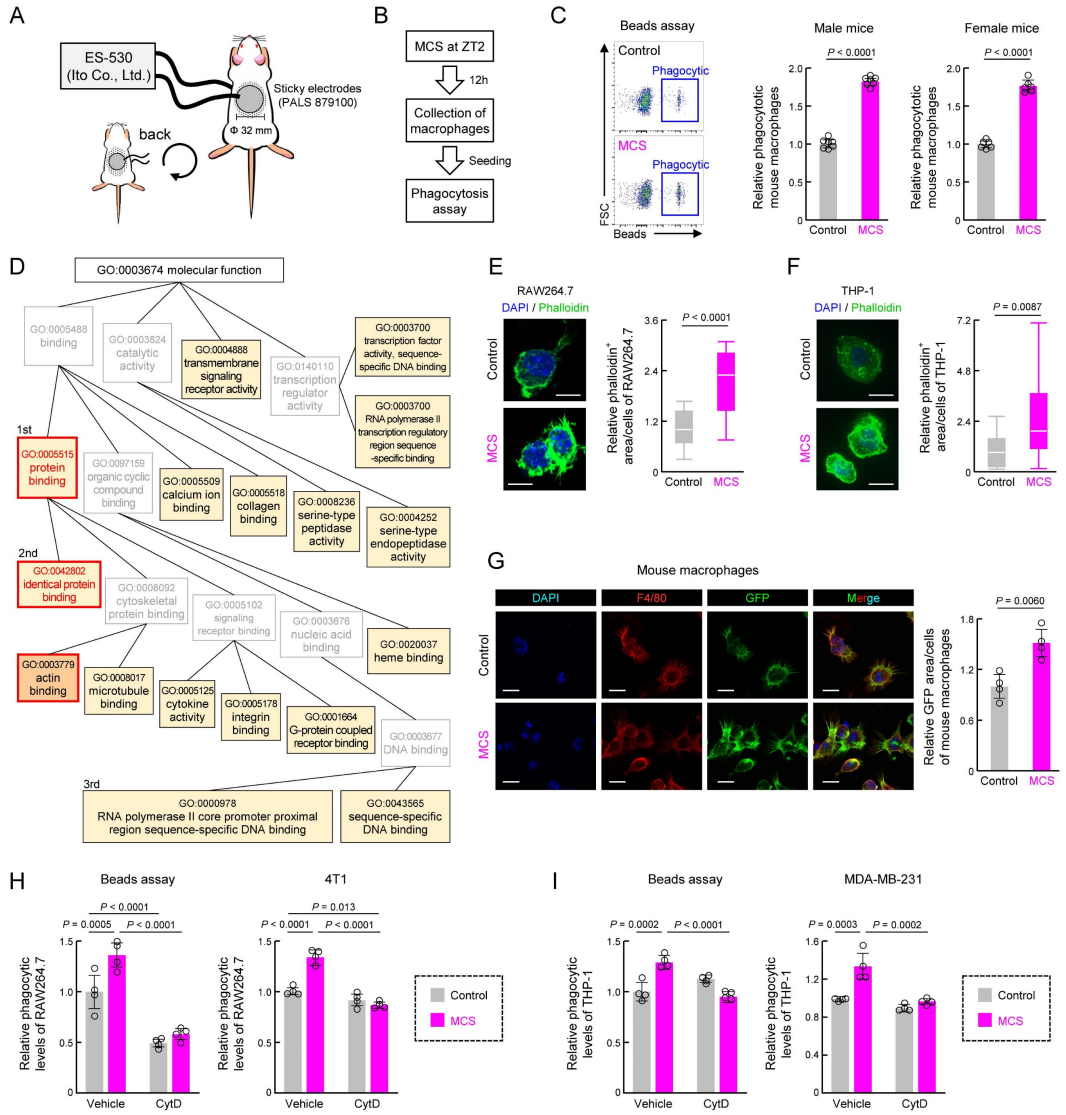
** Effect of abdominal MCS on phagocytic capacity of intraperitoneal macrophages in mice.** (**A**) Schematic illustration detailing the MCS procedure applied to mice. After shaving the abdomen and back, an adhesive pad was utilized for MCS application. (**B,C**) Evaluation of phagocytic activity using opsonized beads. Panel B shows the protocol of MCS treatment. In panel C, the left panels display representative flow cytometry data assessing phagocytosis capacity. The right panels illustrate the difference in the FITC^+^ macrophage cell population ratio between macrophages isolated from control and MCS-treated male and female mice. (**D**) Gene Ontology analysis of genes exhibiting MCS-dependent expression variation, based on RNA-seq results obtained from RNA extracted from intraperitoneal macrophages collected 12 h post-MCS. The analysis includes genes with a Control-to-MCS ratio > 2. From the terms with *P* < 0.05, the top 25 match rates were targeted and arranged based on the hierarchy of parent-child terms. The gene list, all terms with *P* < 0.05 are provided in **Table S1**, **Figure** S**3. (E**). Left: visualization of polymerized actin with GFP-labeled phalloidin in Control or MCS-treated RAW264.7 cells. Scale bar: 30 μm. Right: Calculation of the relative GFP area / DAPI count, indicative of polymerized actin abundance per cell. Twelve hours after MCS, polymerized actin was stained with phalloidin. (**F**) Left: visualization of polymerized actin with GFP-labeled phalloidin in Control or MCS-treated THP-1 cells. Cells were differentiated using PMA exposure for 48 h. Scale bar: 30 μm. Right: Calculation of the relative GFP area / DAPI count, indicative of polymerized actin abundance per cell. Twelve hours after MCS, polymerized actin was stained with phalloidin. (**G**) Visualization of polymerized actin in mouse peritoneal macrophages 12 h post-MCS treatment at ZT2. GFP represents polymerized actin, Red represents F4/80 (macrophage marker), and DAPI represents nuclei. Scale bar: 30 μm. The left panel demonstrates the relative GFP area / F4/80 area, indicating polymerized actin abundance per macrophage. (**H**) Effect of MCS on phagocytosis of beads (left) or 4T1 cells (right) of RAW264.7 under CytD exposure, an inhibitor of actin polymerization. 12 h after MCS, RAW264.7 cells were treated with CytD for 30 m, and then cultured with beads or 4T1 cells for 3 h before measuring phagocytic activity. (**I**) Effect of MCS on phagocytosis of beads (left) or MDA-MB-231 cells (right) of PMA-treated THP-1 under CytD exposure, an inhibitor of actin polymerization. 12 h after MCS, THP-1 cells were treated with CytD for 30 m, and then cultured with beads or MDA-MB-231 cells for 3 h before measuring phagocytic activity. Values represent the mean with standard deviation (F: n = 20, others: n = 4-5). For panels C, E-I, the control group is normalized to 1.0. Statistical significance was determined using two-way ANOVA with Tukey-Kramer post-hoc tests (**H,I**) or two-tailed Student's *t-*tests (**C,E**, **F,G**).* P*-values are shown in each graph. ANOVA: analysis of variance; CytD: cytochalasin D; DAPI: 4′,6-diamidino-2-phenylindole; FITC: fluorescein isothiocyanate; GFP: green fluorescent protein; MCS: microcurrent stimulation; RNA-seq: RNA sequencing; S.D.: standard deviation; ZT: Zeitgeber Time.

To validate the *in vivo* effects of MCS, we applied MCS to the abdomens of mice for 15 min at Zeitgeber Time (ZT) 2, as per our previous study 31, and assessed peritoneal macrophage phagocytosis 12 h later using opsonized beads (Figure 2A,B). The abdominal MCS enhanced phagocytosis by peritoneal macrophages in both male and female mice (*P* < 0.01 for both, Figure 2C).

At the molecular level, bulk RNA-seq followed by GO (https://geneontology.org/) analysis of recovered peritoneal macrophages revealed significant enrichment of several terms following MCS (Figures 2D, S3 and Table S1). First, we focused on protein binding with the highest enrichment rate and its child terms (Figure 2D). Subsequently, we investigated cytokine, M1/M2 differentiation, and SIRPα expression, which are protein interactions strongly correlated with macrophage phagocytic activity. However, MCS did not alter the expression of *Tnfα*, a marker of macrophage inflammatory activity (Figure S4A), percentage of cells expressing CD11c/CD206, expression of markers of M1/M2 macrophages (Figure S4B), or SIRPα expression Figure S4C. Since identical protein binding had the second highest concentration and actin binding, which is also a child term of protein binding, was significantly enriched (Figure 2D), we focused on actin polymerization, which regulates the reorganization of the cytoskeleton necessary for phagocytosis. MCS-induced actin polymerization with pseudopod was evident in RAW264.7, THP-1, and peritoneal macrophages, as observed through phalloidin staining (*P* < 0.01, Figure 2E-G). Additionally, inhibition of actin polymerization 39 using cytochalasin D abolished the MCS-induced increase in phagocytosis of beads and cancer cells (4T1, MDA-MB-231) by RAW264.7 and THP-1 cells (*P* < 0.01, Figure 2H-I). These findings suggest that MCS enhances macrophage phagocytosis by promoting actin polymerization through pathways independent of inflammatory activity or differentiation induction.

### MCS induces alterations in clock gene expression in mouse macrophages upon abdominal stimulation

**Figure 3:**
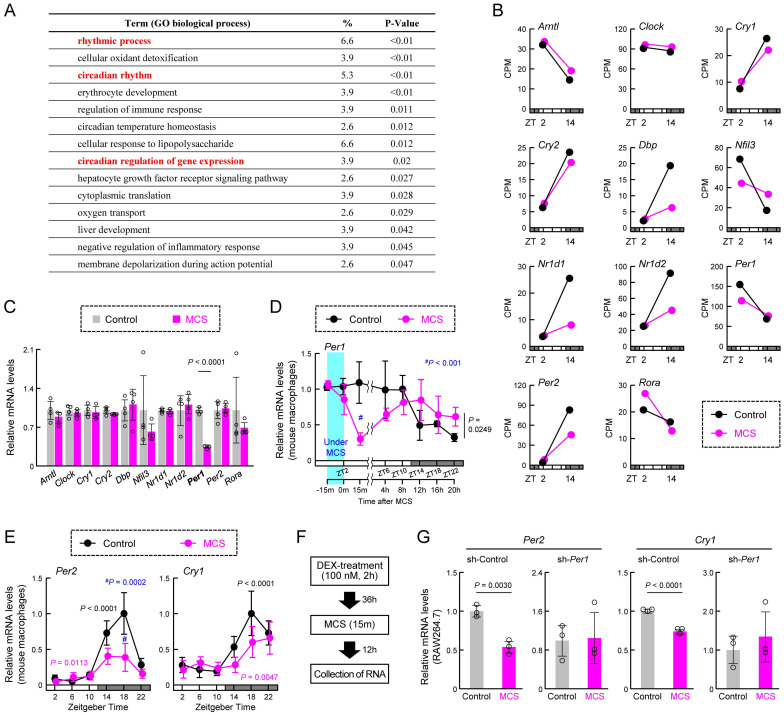
** Effect of MCS on the temporal expression of clock genes in macrophages.** (**A**) Gene Ontology analysis of genes exhibiting MCS-dependent expression variations, based on RNA-seq results obtained from intraperitoneal macrophages collected 12 h post-MCS. The analysis includes genes with a Control-to-MCS ratio > 4. The gene list is provided in **Table S2**. (**B**) Expression levels of key clock genes involved in circadian clock machinery periodicity, including *Arntl*, *Clock*, *Cry1*, *Cry2*, *Dbp*, *Nfil3*, *Nr1d1*, *Nr1d2*, *Per1*, *Per2*, and *Rora*, extracted from RNA-seq results. The values for ZT2 are those in the RNA extracted from macrophages immediately after the end of MCS. The values for ZT14 are those in the RNA extracted from macrophages 12 h after the end of MCS. (**C**) Expression levels of *Arntl*, *Clock*, *Cry1*, *Cry2*, *Dbp*, *Nfil3*, *Nr1d1*, *Nr1d2*, *Per1*, *Per2*, and *Rora* in intraperitoneal macrophages prepared from mice 15 m after MCS. Each mRNA level was measured using RT-qPCR. (**D**) Temporal mRNA expression profiles of *Per1* in intraperitoneal macrophages from Control or MCS-treated female BALB/c mice. The label at the bottom indicates the elapsed time; the time immediately after the end of MCS is defined as 0 m. (**E**) Temporal mRNA expression profiles of *Per2* and *Cry1* in intraperitoneal macrophages from Control or MCS-treated female BALB/c mice. (**F,G**) Effect of MCS on time-dependent decline in the expression of *Per2* and *Cry1* in DEX-treated WT and *Per1*-knockdown RAW264.7 cells. MCS was performed 24 h following treatment with 100 nM DEX for 2 h. The protocol of DEX and MCS treatment is shown in panel F. Data are expressed as the mean ± S.D (n = 4-5). Statistical significance was determined using two-way ANOVA with Tukey-Kramer post-hoc tests.* P*-values are shown in each graph. ANOVA: analysis of variance; MCS: microcurrent stimulation; RNA-seq: RNA sequencing; S.D.: standard deviation.

We next conducted a re-analysis of the RNA-seq data to identify genes affected by MCS, focusing on biological process GO terms. We found significant enrichment of multiple terms related to the circadian clock machinery (Figure 3A). Considering the influence of MCS on transcription-related terms (Figure 2D), it is plausible that MCS affects the expression of clock genes, particularly those encoding transcription factors. Indeed, RNA-seq of macrophages at ZT2, immediately after the end of MCS, and ZT14, 12 h post-MCS, revealed variable expression of multiple clock genes at ZT14 (Figure 3B). Moreover, to identify clock genes with their expression initially altered by MCS, we assessed the expression of clock genes 15 m after the end of MCS. We found that only RT-qPCR of *Per1* mRNA showed a significant change in expression (*P* < 0.01, Figure 3C). *Per1* mRNA expression in macrophages decreased immediately after the start of MCS until 15 m after the end of MCS (Figure 3D, Left); significant differences were observed between control and MCS in the variation at five time points (ZT6, 10, 14, 18, and 22) thereafter (*P* < 0.05, Figure 3D, Right). In addition, *Per2* and *Cry1*, pivotal components of the circadian clock mechanism, at six time points (ZT2, 6, 10, 14, 18, and 22) revealed significant alterations in mRNA expression rhythms (Figure 3E). Therefore, to verify the effect of *Per1* on the changes in *Per2* and *Cry1*, we performed *Per1*-knockdown (Figure S5A) and dexamethasone (DEX) treatment on RAW264.7 cells. DEX treatment induces circadian rhythms in clock gene expression 28,40. In RAW264.7 with sh-Control, the expression of* Per1*, *Per2*, and *Cry1* showed the circadian rhythms (Figure S5B,C). *Per1*-knockdown RAW264.7 cells also showed circadian rhythms in the expression of *Per2* and *Cry1*; however, the expression of these genes 48 h after DEX treatment, which was the peak time, was lower than that of sh-Control cells (Figure S5C). When MCS was performed 36 h following DEX treatment (Figure 3F), the expression of *Per2* and *Cry1* in RAW264.7 with sh-Control 12 h after MCS (48 h following DEX treatment) was lower than in cells that had not undergone MCS (Figure 3G). In contrast, in RAW264.7 cells that underwent the same treatment, there was no effect of MCS on *Per2* and *Cry1* expression (Figure 3G). These findings suggest that MCS alters the macrophage circadian clock machinery by modulating *Per1* transcription.

### Impact of MCS on circadian regulation of macrophage phagocytosis

**Figure 4:**
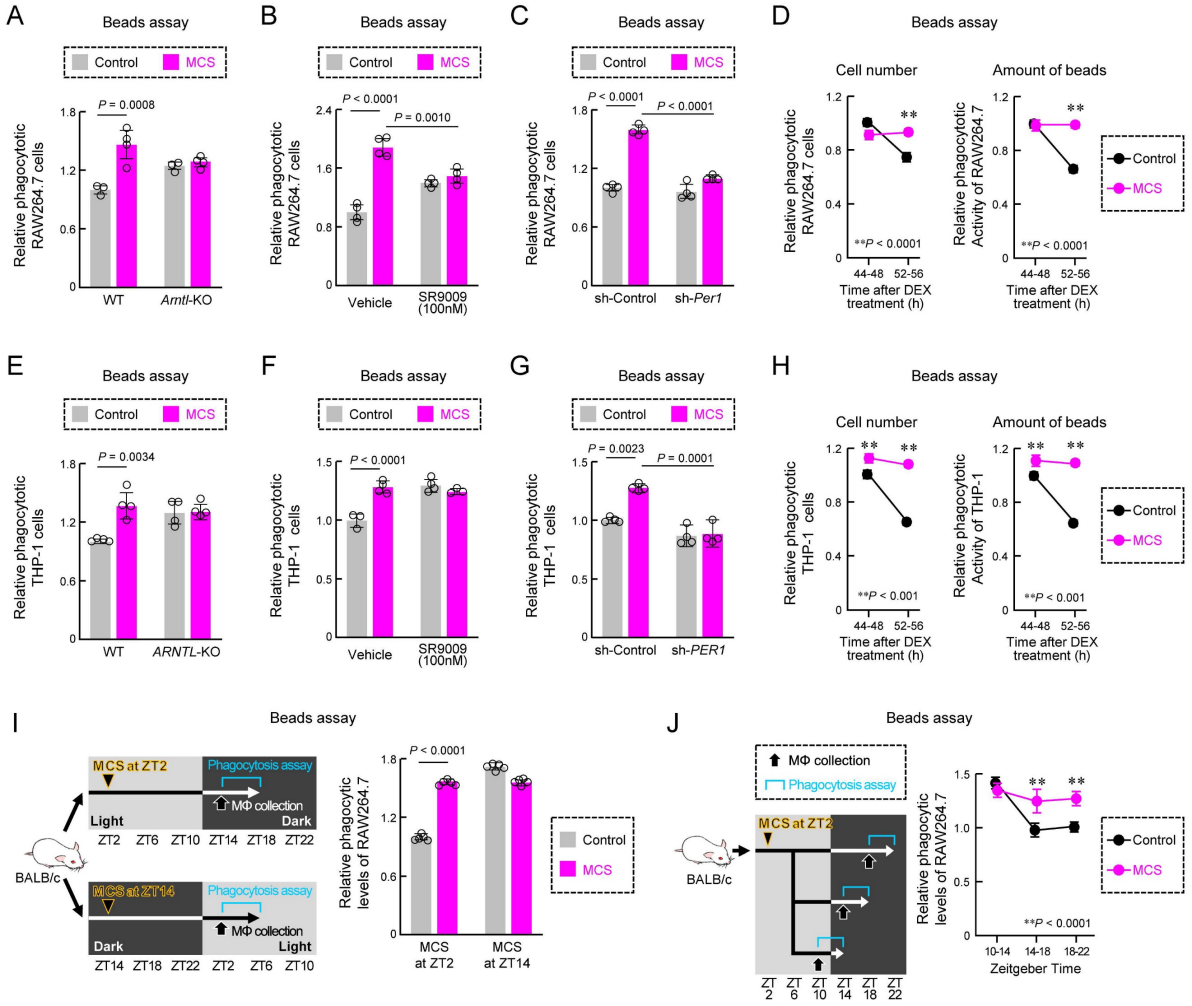
** Effects of MCS on the phagocytic activity via the circadian clock machinery.** (**A,B**) Effects of *Arntl* KO (**A**) and SR9009 exposure (**B**) on phagocytic activity of RAW264.7 cells using opsonized bead. The phagocytic activity was measured immediately after the addition of beads 12 h after MCS and co-incubated for another 3 h with or without SR9009 (100 nM). (**C**) Influence of sh-*Per1* lentivirus transduction on phagocytic activity of RAW264.7 cells. The phagocytic activity was measured immediately after the addition of beads 12 h after MCS and co-incubated for another 3 h. The expression of PER1 protein in the cells is illustrated in **Figure S5A**. (**D**) Effect of MCS on time-dependent decline in the number of phagocytic cells (left) and number of phagocytized beads per cell (right) in DEX-treated RAW264.7 cells. MCS was performed 24 h following treatment with 100 nM DEX for 2 h. The phagocytic activity was measured after beads were added 36 or 48 h following treatment with DEX and co-incubated for another 3 h. (**E,F**) Effects of *ARNTL* KO (**E**) and SR9009 exposure (**F**) on phagocytic activity of PMA-treated THP-1 cells using an opsonized bead. The phagocytic activity was measured immediately after the addition of beads 12 h after MCS and co-incubated for another 3 h with or without SR9009 (100 nM). (**G**) Influence of sh-*PER1* lentivirus transduction on phagocytic activity of PMA-treated THP-1 cells. The phagocytic activity was measured immediately after the addition of beads 12 h after MCS and co-incubated for another 3 h. The expression of PER1 protein in the cells is illustrated in **Figure S5**. (**H**) Effect of MCS on time-dependent decline in the number of phagocytic cells (left) and number of phagocytized beads per cell (right) in DEX-treated and PMA-treated THP-1 cells. MCS was performed 24 h after treatment with 100 nM DEX for 2 h. The phagocytic activity was measured after the addition of beads 36 or 48 h after DEX-treatment and co-incubated for another 3 h. (**I**) Stimulation time-dependent effect of MCS on phagocytic activity in peritoneal macrophages against opsonized beads. Phagocytosis activity was evaluated 12 h after MCS treatment. (**J**) Effect of MCS on the time-dependent decline in phagocytosis in peritoneal macrophages. Peritoneal macrophages were collected after 8 h of MCS, plated, and exposed to opsonized beads every 4 h to assess phagocytosis activity. Data are expressed as the mean ± S.D. (n = 4-5). Statistical significance was determined using two-way ANOVA with Tukey-Kramer post-hoc tests.* P*-values are shown in each graph. ANOVA: analysis of variance; DEX: dexamethasone; MCS: microcurrent stimulation; KO: knockout; RNA-seq: RNA sequencing; S.D.: standard deviation; ZT: Zeitgeber Time.

Next, we investigated how MCS influences the time-dependent decline in macrophage phagocytosis through the circadian clock mechanism. First, we examined the effects of ARNTL and NR1D1, clock genes related to phagocytosis 13,40, on the effects of MCS. By the knockout (KO) of *Arntl* and the exposure of SR9009, an agonist of the clock gene NR1D1 29, both *Arntl* KO and SR9009 exposure in RAW264.7 cells nullified the MCS-induced enhancement of phagocytosis (*P* < 0.01 for both, Figure 4A,B). Additionally, knockdown of *Per1*, a clock gene first affected by MCS, suppressed the MCS-induced increase in phagocytosis in RAW264.7 cells (*P* < 0.01, Figure 4C). Subsequently, we conducted a detailed exploration of the relationship between MCS effects and circadian fluctuations in phagocytic activity using DEX-treated RAW264.7 cells. RAW264.7 cells treated with MCS 36 h following DEX treatment did not exhibit the decline in phagocyte number and phagocytosis 44-56 h following DEX treatment seen in the control group (*P* < 0.01, Figure 4D). Furthermore, the same verification was conducted using PMA-treated THP-1. The results of *ARNTL*-KO (Figure 4E), SR9009 (Figure 4F), *PER1*-knockdown (Figures 4G, S6), and DEX treatment (Figure 4H) were the same as those of RAW264.7. These findings suggest that MCS augments macrophage phagocytosis via PER1 and the circadian clock mechanism.

We further investigated the relationship between circadian fluctuations in macrophage phagocytosis *in vivo* and the effect of MCS by applying stimulation at ZT2, consistent with previous studies, or ZT14, 12 h later. The impact of MCS was only evident with stimulation at ZT2 (*P* < 0.01, Figure 4I). Moreover, evaluation of the decline in phagocytosis during the dark period revealed that the decrease in macrophage phagocytosis in mice treated with MCS at ZT2 differed from that in the control group (*P* < 0.01, Figure 4J). These results suggest that MCS prevents the circadian reduction in phagocytosis.

**Figure 5:**
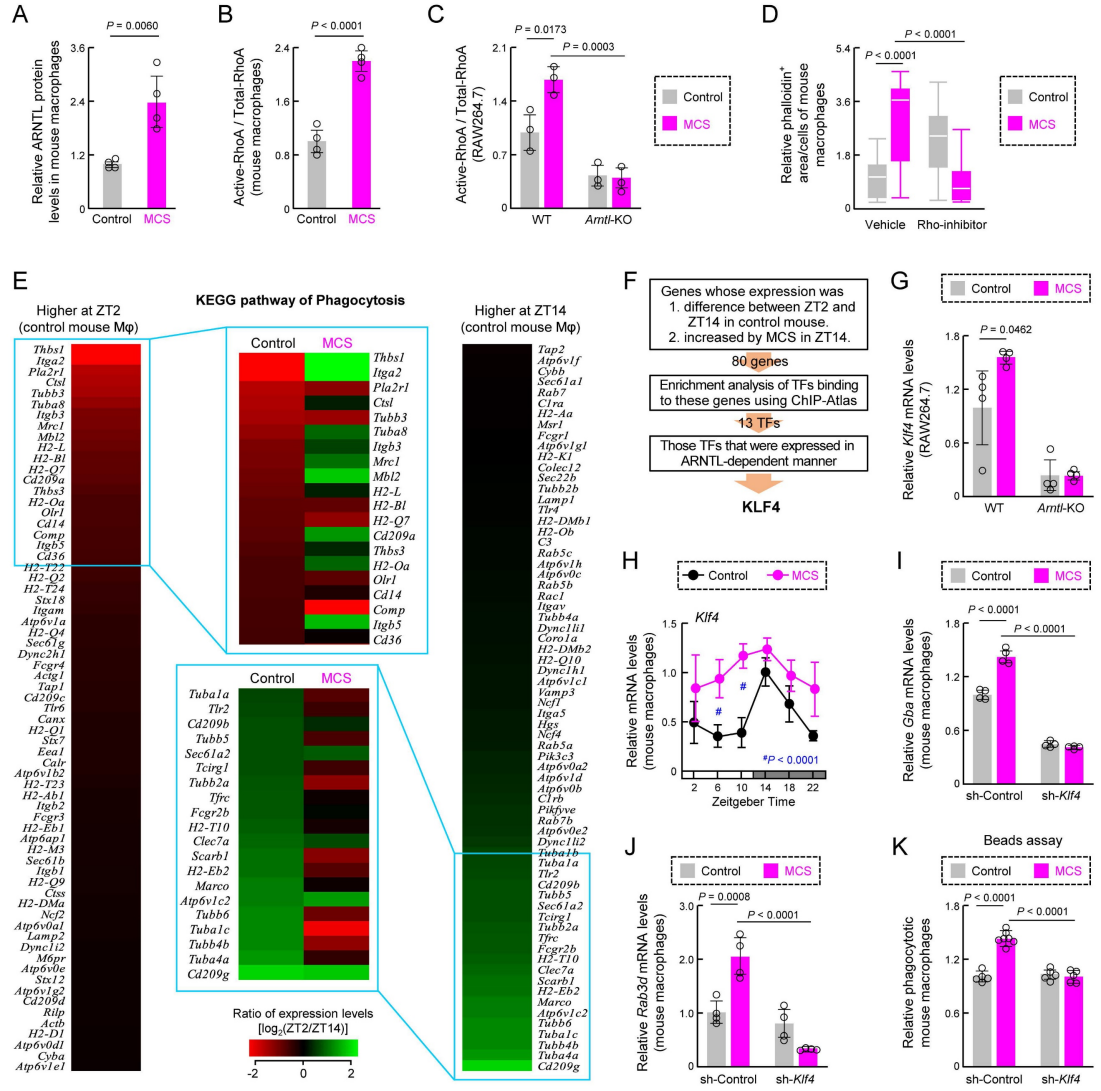
** Effects of MCS on the macrophage phagocytosis-related factors.** (**A**) The expression levels of ARNTL protein in intraperitoneal macrophages from Control or MCS-treated female BALB/c mice in ZT14. (**B**) The expression levels of active-RhoA protein in intraperitoneal macrophages from Control or MCS-treated female BALB/c mice in ZT14. (**C**) Effects of *Arntl* KO on RhoA activity of RAW264.7 cells. The expression of each protein was measured using cells 12 h after MCS. For panel A-C, the images of the western blots for each protein are shown in **Figure S7**. (**D**) Effects of Rho-inhibitor (1 µg/mL) on polymerized actin levels of intraperitoneal macrophages from Control or MCS-treated female BALB/c mice. The cells were exposed to the Rho inhibitor 8 h after MCS. Four hours after exposure to Rho-inhibitor, polymerized actin was stained with phalloidin. (**E**) Effect of MCS on time-dependent variations in phagocytosis-related gene expression in intraperitoneal macrophages, utilizing phagocytosis-related gene expression levels obtained from the RNA-seq results. The heatmaps depict genes with log (ZT2/ZT14) values above 0 (left) and below 0 (right) in control macrophages. The top 20 genes with the highest absolute values of each ratio were compared with the log (ZT2/ZT14) values of MCS macrophages, revealing reversed time-dependent variation for many genes. (**F**) Screening for a transcription factor (TF) that mediates the effects of MCS on genes whose expression levels are time-dependent. Using ChIP-Atlas enrichment analysis, we narrowed down the list of candidate TFs that bind to the region ±5,000 bp from the transcription start site of genes whose expression differed between ZT2 and ZT14 in control mice and increased by MCS in ZT14. Of these TFs, only KLF4 was expressed in mouse macrophages and affected by the loss of *Arntl.* The results of RNA-seq, ChIP-Atlas, and RT-qPCR used for this screening are shown in **Figure S8** and **Table S4**. (**G**) Effects of *Arntl* KO on the *Klf4* mRNA expression of RAW264.7 cells. The mRNA levels were measured 12 h after MCS. (**H**) Temporal mRNA expression profiles of *Klf4* in intraperitoneal macrophages from Control or MCS-treated female BALB/c mice. (**I,J**) The expression levels of *Gba* and *Rad3d* mRNA levels in the vehicle or sh-*Klf4* lentivirus transducted-mouse macrophages. The expression levels of KLF4 protein and *Klf4* mRNA in each cell are illustrated in **Figure S9**. (**K**) Influence of sh-*Klf4* lentivirus transduction on phagocytic activity of intraperitoneal macrophages from Control or MCS-treated female BALB/c mice. The phagocytic activity was measured immediately after the addition of beads 12 h after MCS and co-incubated for another 3 h. Data are expressed as the mean ± S.D. (D: n = 20-80, others: n = 4-6). Statistical significance was determined using two-way ANOVA with Tukey-Kramer post-hoc tests.* P*-values are shown in each graph. ANOVA: analysis of variance; DEX: dexamethasone; MCS: microcurrent stimulation; KO: knockout; RNA-seq: RNA sequencing; S.D.: standard deviation; ZT: Zeitgeber Time.

We also determined the phagocytosis-related factors whose function is altered by MCS through the clock genes. ARNTL regulates actin polymerization by binding to RhoA 13. MCS increased the levels of ARNTL protein and active RhoA in mouse macrophages (Figures 5A,B, S7). In addition, RhoA activation by MCS was not observed in *Arntl*-KO RAW264.7 cells (Figure 5C) and the addition of Rho-inhibitor (1 μg/mL) eliminated the increase in actin polymerization in mouse peritoneal macrophages caused by MCS (Figure 5D). Since PER1 reduces the expression and activity of ARNTL 17,18, these results suggest RhoA activity is regulated via ARNTL during the promotion of actin polymerization by MCS.

Phagocytosis by macrophages involves various genes in addition to actin polymerization.; therefore, we examined circadian variations in mRNA expression related to phagocytosis in mouse peritoneal macrophages. Phagocytosis by these macrophages displays circadian fluctuations, peaking during the light period and diminishing during the dark period 25,26. Analysis of RNA expression of phagocytosis-related genes using Kyoto Encyclopedia of Genes and Genomes (KEGG) (https://www.genome.jp/kegg/) database revealed differences between ZT2 and ZT14 in the control group (Figure 5E). Among the top 20 genes with high or low ZT2/ZT14 ratios, the ZT2/ZT14 ratio varied considerably in the MCS group (Figure 5E).

This suggests that changes in gene expression related to phagocytosis by MCS occur via transcription factors under the control of clock genes. We conducted a search for transcription factors capable of binding to the transcription start sites of the genes exhibiting differential expression between ZT2 and ZT14 in control mice and displaying an increase by MCS in ZT14 (Table S4), ±5000 bp using enrichment analysis of the ChIP-Atlas genomics database 41 (https://chip-atlas.org/). Of the screened transcription factors (Figure S8A), only Kruppel-like factor 4 (*Klf4*) was expressed in mouse macrophage RNA-seq (Figure S8B). Additionally, the expression level of *Klf4* in RAW264.7 cells was affected by the absence of ARNTL, a transcription factor involved in the circadian clock machinery (Figures 5F, S8C). *Klf4* is a transcription factor whose expression is regulated by ARNTL and is involved in regulating the circadian variation of macrophage phagocytosis 25. The expression level of *Klf4* mRNA increased after 12 h of MCS in wild-type RAW264.7 cells (Figure 5G), although not in *Arntl*-KO cells (Figure 5G). Furthermore, the expression of *Klf4* in the peritoneal macrophages of MCS-treated mice was significantly variable at multiple time points (*P* < 0.01 for ZT6 and 10, Figure 5H). Therefore, we suppressed *Klf4* expression in mouse peritoneal macrophages after MCS using anti-*Klf4* shRNA (sh-*Klf4*) (Figure S9). The MCS-induced increase in mRNA expression of glucosylceramidase beta (*Gba*) and RAB3D member RAS oncogene family (*Rab3d*), which are KLF4-dependent phagocytosis-related genes 25, was suppressed by sh-*Klf4* (Figure 5I,J). This also led to a decrease in the number of phagocytotic cells (Figure 5K). These findings indicate that MCS alters RhoA activity and gene expression associated with the circadian variation of macrophage phagocytosis *in vivo* and suppresses the decrease in time-dependent phagocytosis activity through these processes.

### MCS pre-treatment suppresses engraftment of peritoneally injected cancer cells

**Figure 6:**
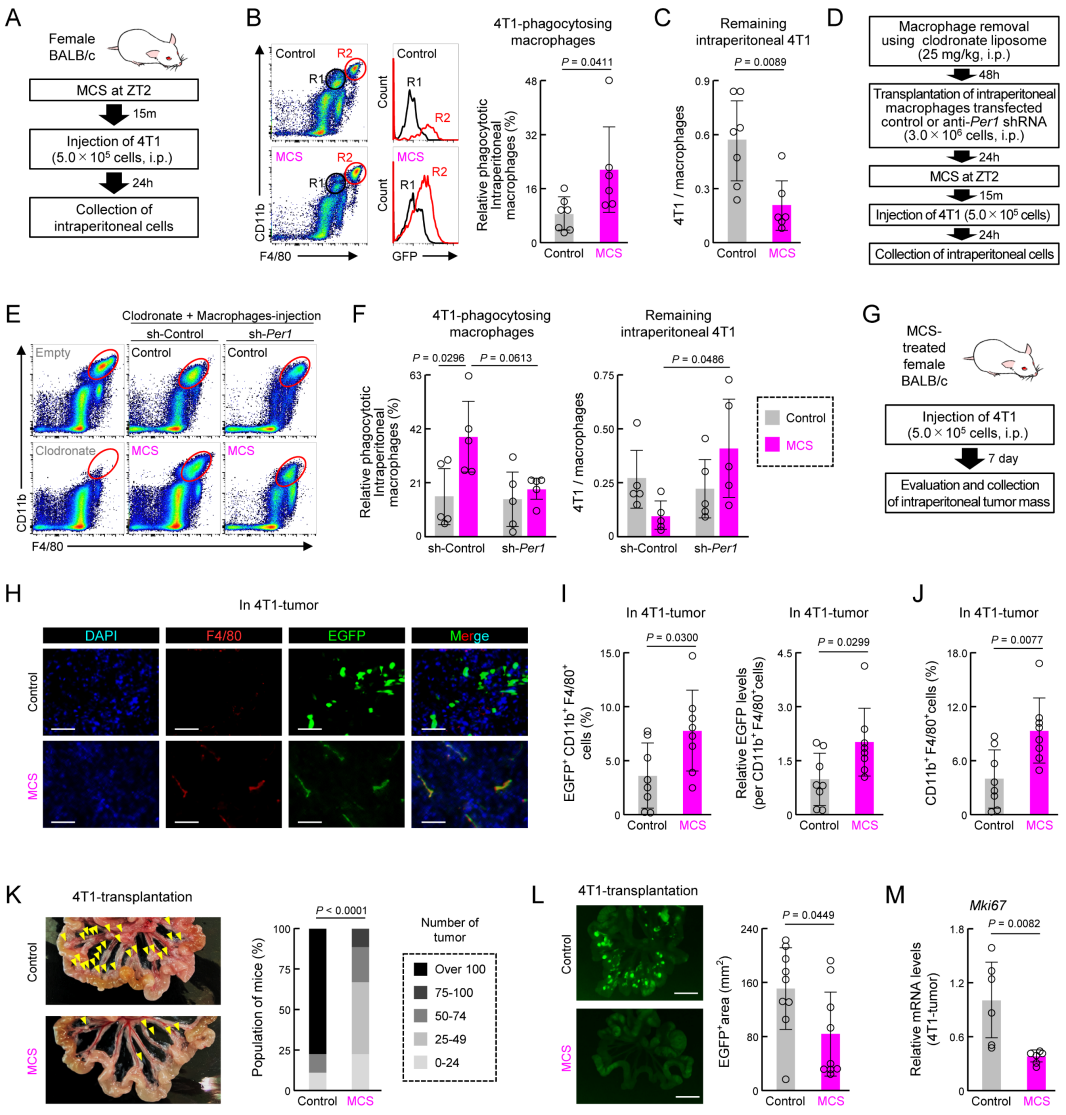
** Effect of prior MCS treatment on the viability of peritoneally seeded 4T1 cells.** (**A**) Protocol of MCS treatment and 4T1 cell injection. Female BALB/c mice received MCS treatment at ZT2 followed by intraperitoneal injection of 5.0 × 10^5^ GFP-expressing 4T1 cells. (**B**) Flow cytometry was utilized to assess the expression of macrophage markers CD11b and F4/80. GFP-positive macrophages, indicative of those phagocytosing 4T1 cells, were identified in the R2 region. The right panel illustrates the percentage of GFP positivity in macrophages (Ly6G^-^, CD11b^+^, F4/80^+^). The gating strategy is provided in **Figure S19**. (**C**) Counts of the 4T1 cells in collected intraperitoneal cells expressed as a ratio to total macrophages. (**D**) Protocol of macrophage removal, transplantation of *Per1*-downregulated macrophages, MCS treatment, and 4T1 cell injection. (**E,F**) Flow cytometry to assess CD11b and F4/80 expression (**E**), GFP positivity in macrophages (F; left), and counts of 4T1 cells (F; right) in collected intraperitoneal cells from *Per1*-downregulated macrophage-transplanted mice. (**G**) Protocol of 4T1 cell injection into MCS-treated mice and evaluation and collection of intraperitoneal tumor mass. (**H**) Immunohistochemistry image depicting the tumor mass formed around the portal vein. Nuclei were stained with DAPI, F4/80 with Red, and GFP represents 4T1 cells. Scale bar: 20 μm. (**I**) Ratio of GFP^+^ macrophages and the phagocytosed amount per cell in the tumor mass formed around the portal vein. (**J**) Total number of macrophages in the tumor. (**K**) Left: image of the removed small intestine. Yellow arrows indicate nodular tumors formed around the portal vein. Right: Number of nodular tumors formed in the abdominal cavity. Mice are ranked according to the number of tumors, with the percentage of mice in each rank depicted for the control and MCS groups, respectively. (**L**) 4T1-derived GFP fluorescence around the portal vein. The right panel illustrates the proportion of the GFP-positive area. Scale bar: 10 mm. (**M**) Difference in the expression of *Mki67* mRNA in tumors between the control and MCS-treated groups. Data are expressed as the mean ± S.D. (n = 4-7). Statistical significance was determined using two-way ANOVA with Tukey-Kramer post-hoc tests (**f**) or two-tailed Student's* t*-tests (**B,C**, **I-M**).* P*-values are shown in each graph. ANOVA: analysis of variance; DAPI: 4′,6-diamidino-2-phenylindole; GFP: green fluorescent protein; MCS: microcurrent stimulation; S.D.: standard deviation; ZT: Zeitgeber Time.

We next investigated how MCS-induced activation of macrophage phagocytosis affects cancer cell engraftment and tumorigenesis. Twenty-four hours post-injection of mouse breast cancer cell line 4T1 into the abdominal cavity of female mice treated with MCS at ZT2 (Figure 6A), the percentage of abdominal macrophages engulfing 4T1 cells was significantly higher than that in the control group (*P* < 0.05, Figure 6B). Additionally, the number of remaining 4T1 cells in the abdominal cavity was significantly reduced (*P* < 0.01, Figure 6C). To determine whether the clock genes mediate this MCS-dependent increase in phagocytosis and decrease in 4T1 cell number, intraperitoneal macrophages were collected from healthy mice, and cells were infected with lentivirus-expressing shRNA against *Per1*. Control or *Per1*-downregulated macrophages were injected intraperitoneally into mice administered with clodronate liposome, which removed macrophages (Figure 6D,E). Following MCS treatment and transplantation of 4T1 cells into these mice, neither the increase in phagocytosis of macrophages by MCS nor the decrease in the number of remaining 4T1 cells was observed in mice transplanted with *Per1*-downregulated macrophages (Figure 6F).

Notably, 4T1 injection into the peritoneum resulted in multiple nodular tumors around the portal vein after 7 days (Figure S10). To assess the effect of MCS on tumor formation, MCS treatment at ZT2 was followed by 4T1 injection, and tumors and macrophages within the tumors were observed 7 days post-transplantation (Figure 6G). Immunohistochemical staining of tumor sections revealed that only in the MCS group, F4/80-positive cells within the tumor exhibited green fluorescence derived from 4T1 cells (Figure 6H). Moreover, flow cytometry analysis of tumor macrophages (CD11b^+^, F4/80^+^, Ly6G^-^ cells) showed a significant increase in both the number of GFP-positive cells and the GFP intensity per cell following MCS (*P* < 0.05 for both, Figure 6I). Furthermore, MCS augmented the number of macrophages within the tumors (*P* < 0.01, Figure 6J). The occurrence of nodular tumors around the portal vein (*P* < 0.01, Figure 6K) and the total area of tumor-derived fluorescence was significantly reduced by MCS (*P* < 0.05, Figure 6L). Additionally, the expression of *Mki67*, indicative of breast cancer tumor malignancy grade 42, was significantly decreased in tumors following MCS treatment (*P* < 0.01, Figure 6M).

**Figure 7:**
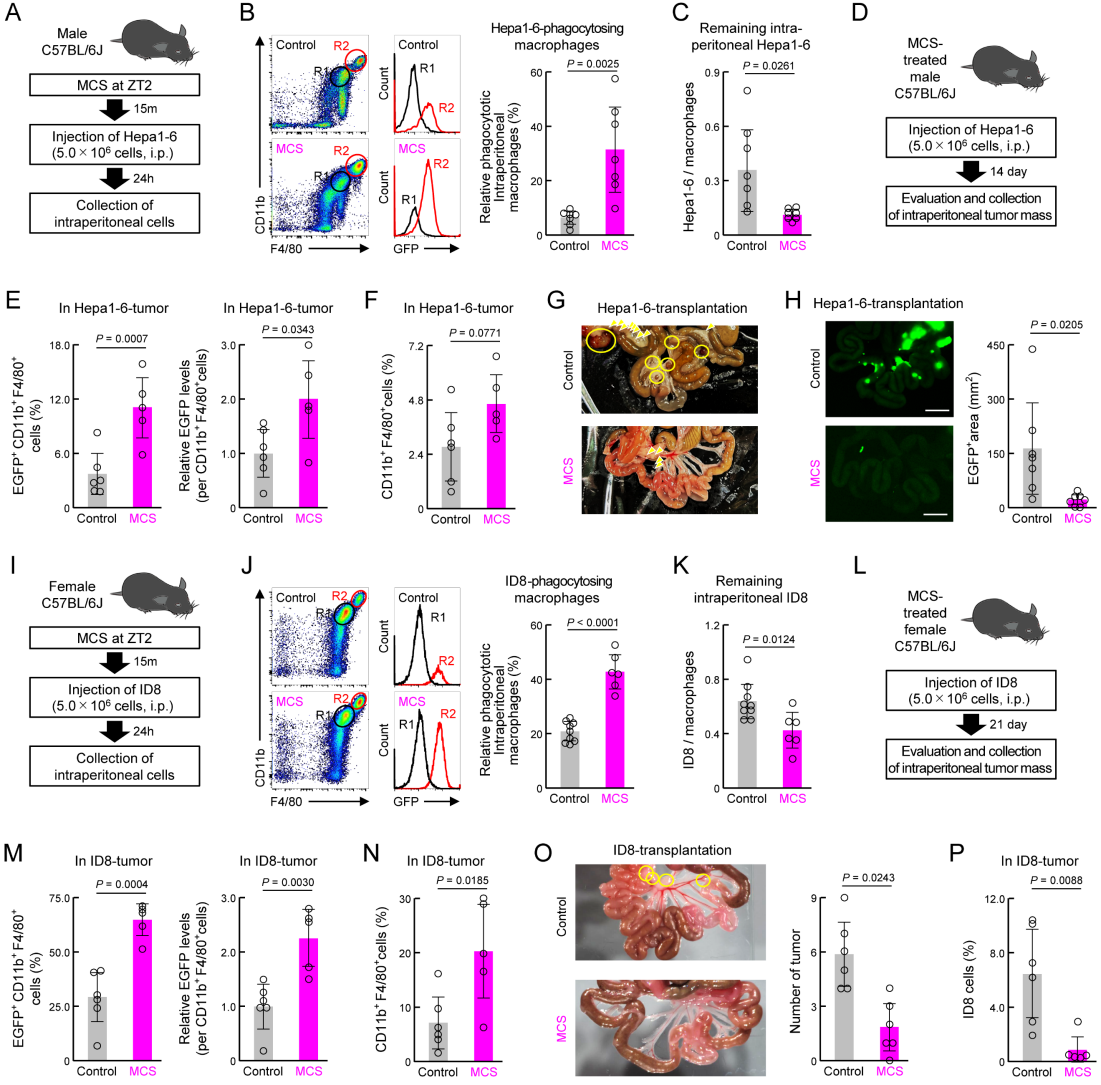
** Effect of prior MCS treatment on the viability of peritoneally seeded Hepa1-6 and ID8 cells.** (**A**) Protocol of MCS treatment and Hepa1-6 cell injection. Male C57BL/6J mice received MCS treatment at ZT2 followed by intraperitoneal injection of 5.0 × 10^6^ GFP-expressing Hepa1-6 cells. (**B**) Flow cytometry was utilized to assess the expression of macrophage markers CD11b and F4/80. GFP-positive macrophages, indicative of those phagocytosing Hepa1-6 cells, were identified in the R2 region. The right panel illustrates the percentage of GFP positivity in macrophages (Ly6G^-^, CD11b^+^, F4/80^+^). The gating strategy is provided in **Figure S19**. (**C**) Counts of Hepa1-6 cells in collected intraperitoneal cells presented as a ratio to total macrophages. (**D**) Protocol of Hepa1-6 cell injection into MCS-treated male C57BL/6J mice and evaluation and collection of intraperitoneal tumor mass. (**E**) The ratio of GFP^+^ macrophages and the phagocytosed amount per cell in the tumor mass formed around the portal vein. (**F**) Total number of macrophages in the tumor. (**G**) Image of the removed small intestine of Hepa1-6-transplanted male mice. Yellow arrows indicate nodular tumors and yellow circles indicate tumors with visible capillaries. (**H**) Hepa1-6-derived GFP fluorescence around the portal vein in male mice. The right panel shows the proportion of the GFP-positive area. Scale bar: 10 mm. (**I**) Protocol of MCS treatment and ID8 cell injection. Female C57BL/6J mice received MCS treatment at ZT2 followed by intraperitoneal injection of 5.0 × 10^6^ GFP-expressing ID8 cells. (**J**) Flow cytometry was utilized to assess the expression of macrophage markers CD11b and F4/80. GFP-positive macrophages, indicative of those phagocytosing ID8 cells, were identified in the R2 region. The right panel illustrates the percentage of GFP positivity in macrophages (Ly6G^-^, CD11b^+^, and F4/80^+^). The gating strategy is provided in **Figure S19**. (**K**) Counts of ID8 cells in collected intraperitoneal cells presented as a ratio to total macrophages. (**L**) Protocol of ID8 cell injection into MCS-treated female C57BL/6J mice and evaluation and collection of intraperitoneal tumor mass. (**M**) Ratio of GFP^+^ macrophages and the phagocytosed amount per cell in the tumor mass formed around the portal vein. (**N**) Total number of macrophages in the tumor. (**O**) Left: Photographic image of the removed small intestine of ID8-transplanted female mice. Yellow circles indicate nodular tumors. Right: The number of the tumor mass formed around the portal vein. (**P**) The number of ID8 cells as a percentage of the total number of cells in the tumor mass, as measured by flow cytometry. Data are expressed as the mean ± S.D. (n = 4-7). Statistical significance was determined using two-tailed Student's *t*-tests.* P*-values are shown in each graph. GFP: green fluorescent protein; MCS: microcurrent stimulation; S.D.: standard deviation; ZT: Zeitgeber Time.

Subsequently, we validated these findings using the mouse liver cancer cell line Hepa1-6 in male mice (Figure 7A). Similar to that observed for 4T1, MCS in male mice enhanced the phagocytic capacity of peritoneal macrophages toward Hepa1-6 cells injected into the peritoneal cavity and reduced the number of remaining Hepa1-6 cells in the abdominal cavity (*P* < 0.05 for both, Figure 7B,C). Evaluation of the post-tumor implantation status under the same conditions as with 4T1 (Figure 7D) revealed that MCS increased cancer cell phagocytosis by tumor macrophages and macrophage infiltration (Figure 7E,F). Furthermore, the MCS group exhibited no tumor mass formation (Figure 7G) and a significantly reduced total tumor area (*P* < 0.05, Figure 7H) compared with the control group.

Additional validation was conducted using ID8 mouse ovarian surface epithelial cells, which have the ability to metastasize to the peritoneum 43, in female mice (Figure 7I). MCS in female mice enhanced the phagocytic capacity of peritoneal macrophages toward ID8 cells injected into the peritoneal cavity and reduced the number of remaining ID8 cells in the abdominal cavity (*P* < 0.05 for both, Figure 7J,K). Post-tumor implantation status evaluation under the same conditions as those with 4T1 (Figure 7L) revealed that MCS increased cancer cell phagocytosis by tumor macrophages and macrophage infiltration (Figure 7M,N). Furthermore, the MCS group had significantly fewer tumor masses formed in the abdominal cavity (*P* < 0.05, Figure 7O), and the percentage of ID8 cells in this tumor mass was significantly reduced (*P* < 0.01, Figure 7P) compared with the control group. These findings suggest that enhancing macrophage phagocytic capacity via MCS suppresses cancer cell engraftment in the abdominal cavity and reduces tumor size in both male and female mice.

### MCS induces cancer immunity activation following peritoneal seeding

**Figure 8:**
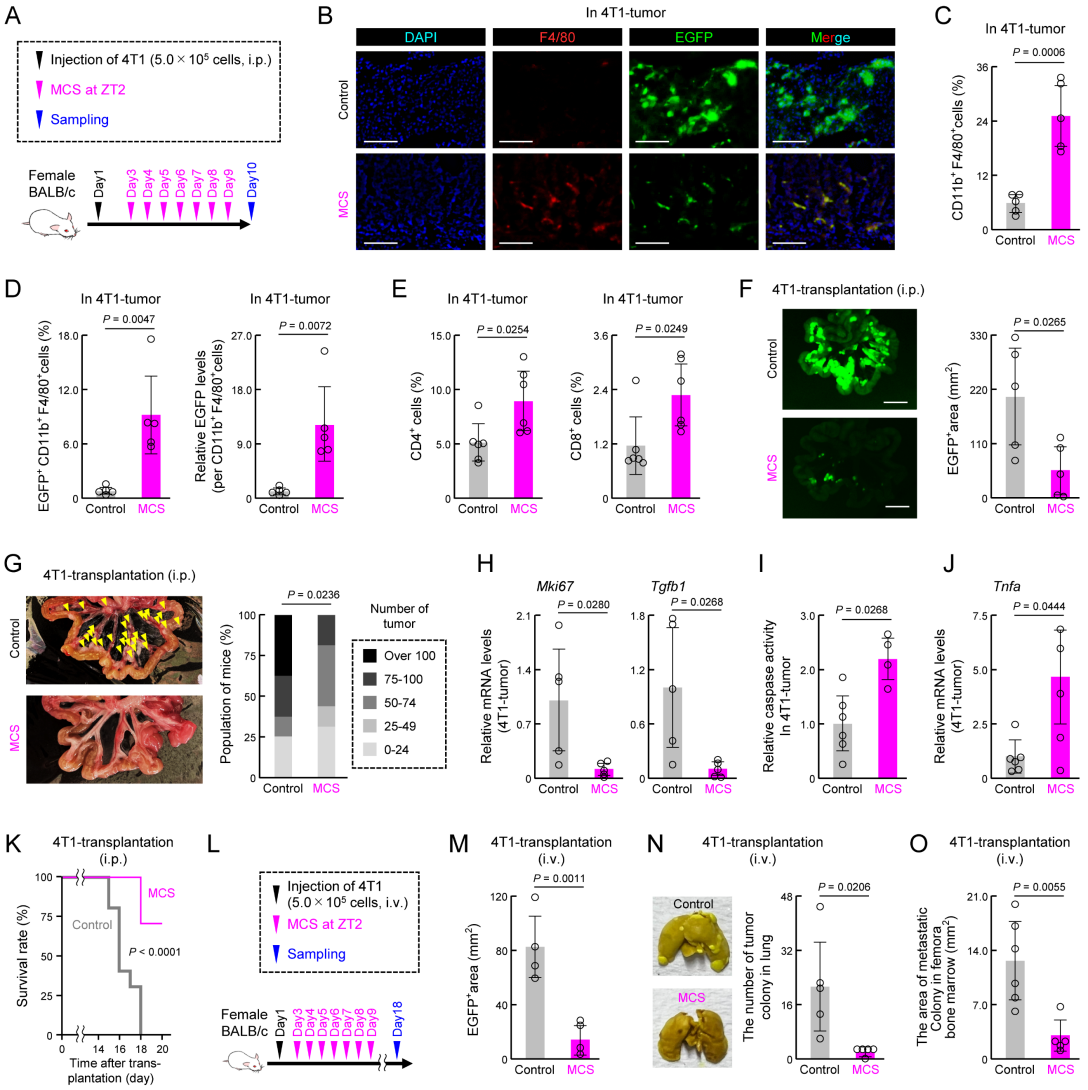
** Effect of MCS on tumor development, immune response, metastasis, and survival rate.** (**A**) MCS treatment protocol in BALB/c mice intraperitoneally injected with 4T1 cells. MCS was administered daily for 1 week starting from the second day post-injection. (**B**) Immunohistochemistry image depicting tumor mass formation around the portal vein. Nuclei are stained with DAPI (blue), F4/80-positive macrophages are stained in red, and GFP indicates 4T1 cells. Scale bar: 20 μm. (**C**) Total macrophage count within tumors around the portal vein. (**D**) Ratio of GFP-positive macrophages and the amount of phagocytosed material per cell within tumors. (**E**) Ratio of infiltrated CD4^+^ or CD8^+^ T cells (CD3^+^ CD19^-^) within tumors. (**F**) Visualization of 4T1-derived GFP fluorescence around the portal vein. The right panel shows the proportion of the GFP-positive area. Scale bar: 10 mm. (**G**) Left: Image of excised small intestine with yellow arrows indicating nodular tumors around the portal vein. Right: Number of nodular tumors formed in the abdominal cavity. Each mouse is ranked based on tumor count, and the percentage of mice in each rank is presented for control and MCS groups. (**H**) Differential *Mki67* and *Tgfβ1* mRNA expression in tumors between control and MCS-treated groups. (**I**) Difference in caspase-3/7 activity between control and MCS-treated tumors. (**J**) Difference in *Tnfa* mRNA expression in tumors between control and MCS-treated groups. (**K**) Kaplan-Meier survival curves of control and MCS-treated mice intraperitoneally injected with 4T1 cells. (**L**) MCS treatment protocol in BALB/c mice injected with 4T1 cells via the tail vein. MCS was administered daily for 1 week starting from the second day post-injection. For panels M-O, the mice were used 18 days after 4T1 transplantation. (**M**) The 4T1-derived GFP-positive area around the portal vein in control and MCS-treated mice injected with 4T1 via the tail vein. Representative images are shown in **Figure S11A**. (**N**) The number of pulmonary tumor colonies in control and MCS-treated mice injected with 4T1 via the tail vein. The left panels show representative images of pulmonary tumor colonies. The right panel shows the quantification of the number of tumor colonies in the lungs. (**O**) The quantification of the area of metastatic colonies isolated from tumor-bearing mice femora bone marrow. Representative photographs of tumor colonies are shown in **Figure S11B**. Data are expressed as mean ± S.D. (n = 4-7). Statistical significance was determined using two-tailed Student's *t*-tests.* P*-values are shown in each graph. ANOVA: analysis of variance; DAPI: 4′,6-diamidino-2-phenylindole; GFP: green fluorescent protein; MCS: microcurrent stimulation; S.D.: standard deviation.

Next, we examined the impact of MCS on cancer immunity and tumorigenesis when MCS was administered 2 days post intraperitoneal injection of 4T1 cells, facilitating cancer cell engraftment (Figure 8A). Tumors from the MCS group exhibited a notable increase in the number of F4/80-positive cells compared with that in the control group, indicating enhanced macrophage infiltration (Figure 8B). Moreover, GFP-derived fluorescence, indicative of 4T1 cells, was diminished in tumors from the MCS group, with few GFP-positive cells observed (Figure 8B). Flow cytometry analysis revealed higher total macrophage numbers within tumors (*P* < 0.01, Figure 8C), along with an elevated percentage of GFP-positive macrophages (*P* < 0.01, Figure 8D, left) and increased GFP-derived fluorescence intensity per cell (*P* < 0.01, Figure 8D, right) in the MCS group. Additionally, MCS significantly augmented the presence of CD4^+^ and CD8^+^ lymphocytes within tumors (*P* < 0.05 for both, Figure 8E). Tumor growth was significantly attenuated in the MCS group, evidenced by reduced periportal tumor area (*P* < 0.05, Figure 8F) and fewer nodular tumors (*P* < 0.05, Figure 8G). Concomitantly, MCS decreased *Mki67* and *Tgfβ1* expression in tumors (*P* < 0.05 for both, Figure 8H), indicative of suppressed proliferation and reduced immunosuppression. Moreover, tumors in the MCS group exhibited increased caspase activity and *Tnfα* expression, suggesting enhanced tumor cell death (*P* < 0.05 for both, Figure 8I,J). MCS significantly improved the survival rate after 4T1-transplantation (*P* < 0.01, Figure 8K). These findings demonstrate that MCS exerts a tumor-suppressive effect by activating cancer immunity mechanisms, leading to improved prognosis.

Additionally, since 4T1 shows metastatic potential to the lungs and bone marrow through injection into the tail vein 44,45, we investigated the effect of MCS on the metastatic potential of cancer cells using 4T1 transplanted mice with tail vein injection (Figure 8L). Mice injected with 4T1 cells and exposed to MCS exhibited limited tumor colony formation around the portal vein (*P* < 0.01, Figures 8M, S10A), lung (*P* < 0.05, Figure 8N), and bone marrow (*P* < 0.01, Figures 8O, S10B) compared with the control group. These findings suggest that MCS can reduce tumor engraftment, growth, and metastasis, and improve prognosis.

## Discussion

The circadian rhythms governing physiological functions, along with the circadian clock machinery orchestrating them, are pivotal factors influencing the function of immune cells involved in cancer immunity 19,20. Considering the relevance of the circadian clock machinery to macrophage and T cell functions 14 and the correlation between the efficacy of cancer immunotherapy and administration timing 27, targeting the circadian clock mechanism of immune cells holds promise for enhancing cancer immunotherapy efficacy. In this study, we demonstrated that manipulating macrophage clock gene expression through MCS augmented macrophage phagocytic activity against cancer cells, leading to inhibition of cancer cell engraftment and tumor growth. Specifically, MCS administered to mouse abdomen hindered the peri-intestinal engraftment and proliferation of breast cancer cells (4T1). Breast cancer, known for its relatively low response rates to T cell ICIs and high CD47 expression 9-11, has been challenging to treat with immunotherapy. The observed increase in macrophage phagocytosis and tumor-infiltrating CD4^+^ and CD8^+^ cells in breast cancer cells suggests that altering macrophage clock gene expression via MCS could enhance the antitumor activity of immune cells against breast cancer. Manipulating the macrophage circadian clock mechanism thus emerges as a promising therapeutic strategy for cancers with limited responsiveness to immunotherapy.

MCS applied to RAW264.7 and THP-1 macrophage cell lines in this study enhanced their phagocytic capacity against various solid tumor-derived cancer cells, encompassing those of breast, melanoma, liver, colon, lung, glioblastoma, renal, ovarian, and pancreatic cancer. Conversely, MCS-treated macrophage cell lines did not affect the viability of non-tumor-derived cell lines such as NIH3T3 and astrocytes. Since the phagocytosis of cancer cells by phagocytic cells involves the inhibitory SIRPα-CD47 interaction and the facilitatory LRP-cancer cell membrane CALR interaction, blockade of the LRP-CALR interaction impedes phagocytosis 46,47. The expression levels of CD47 in the cell lines used in this study were generally lower in the tumor-derived cell lines than in the non-tumor-derived cell lines (Figure S12, left). Of these, NIH3T3 cells, which are the non-tumor-derived cell line and express the highest levels of CD47, exhibit minimal phagocytosis by RAW264.7 cells, and the effects of MCS and the anti-CD47 antibody magrolimab were also not observed (Figure S13A-C). In contrast, the expression levels of CALR in the cell lines used in this study were generally higher in the tumor-derived cell lines than in the non-tumor-derived cell lines (Figure S12, right). When the expression of *Calr* in 4T1 cells, which is derived from breast cancer, was knocked down, the phagocytic activity of RAW264.7 cells towards 4T1 cells was significantly reduced, and abolished the effect of MCS (Figure S13D,E). This suggests that MCS selectively influences cancer cells expressing high levels of CALR without affecting the self-nonself recognition system mediated by SIRPα-CD47 and LRP-CALR interaction in macrophages. This is considered to be the underlying cause of MCS refraining from interfering in non-cancer cells that are protected from phagocytosis by these interactions. Further investigation of the effects of MCS on tumorigenesis of human cancer cells is needed to gain deeper insights into the potential application of MCS for cancer immunotherapy. However, it is important to note that many immunocompromised mice used in xenograft models have abnormalities in immune cells in general, including macrophages and lymphocytes.

Moreover, MCS did not induce alterations in M1/M2 differentiation markers such as plasma membrane CD11c, CD206, and *Tnfα* mRNA in macrophages. *Arntl* KO or exposure to SR9009 in RAW264.7 cells enhanced phagocytosis and abolished the effect of MCS, indicating that the MCS-induced increase in macrophage phagocytic activity is independent of macrophage differentiation induction. This is consistent with previous findings demonstrating that *Arntl* loss in macrophages does not affect M1/M2 differentiation 48 and that *Arntl* KO enhances macrophage phagocytosis 13, along with SR9009-induced phagocytosis in retinal cells 40. Additionally, MCS-induced upregulation of the expression of *Klf4*, a regulator of circadian variation in macrophage phagocytosis whose expression is modulated by *Arntl* 25,49, and its-dependent activation of phagocytosis activity suggests that MCS influences the expression of *Klf4* and other molecules involved in circadian variation in phagocytosis through the circadian clock mechanism. In addition to *Gba* and *Rab3d*, the association of phagocytosis-related genes such as *Thbs1* and *Tubulin* with clock genes and KLF4 further supports the circadian clock-dependent action of MCS 50,51. RhoA, which controls actin reorganization, is also closely related to clock genes, and the expression of CLOCK and ARNTL affects actin polymerization and phagocytic activity via RhoA activity in various cells 13. The activation of phagocytosis by MCS may involve a combination of an increase in F-actin due to the activation of RhoA via the stabilization of ARNTL caused by a decrease in *Per1* expression and an increase in the expression of phagocytosis-related genes due to the upregulation of *Klf4*.

MCS administered under the same conditions as those in this study affects *Per1* expression in cells, such as NIH3T3 cells and astrocytes, and organs, such as the liver, both *in vitro* and *in vivo* 31. The significant alteration of *Per1* mRNA expression in abdominal macrophages at ZT2 immediately post-MCS and the abrogation of MCS-induced increase in phagocytic activity upon suppression of *Per1* expression using sh-*Per1* in RAW264.7 cells suggested that MCS exerts its effects on macrophages through *Per1*, consistent with previous findings 31. In the suprachiasmatic nucleus, CREB, activated by retinal nerve firing, synchronizes the body clock with the external light-dark cycle by transiently increasing *Per1* expression and suppressing E-box-regulated genes, including *Per* and *Cry* 52. It is plausible that MCS-induced alteration of ZT14, the time of peak *Per1* mRNA expression in macrophages, operates via a similar mechanism. Conversely, since the circadian periodicity of clock gene expression persists unless *Per1*, *Per2*, and *Per3* are collectively lost, changes in *Per1* expression minimally impact the circadian periodicity of other clock gene expressions under light/dark cycle conditions 53,54. Whereas MCS-induced changes in the timing of peak *Per1* expression were observed, RT-qPCR and RNA-seq did not reveal similar alterations in other clock genes, suggesting that MCS primarily influences *Per1* expression with minimal impact on the circadian periodicity of clock gene expression. However, given that macrophage circadian clock dysregulation exacerbates conditions such as cardiac fibrosis and multiple sclerosis 23,24, the effects of MCS intervention on macrophage circadian clock in non-cancerous diseases warrant careful investigation.

As current stimulation below 300 μA influences various chronic wounds via macrophages 32,36, evidence of MCS effects on immune function continues to emerge. In this study, MCS impeded the growth and proliferation of cancer cells pre-transplantation in both male and female mice, where three types of cancer cells, 4T1, Hepa1-6, and ID8, were transplanted, highlighting a significant finding concerning the interplay between macrophages and MCS. Notably, electrical stimulation under conditions different from those in this study enhances NF-κB signaling and nitric oxide production in macrophages, which may also relate to the circadian clock-dependent effect of MCS on phagocytosis 32,36.

Moreover, metal ion influx into cells 55,56 and modulation of protein kinases 31,57 associated with electrical stimulation have links to clock genes 58, potentially mediating MCS-induced changes in *Per1* expression. The fact that MCS decreased the expression level of* Per1* in ARNTL-KO RAW264.7 cells (Figure S14A,B) and that MCS did not change the expression level of ARNTL in *Per1*-knockdown RAW264.7 cells (Figure S14C) suggested that MCS increases the expression level of ARNTL via the downregulation of *Per1* expression. The fluctuation in Per1 expression caused by MCS relates with the phosphorylation of cAMP response element binding protein (CREB) by protein kinase A (PKA) 31. MCS to the mouse abdomen decreased the level of phosphorylated CREB in macrophages (Figure S14D), and exposure to the PKA inhibitor H89 or the divalent metal ion influx inhibitor EDTA abolished the effects of MCS on *Per1* expression and phagocytosis in RAW264.7 cells (Figure S14E,F). PERIOD, including PER1, and CRY, which form heterodimers with PERIOD, control the transcription, function and degradation of the CLOCK/ARNTL complex 17,18,59,60. In addition, the reduction in Per gene expression caused by mechanical stress reduces ARNTL 61. Therefore, our results suggest that MCS modulates *Per1* expression via the divalent metal ion-cAMP-PKA-CREB pathway and that the stabilization of ARNTL protein by this mechanism is involved in MCS-induced phagocytosis (Figure S14G). The capacity of *in vivo*-generated electric fields and electrical stimulation to modulate cell movement by fostering intercellular pore formation 62 aligns with our findings of MCS augmenting macrophage phagocytosis and tumor invasion. Consequently, the circadian clock-mediated mechanism unveiled in this study may be part of the mechanism through which cell motility is influenced by electrical stimulation, warranting further exploration.

The MCS protocol employed in this study resembles pulse depolarization iontophoresis, which, unlike the conventional continuous, direct current, poses less harm to living tissue 63. Iontophoresis has been utilized not only for its inherent therapeutic benefits but also for facilitating drug delivery to the skin 63. Numerous studies have explored the relationship between electrical stimulation conditions and skin physiology, revealing that higher MCS frequencies correlate with decreased skin impedance, whereas impedance rises with constant current intensity 63-65. However, the mechanisms underlying MCS effects on tissues other than the skin remain to be fully elucidated. Visualizing electrons entering the body through the skin remains challenging, making it difficult to pinpoint the initial molecular events of MCS within intracellular molecules, including macrophages. Nevertheless, comprehensive analyses akin to ours are pivotal to a deeper understanding of these mechanisms.

MCS administered after 4T1 transplantation augmented the presence of macrophages, CD4^+^, and CD8^+^ T cells within tumors. Given that phagocytosis triggers tumor-T cellular immunity through cytokine secretion and antigen presentation 66, it is plausible that MCS activated T cellular immunity via these macrophage functions. Evidence indicating minimal circadian variation in T cell clock gene expression compared with that in other cells, including macrophages, and the minor role of T cell clock gene expression in T cell function 14,67,68 supports the notion that macrophages mediate MCS-induced T cell immune activation. This finding is reinforced by the results demonstrating that the depletion of macrophages by clodronate liposomes eliminated the tumor-suppressing effect of MCS on 4T1-transplanted mice (Figure S15A-C). MCS slightly enhanced the phagocytic activity of neutrophils in some tumor-bearing mice (Figure S16A,B) but did not induce phagocytosis to the same extent as macrophages (Figure S16C,D), since macrophages have a greater phagocytic capacity for large foreign bodies such as cancer cells and apoptotic cells 69. This is further supported by the fact that the effect of MCS disappeared with the addition of clodronate liposomes, which had a limited effect on neutrophils compared with macrophages (Figure S15D).

Since phagocytosis by macrophages and T-cell immunity have antitumor effects on solid tumors, MCS can potentially treat such cancers. The observed effect of electrical stimulation on chronic wounds, which enhances fibroblast proliferation 70,71, might also contribute to MCS action on tumors by promoting the formation of the tumor microenvironment. Notably, tumor-associated macrophages (TAMs) have the capacity to differentiate into either tumor-promoting or -suppressing cells based on the surrounding milieu, governed by the tumor microenvironment comprising the extracellular matrix and immune cells 72. Although our study revealed that MCS did not directly induce macrophage differentiation into CD206-positive cells (i.e., tumor-promoting M2-like TAMs), this differentiation may be indirectly influenced by the complex interplay among cancer cells, fibroblasts, and capillaries within the tumor microenvironment. Given that extracellular matrix proliferation, certain TAM types, and neovascularization promote tumor growth 73, it is important to carefully evaluate the effects of MCS on solid tumors. We attempted to apply MCS to mice with solid tumors, such as the footpads or dorsal-subcutaneous transplantation; however, it was extremely difficult to attach the stimulation pad to areas with a small surface area and many irregularities, such as the footpads or dorsal where the tumor had engrafted. Therefore, we could not achieve this. To overcome the limitations of this research, it is imperative to verify the findings using larger animals or humans. Alternatively, since the equipment used for MCS in this study is suitable for humans who are typically large with minimal body hair, it may be beneficial to develop equipment suitable for mice, which are small and possess dense fur, in order to advance basic research.

## Methods

### Cell culture and treatment

RAW264.7 mouse macrophage-like cells (RRID: CVCL_0493), THP-1 human monocyte-like cells (RRID: CVCL_0006), 4T1 mouse breast cancer cells (RRID: CVCL_0125), MDA-MB-231 human breast cancer cells (RRID: CVCL_0062), MCF-7 human breast cancer cells (RRID: CVCL_ 0031), A549 human lung cancer cells (RRID: CVCL_ 0023), U251-MG human glioblastoma (RRID: CVCL_ 0021), MiaPaCa2 human pancreatic cancer cells (RRID: CVCL_ 0428), and PANC-1 human pancreatic cancer cells (RRID: CVCL_0480) were purchased from American Type Culture Collection (Manassas, VA, USA). NIH3T3 fibroblasts (RRID: CVCL_0594), Hepa1-6 mouse hepatocytes (RRID: CVCL_0327), RenCa mouse renal carcinoma (RRID: CVCL_ 2174), and B16 mouse melanoma (RRID: CVCL_0157) were purchased from Cell Resource Center for Biomedical Research (Tohoku University, Miyagi, Japan). Colon26 mouse colon carcinoma (RRID: CVCL_0240) were purchased from Cell Bank Riken BioResource Center (Ibaraki, Japan). ID8 mouse ovarian surface epithelial cells (RRID: CVCL_ IU14) were purchased from Sigma Aldrich (St. Louis, MO, USA). Mouse astrocytes and mouse embryonic fibroblasts (MEFs) were established following procedures similar to those reported in previous studies 74,75.

RAW264.7, MDA-MB-231, Hepa1-6, B16, MCF-7, A549, U251-MG, RenCa, and NIH3T3 cells; astrocytes; MEFs were cultured in Dulbecco's modified Eagle's medium (DMEM) supplemented with 5% fetal bovine serum (FBS) and 0.5% penicillin-streptomycin solution (Invitrogen Life Technologies, Carlsbad, CA, USA) and maintained at 37 °C in a humidified 5% CO_2_ atmosphere. THP-1, 4T1, and Colon26 cells were cultured in RPMI1640 medium supplemented with 0.5% penicillin-streptomycin and 10% FBS under a 5% CO_2_ environment at 37 °C. ID8 cells were cultured in high-glucose DMEM supplemented with 0.5% penicillin-streptomycin, 5 µg/mL insulin (Thermo Fisher Scientific, Middlesex, MA, USA), and 5% FBS under a 5% CO_2_ environment at 37 °C.

Twenty-four hours before MCS, RAW264.7 cells were seeded in 96-well plates (5.0 × 10^4^ cells/well). Following a protocol similar to that in a previous study on THP-1 differentiation into macrophage-like cells 38, THP-1 cells, seeded 1.5 × 10^5^ cells/well, were treated with phorbol 12-myristate 13-acetate (PMA; 5 ng/mL) for 48 h before MCS.

MCS-treated RAW264.7 and PMA-treated THP-1 cells were exposed to 300 µA, 400 Hz bidirectional pulsed MCS for 15 min using ES-530 (Ito Co., Ltd., Saitama, Japan) via platinum electrodes (Gold Shousha Co., Ltd., Fukuoka, Japan), as described previously 31. The connection method and detailed current conditions are illustrated in **Figures 1A** and **S1**. Control cells were subjected to the same electrode placement and device connection but were not stimulated.

The reagents added to the cultured cells were as follows: red fluorescent labeling of RAW264.7 (Figure 1D); CellTracker Deep Red Dye (5 μM for 2 h; Thermo Fisher Scientific), inhibition of actin polymerization in RAW264.7 and THP-1 (Figure 2H,I); Cytochalasin D (2 μM for 30 m; Sigma Aldrich), activation of NR1D1 (Figure 4B,F); SR9009 (100 nM for 24 h; Sigma Aldrich), inhibition of Rho activity in cultured mouse macrophages; Rho-inhibitor I, G-switch (1 μg/mL for 4 h; Cytoskeleton, Inc., Denver, CO, USA). To synchronize the cellular circadian clock, RAW264.7 cells and THP-1 cells were treated with 100 nmol/L dexamethasone (DEX, FUJIFILM Wako Pure Chemical Corporation, Osaka, Japan) for 2 h, as previously described 28.

For mouse *Per1,* human *PER1,* mouse *Klf4*, and mouse *Calr* expression knockdown, *Per1* small hairpin RNA (shRNA) (m) Lentiviral Particles (Santa Cruz Biotechnology, Inc., TX, USA), PER1 small hairpin RNA (shRNA) (h) Lentiviral Particles (Santa Cruz Biotechnology, Inc.), Klf4 small hairpin RNA (shRNA) (m) Lentiviral Particles (Santa Cruz Biotechnology, Inc.), and Calregulin small hairpin RNA (shRNA) (m) Lentiviral Particles (Santa Cruz Biotechnology, Inc.), were transduced into RAW264.7 cells, THP-1 cells, mouse intraperitoneal macrophages, or 4T1 cells, respectively. The knockout of the *Arntl* genes in RAW264.7 and the *ARNTL* genes in THP-1 were performed using Bmal1 CRISPR/CRISPR-associated protein 9 (Santa Cruz Biotechnology, Inc), Bmal1 homology-directed repair plasmids (Santa Cruz Biotechnology, Inc), BMAL1 CRISPR/CRISPR-associated protein 9 (Santa Cruz Biotechnology, Inc), and BMAL homology-directed repair plasmids (Santa Cruz Biotechnology, Inc), as previously described 76.

Green fluorescent protein (GFP)-expressing cancer cells were constructed by transducing GFP-expressing lentivirus particles, prepared using the Lentiviral High Titer Packaging Mix with pLVSIN series (Clonetech, Palo Alto, CA), into each cancer cell line, as previously described 77. The short tandem repeat (STR) of all GFP-positive cell lines matched the ATCC and Japanese Collection of Research Bioresources Cell Bank (JCRB) database. All STR analyses are shown in **Figure S17**.

### Animal housing and treatment procedures

All animal procedures adhered to the ARRIVE guidelines and Guidelines for Animal Experiments of Kyushu University and received approval from the Institutional Animal Care and Use Committee of Kyushu University (protocol ID #A23-363-0). Five-week-old male and female BALB/c mice and C57BL/6J mice male and female were purchased from Jackson Laboratory Japan (Kanagawa, Japan). The mice were housed in a controlled environment with a temperature of 24 ± 1 °C, relative humidity of 60% ± 10%, and *ad libitum* access to standard pelleted diet and water. MCS procedures were conducted as previously described 31. Briefly, 6-7-week-old mice were anesthetized with 1.5% isoflurane (Pfizer Inc., NY, USA) by inhalation, and electrode pads (Accelgard; PALS 879100, Φ32 mm × t1.3 mm; Access Health, VIC, USA) were applied to their abdomen and back. MCS-treated mice received 300 µA, 400 Hz bidirectional pulsed MCS for 15 min via a pad affixed from ES-530 (Ito Co., Ltd.) (**Figure 2A**). Control mice underwent the same procedure but did not receive stimulation. To assess the role of macrophages in clearing cancer cells in the peritoneum and inhibiting tumorigenesis, BALB/c or C57BL/6J mice were injected with GFP-expressing 4T1 cells (5.0 × 10^5^), Hepa1-6 cells (5.0 × 10^6^), or ID8 cells (5.0 × 10^6^) via intraperitoneal or tail vein. The body weight of mice is shown in **Figure S18.** The following procedure was used to transfer *Per1*-downregulated macrophages: Intraperitoneal macrophages were removed by administering clodronate liposome (Macrokiller V300, Cosmo Bio Co., Ltd., Tokyo, Japan; 25 mg/kg, i.p.) to BALB/c mice. Macrophages collected from the abdominal cavity of another healthy BALB/c mice were infected with Per1 shRNA (m) Lentiviral Particles (Santa Cruz Biotechnology, Inc.) to downregulate *Per1* expression in the macrophages. The *Per1*-downregulated macrophages were injected into the abdominal cavity of the clodronate-treated mice 48 h following clodronate liposome administration. The mice were used in the experiment 24 h after injection of macrophages. The lungs of mice were removed 18 days post-injection of 4T1 via the tail vein, rinsed, and fixed in Bouin's solution (FUJIFILM Wako Pure Chemical Corporation) to stain the tumor nodules. To isolate metastatic tumor colonies in bone marrow, bone marrow cells were collected from mouse femora and treated with RBC lysis buffer (BioLegend, CA, USA). Cells were suspended in RPMI medium and cultured under a 5% CO_2_ environment at 37 °C for 2 weeks. 60 µM of 6-thioguanine was added to the culture medium to select 4T1 metastatic tumor cells. Tumor colonies were stained using Cell Counting Kit-8 (FUJIFILM Wako Pure Chemical Corporation) for quantification.

### Flow cytometry

RAW264.7, THP-1, mouse macrophages, and tumor cells were prepared and analyzed following established protocols 23,28,77-79.

#### Preparation of tumor-derived cells

Eight days post-transplantation, tumors around the small intestine were harvested after perfusion with 10 mL of phosphate-buffered saline (PBS) from the left ventricle. The excised tumors were digested in PBS containing 500 μg/mL of collagenase type II (FUJIFILM Wako Pure Chemical Corporation), 200 μg/mL of CaCl_2_ (Nacalai Tesque, Kyoto, Japan), 0.05% trypsin (Sigma Aldrich), and 10% FBS at 37 °C for 10 min with agitation. Subsequently, Red Blood Lysis buffer (BioLegend) was used to treat the isolated cells, followed by filtration through a 40 μm strainer.

#### Collection of free cells in the abdominal cavity

We injected 5 mL of PBS into the mouse abdominal cavity, and the intraperitoneal fluid was aspirated using a 23-gauge needle and syringe. After treatment with Red Blood Lysis buffer (BioLegend), the collected cells were filtered through a 40 μm strainer.

#### Population analysis

Following treatment with mouse TruStain FcX (BL-101320, BioLegend), the cells were stained with various antibodies: anti-mouse F4/80-APC fire (BL-123116, BioLegend), anti-mouse/human CD11b-PE (BL-101208, BioLegend), anti-mouse/human CD11b-APC (BL-101212, BioLegend), anti-mouse CD206-PE (BL-141705, BioLegend), anti-mouse CD11c-APC fire (BL-117352, BioLegend), anti-mouse SIRPα-APC fire (BL-144029, BioLegend), anti-mouse Ly6G-fluorescein isothiocyanate (FITC; BL-127606, BioLegend), anti-mouse CD3-PE (BL-100206, BioLegend), anti-mouse CD4-APC (BL-100412, BioLegend), and/or anti-mouse CD19- APC fire (BL-115558, BioLegend). Dead cells were identified using eFluor 780 viability dye (BD-565388, BD Biosciences, Erembodegem, Belgium). Flow cytometry analysis was performed using Aria III (BD Biosciences), and data were analyzed using FlowJo v10.9 (BD Biosciences). The gating strategy is illustrated in **Figure S19**.

### Assessment of phagocytic activity

Phagocytosis assays employing opsonized microbeads were conducted based on established protocols 40,80. RAW264.7, PMA-treated THP-1 cells, or peritoneal macrophages seeded in 96-well plates were exposed to either Fluoresbrite YG Microspheres 1.00 µm (Polysciences Inc., PA, USA) or GFP-expressing cancer cells (1.0 × 10^5^ cells). After 3 h, cells were washed with PBS, and GFP fluorescence was quantified using fluorescence-activated cell sorting. The gating strategy is illustrated in **Figure S19**.

### RNA sequencing (RNA-seq)

Peritoneal macrophages (CD11b^+^ F4/80^+^ Ly6G^-^ cells) were isolated from 6-week-old female BALB/c mice via flow cytometry. Total RNA was extracted from the cells using the ReliaPrep RNA Miniprep Systems (Promega, Madison, WI, USA). For RNA sequencing, the sequencing libraries were prepared from 200 ng of total RNA with MGIEasy rRNA Depletion Kit and MGIEasy RNA Directional Library Prep Set (MGI Tech Co., Ltd., Shenzhen, China) according to the manufacturer's instructions. The libraries were sequenced on the DNBSEQ-G400 FAST Sequencer (MGI Tech Co., Ltd.) with paired-end 150 nt strategy. For sequencing data analysis, all sequencing reads were trimmed of low-quality bases and adapters with Trimmomatic (v.0.38) 81. Raw counts for each gene were estimated in each sample using RSEM version 1.3.0 and Bowtie 2 82,83. To detect the differentially expressed genes, we used edgeR 84 program. Normalized counts per million (CPM) values and log fold-changes (logFC) were obtained from the gene-level raw counts. Raw RNA-seq data were deposited in Gene Expression Omnibus at NCBI (GSE265902). Genes shown in Figures 2D and 3A were selected based on the following criteria: |logFC| > 1 and logCPM > 0 or |logFC| > 3 and logCPM > 0, respectively. Functional analysis of upregulated genes was conducted using the Kyoto Encyclopedia of Genes and Genomes (KEGG) database on the DAVID system and Gene Ontology (GO) analysis. Gene lists corresponding to **Figures 2D**, **3A**, and **5E**,**F** are provided in **Tables** S**1**-S**4**.

### Quantitative Reverse Transcription Polymerase Chain Reaction (RT-PCR)

Total RNA was isolated using the ReliaPrep RNA Miniprep Systems (Promega). cDNA synthesis was performed using the ReverTra Ace qPCR RT kit (Toyobo, Osaka, Japan), followed by PCR amplification. RT-PCR analysis was performed on diluted cDNA samples utilizing the THUNDERBIRD SYBR qPCR Mix (Toyobo) with the 7500 Real-time PCR system (Applied Biosystems, Foster City, CA, USA). Data normalization was conducted using 18S and β-actin mRNA as internal controls. Primer sequences are provided in **Table S5**.

### Immunofluorescence histochemical staining

Following fixation of cells in PBS containing 4% paraformaldehyde, membrane permeabilization was achieved by incubation in PBS containing 0.1% TritonX-100 (0.1% Triton-PBS) for 10 min. Subsequently, the cells were blocked with 0.1% Triton-PBS containing 2% bovine serum albumin and incubated with rat anti-mouse F4/80 antibody (Bio-Rad Laboratories, Inc., Hercules, CA, USA) for 24 h. After washing the cells with 0.1% Triton-PBS, Alexa Fluor 546-conjugated goat anti-rat IgG antibody (Life Technologies) was applied and allowed to react for 2 h. Cells designated for F-actin staining were treated with Acti-stain 488 phalloidin (Cytoskeleton, Inc.) solution for 30 min at 4 °C. Following PBS washes, samples were mounted using VECTASHIELD Mounting Medium with 4′,6-diamidino-2-phenylindole (DAPI; Vector Laboratories, NY, USA). Imaging and analysis were conducted using LSM700 (Zeiss, Oberkochen, Germany) and BZ-9000 (KEYENCE, Osaka, Japan) systems.

### Western blotting

To collect total protein, RAW264.7, 4T1, THP-1, and mice intraperitoneal macrophages were homogenized in CelLytic Cell Lysis Reagent (Sigma Aldrich). Active-RhoA protein in mouse macrophage and RAW264.7 cells were collected using the RhoA Activation Assay Biochem Kit (Cytoskeleton), performed according to the manufacturer's instructions. Subsequently, samples were separated using sodium dodecyl sulfate-polyacrylamide gel electrophoresis, and the proteins were then transferred to polyvinylidene difluoride membranes (Immobilon-P; Merck Millipore, MA, USA). Primary antibodies against ARNTL (1:1,000; ab235577, Abcam, Cambridge, MA, USA), CALR (1:1,000; ab92516, Abcam), KLF4 (1:1,000; #4038; Cell Signaling Technology, Inc., Danvers, MA, USA), PER1 (1:1,000; PM091; MEDICAL & BIOLOGICAL LABORATORIES CO., LTD., Tokyo, Japan), CREB (1:1,000; #4820; Cell Signaling Technology, Inc.), phospho-CREB (1:1,000; #9198; Cell Signaling Technology, Inc.), and ACTB (SC-47778, Santa Cruz Biotechnology), which were diluted using Can Get Signal Immunoreaction Enhancer Solution (Toyobo), were used to incubate the membranes. The immunocomplexes were reacted with anti-guinea pig, or anti-rabbit IgG secondary antibody and then with Chemi-Lumi One reagent (Nacalai Tesque, Inc.). Next, the membranes were imaged, and the density of each band was analyzed using an ImageQuant LAS 3000 mini (Fuji Film, Co., Ltd). All uncropped images are presented in the corresponding Figures in Supplementary Material.

### Statistical analysis

Statistical analyses were performed using the JMP Pro 17 software (SAS Institute Japan, Tokyo, Japan). Differences among multiple groups were assessed using two-way or one-way analysis of variance with Tukey-Kramer's post-hoc test, whereas the two-sided unpaired *t*-test was utilized for comparisons between pairs of groups. Statistical significance was defined as *P* ≤ 0.05 and *P* < 0.01.

## Conclusions

In this study, we elucidated a mechanism whereby MCS mitigates cancer cell engraftment and tumorigenesis in the abdominal cavity by enhancing macrophage phagocytic activity via clock genes. This mechanism may underlie the involvement of the circadian clock in resistance to treatment with ICIs, as evidenced by numerous animal and clinical studies 27,28,85,86. Moreover, this mechanism, which can counteract the time-dependent decline in macrophage phagocytosis, holds promise for overcoming therapeutic resistance. Furthermore, given the significant immune-related adverse events associated with existing cancer immunotherapies targeting self-nonself recognition signals, such as the PD1-PDL1 interaction and SIRPα-CD47 87,88, the development of therapies centered on MCS and clock genes may offer a solution to these challenges in cancer immunotherapy.

## Supplementary Material

Supplementary figures and tables.

## Abbreviations

CPM: counts per million
Cry: cryptochrome
DAPI: 4′,6-diamidino-2-phenylindole
DEX: dexamethasone
FBS: fetal bovine serum
FITC: fluorescein isothiocyanate
GO: Gene Ontology
GFP: green fluorescent protein
ICIs: immune checkpoint inhibitors
KEGG: Kyoto Encyclopedia of Genes and Genomes
KO: knockout
logFC: log fold-change
MEFs: mouse embryonic fibroblasts
MCS: microcurrent stimulation
Per: period
PMA: phorbol 12-myristate 13-acetate
RNA-seq: RNA sequencing
shRNA: small hairpin RNA
TAMs: tumor-associated macrophages
ZT: Zeitgeber Time
