# Supplementary material for: Targeting macrophage circadian rhythms with microcurrent stimulation to activate cancer immunity through phagocytic defense
Source: Theranostics. 2025 Jan 1;15(2):340–61. doi: 10.7150/thno.100748 (PMC11671381; doi:10.7150/thno.100748)
Supplement: Supplementary file 1 — Supplementary figures and tables. [file thnov15p0340s1.pdf]

Supplementary Material

Targeting macrophage circadian rhythms with microcurrent stimulation to activate cancer immunity through phagocytic defense

Yuya Yoshida*, Tomohito Tanihara, Keika Hamasaki, Fumiaki Tsurusaki, Taiki Fukuda, Satoka Adachi, Yuma Terada, Kaita Otsuki, Naoki Nishikawa, Kohei Fukuoka, Ryotaro Tsukamoto, Kengo Hamamura, Kosuke Oyama, Akito Tsuruta, Kouta Mayanagi, Satoru Koyanagi, Shigehiro Ohdo*, and Naoya Matsunaga*

***Corresponding authors:**

Yuya Yoshida

Department of Clinical Pharmacokinetics, Faculty of Pharmaceutical Sciences, Kyushu University, 3-1-1 Maidashi Higashi-ku, Fukuoka 812-8582, Japan

Tel.: +81 92-642-6658

Email: yoshida@phar.kyushu-u.ac.jp

Naoya Matsunaga

Department of Clinical Pharmacokinetics, Faculty of Pharmaceutical Sciences, Kyushu University, 3-1-1 Maidashi Higashi-ku, Fukuoka 812-8582, Japan

Tel. : +81 92-642-6656

Email: matunaga@phar.kyushu-u.ac.jp

Shigehiro Ohdo

Department of Clinical Pharmacokinetics, Faculty of Pharmaceutical Sciences, Kyushu University, 3-1-1 Maidashi Higashi-ku, Fukuoka 812-8582, Japan

Tel.: +81 92-642-6610

Email: ohdo@phar.kyushu-u.ac.jp

This file includes:

Figures S1 to S19

Tables S1 to S5

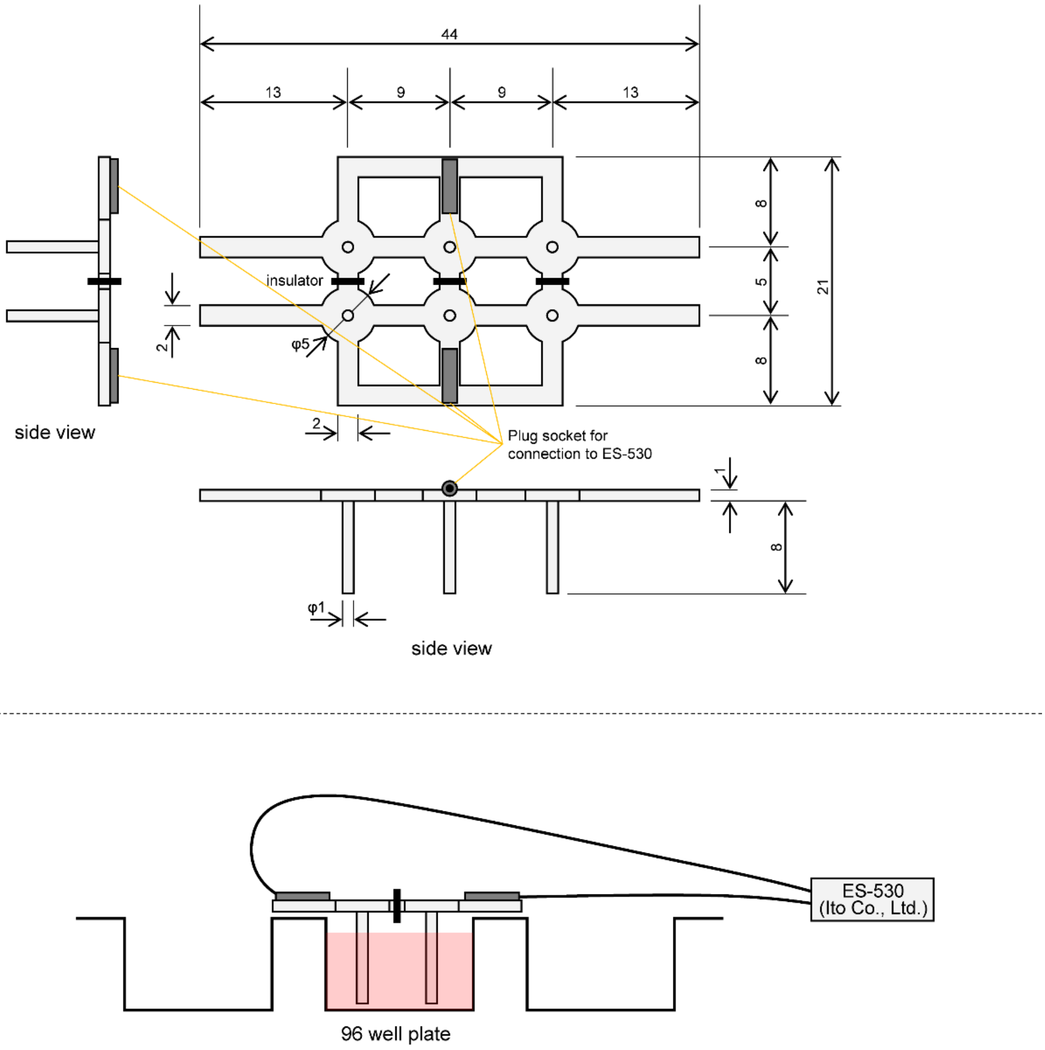

Figure S1. Design of platinum electrodes used for microcurrent stimulation (MCS) in RAW264.7 and PMA-treated THP-1 cells.

All parts were fabricated with platinum except for the insulator part. Platinum with a purity of at least 99.95% was used in the fabrication of the electrodes. MCS: microcurrent stimulation.

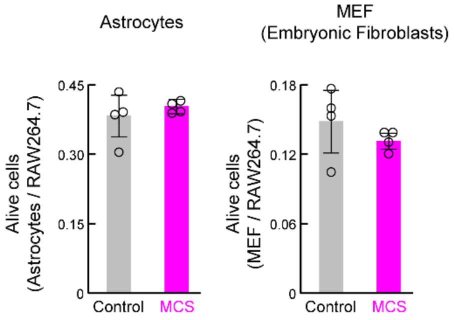

Figure S2. The number of living mouse embryonic fibroblasts (MEF) and mouse astrocytes after co-cultured with RAW264.7 cells. The number of living cells was determined immediately after the addition of MEF and astrocytes 12 h after microcurrent stimulation (MCS) for RAW264.7 cells, and co-incubated for another 3 h. Data are presented as the mean ± S.D. (n = 4).

Term (GO molecular function)	%	P-Value
protein binding	26.2	<0.01
identical protein binding	11.6	<0.01
RNA polymerase II core promoter proximal region sequence-specific DNA binding	5.9	0.043
calcium ion binding	4.3	0.011
transcription factor activity, sequence-specific DNA binding	3.9	0.014
transcriptional activator activity, RNA polymerase II transcription regulatory region sequence-specific binding	3.6	<0.01
receptor binding	3.5	<0.01
sequence-specific DNA binding	3.1	0.014
peptidase activity	3	0.043
actin binding	2.6	0.019
transmembrane signaling receptor activity	1.9	<0.01
serine-type endopeptidase activity	1.8	<0.01
microtubule binding	1.8	0.037
transcription regulatory region sequence-specific DNA binding	1.8	0.044
cytokine activity	1.7	0.026
extracellular matrix structural constituent	1.5	<0.01
integrin binding	1.5	<0.01
serine-type peptidase activity	1.5	<0.01
heme binding	1.5	0.021
lyase activity	1.4	0.014
G-protein coupled receptor binding	1.2	<0.01
protein tyrosine kinase activity	1.1	0.045
collagen binding	1	<0.01
transmembrane receptor protein tyrosine kinase activity	0.8	0.011
cadherin binding	0.8	0.044
oxygen binding	0.6	<0.01
extracellular matrix structural constituent conferring tensile strength	0.6	0.023
ionotropic glutamate receptor binding	0.6	0.034
alpha-tubulin binding	0.6	0.034
microtubule motor activity	0.6	0.042
RNA polymerase II transcription factor activity, ligand-activated sequence-specific DNA binding	0.6	0.042
haptoglobin binding	0.5	<0.01
oxygen transporter activity	0.5	<0.01
hydro-lyase activity	0.5	<0.01
tumor necrosis factor receptor binding	0.5	0.034
calcium-activated potassium channel activity	0.4	<0.01
platelet-derived growth factor binding	0.4	<0.01
organic acid binding	0.4	0.01
carbonate dehydratase activity	0.4	0.022
inward rectifier potassium channel activity	0.4	0.049
hemoglobin beta binding	0.3	0.012
kinetochore binding	0.3	0.018
hemoglobin alpha binding	0.3	0.032
hyaluronoglucosaminidase activity	0.3	0.04
hemoglobin binding	0.3	0.049

Figure S3. Gene Ontology analysis of genes exhibiting MCS-dependent expression variation, based on RNA-seq results obtained from RNA extracted from intraperitoneal macrophages collected 12 h post-MCS.

All terms with $P < 0.05$ are shown. The hierarchy of parent-child terms targeting the top 25 match rates is shown in **Figure 2D**. The analysis includes genes with a Control-to-MCS ratio > 2. The gene list is provided in **Table S1**.

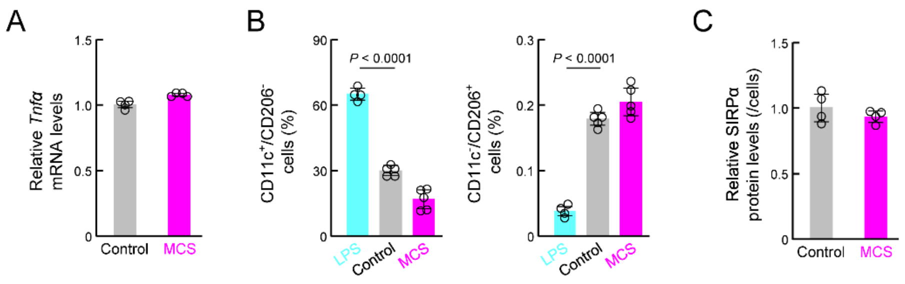

Figure S4. Influence of microcurrent stimulation (MCS) on cytokine, M1/M2 differentiation, and SIRPα expression.

(**A**) mRNA expression levels of *Tnfa* in control and MCS-treated RAW264.7 cells. (**B**) Left: variation in the ratio of $CD11c^{+}$ $CD206^{-}$ RAW264.7 cells among all cells. Right: Variation in the ratio of $CD11c^{-}$ $CD206^{+}$ RAW264.7 cells among all cells. (**C**) SIRPα expression of RAW264.7 per cell assessed using flow cytometry. The control value is normalized to 1.0. All values were measured 12 h after MCS. Data are expressed as the mean ± S.D. (n = 4).

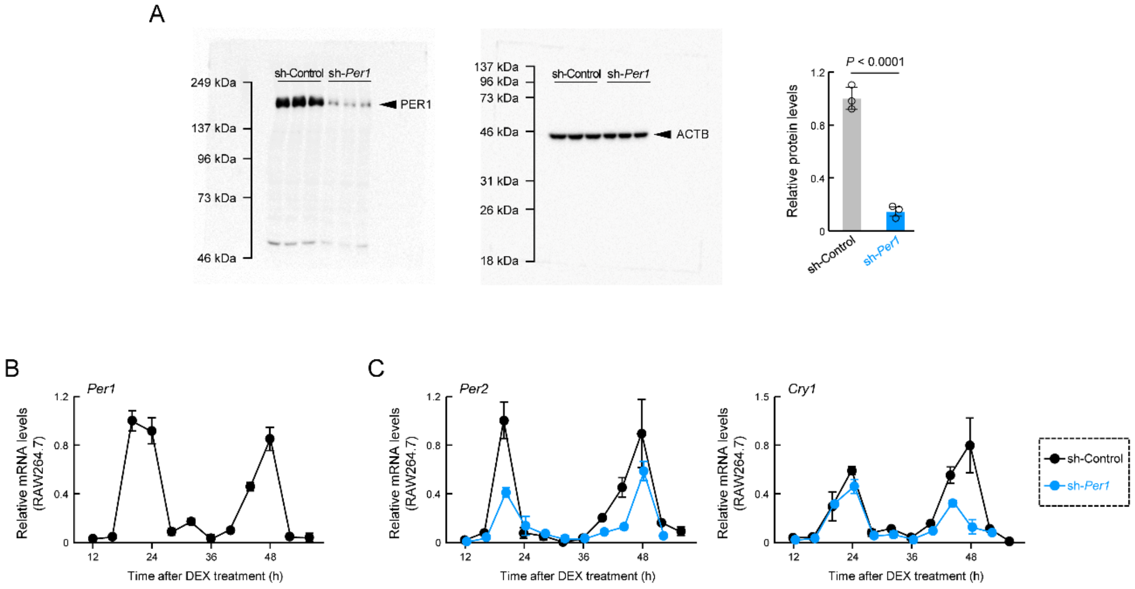

Figure S5. Effect of dexamethasone and sh-*Per1* on the circadian rhythm of clock genes expression in RAW264.7.

(**A**) Protein expression of PER1 and ACTB in RAW264.7 transduced sh-Control or sh-*Per1* lentivirus used in **Figures 3G**, **4D, and S14E**. Left panels indicate uncropped western blot images. (**B**) Temporal expression profiles of *Per1* mRNA in RAW264.7 cells following synchronization of the circadian clock. Cells were treated with 100 nM dexamethasone for 2 h to synchronize the circadian clock, and the mRNA levels of *Per1* were assessed at the indicated time points. A significant time-dependent variation in the mRNA levels of *Per1* ($P < 0.05$; one-way ANOVA) was observed. (**C**) Temporal expression profiles of *Per2* and *Cry1* mRNA in RAW264.7 cells with sh-Control and sh-*Per1* following synchronization of the circadian clock. Cells were treated with 100 nM dexamethasone for 2 h to synchronize the circadian clock, and the mRNA levels of *Per2* and *Cry1* were assessed at the indicated time points. A significant time-dependent variation in the mRNA levels of *Per2* and *Cry1* ($P < 0.05$; one-way ANOVA) was observed. Data are expressed as the mean with S.D. (n = 4). sh-*Per1*, *Per1* small hairpin RNA.

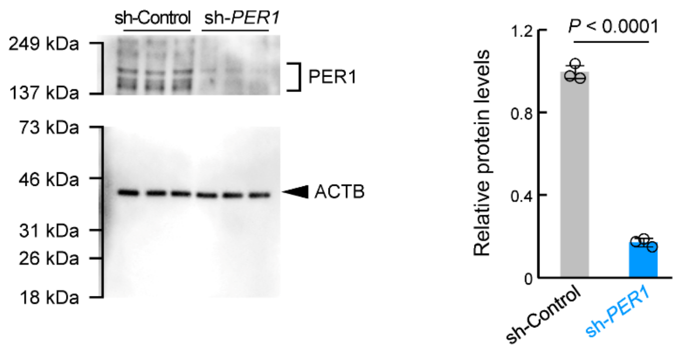

Figure S6. Effect of sh-*PER1* on THP-1.

Protein expression of PER1 and ACTB in THP-1 transduced sh-Control or sh-*PER1* lentivirus used in **Figure 3H**. Left panels indicate uncropped western blot images. sh-*PER1*, *PER1* small hairpin RNA.

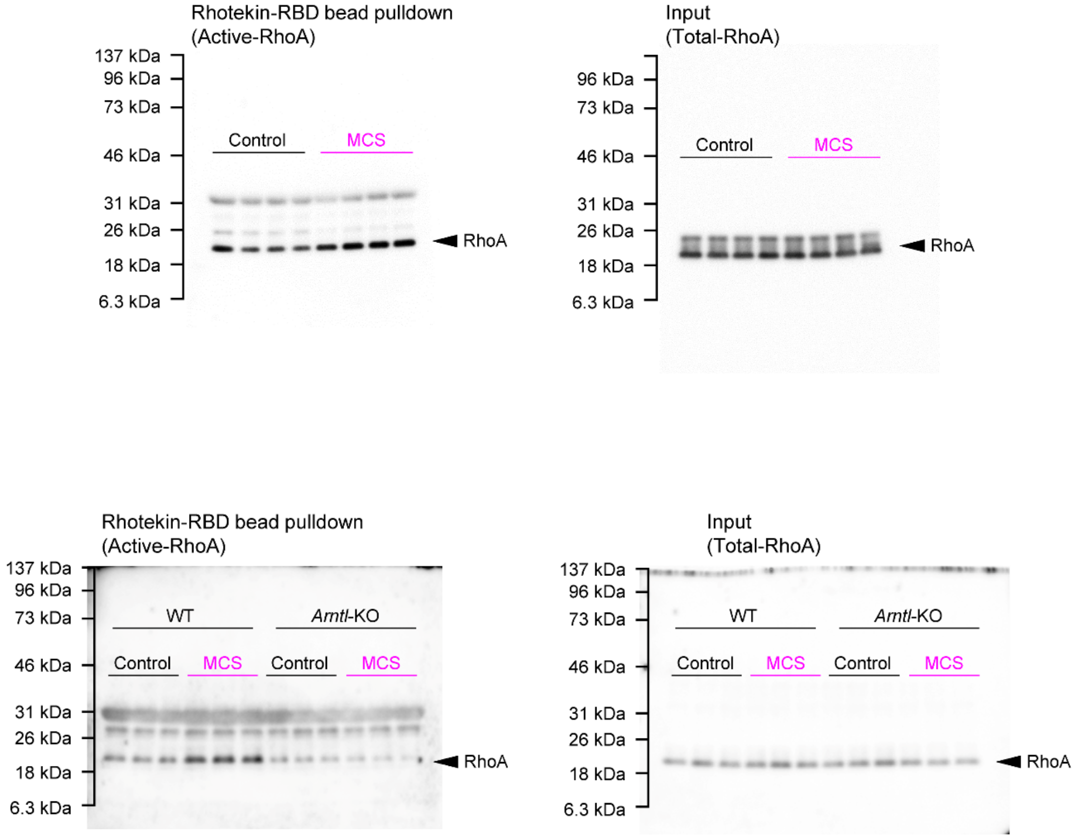

Figure S7. Uncropped western blot images using Figure 5.

A

ChIP-Atlas, P <0.01 and Fold Enrichment >4

ID	Antigen	Log P-value	Log Q-value	Fold Enrichment
SRX1532691	E2f4	-11.8	-7.8	6.58
SRX1532694	E2f4	-10.6	-6.7	6.47
SRX1532692	E2f4	-10.2	-6.5	6.16
SRX122394	E2f4	-14.3	-9.9	11.45
SRX122397	E2f4	-7.8	-4.3	7.72
SRX344966	Epitope tags	-4.5	-1.4	4.18
SRX1120754	E2f3	-4.1	-1.1	4.49
SRX122396	E2f4	-4	-1	6.67
SRX118005	Gata1	-3.1	-0.4	4.75
SRX1120752	E2f1	-2.8	-0.3	4.93
SRX8713937	Klf4	-2.5	-0.1	4.31
SRX1547317	Srf	-2.5	-0.1	4.15
SRX495627	Rorc	-3	-0.4	6.84
SRX1035395	Gfi1	-2.8	-0.3	6.12
SRX13554160	Mef2c	-2.6	-0.1	5.43
SRX1979970	Neurod2	-2.6	-0.1	5.36
SRX10157960	Klf5	-2.4	-0.1	4.96
SRX5358289	Pax3	-2.3	-0.1	4.47
SRX22006953	Nfyc	-2.2	-0.1	4.34

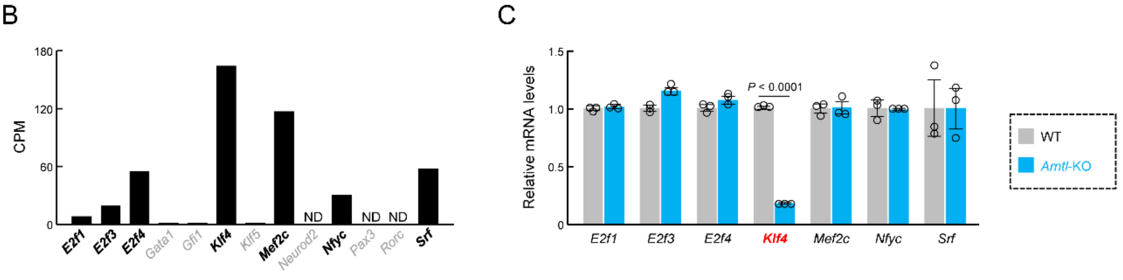

Figure S8. Screening for a transcription factor (TF) that mediates the effects of MCS on genes whose expression levels are time dependent.

(**A**) ChIP-Atlas enrichment analysis using genes whose expression differed between ZT2 and ZT14 in control mice and increased by MCS in ZT14. The threshold values were set at $P < 0.01$ and Fold Enrichment > 4. Gene lists are shown in **Table S4**. (**B**) Comparison of mRNA expression levels of candidate transcription factors in mouse peritoneal macrophages using RNA-seq results. Genes with CPM values <1 are shown in gray; these were excluded from the candidate transcription factors. (**C**) Expression levels of candidate transcription factors in wild-type and *Arntl*-KO RAW264.7 cells. For panel C, data are expressed as the mean ± S.D. (n = 3). Statistical significance was determined using two-tailed Student's *t*-tests. *P*-values are shown in each graph. MCS: microcurrent stimulation; S.D.: standard deviation; MCS: microcurrent stimulation.

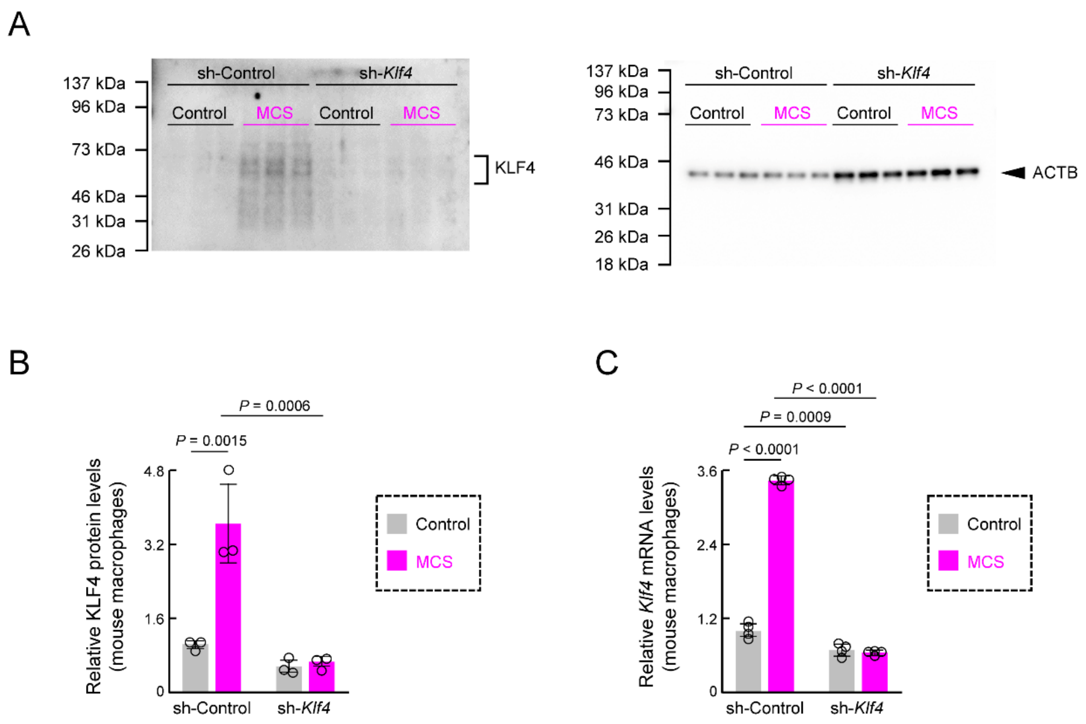

Figure S9. Effect of sh-*Klf4* and MCS on mouse peritoneal macrophages.

(**A-C**) Protein expression of KLF4 and ACTB (**A,B**) and mRNA expression of *Klf4* (**C**) in MCS-treated cultured mouse peritoneal macrophages transduced sh-Control or sh-*Klf41* lentivirus used in **Figure 5I-K**. All values were measured 12 h following MCS. Panel A shows uncropped western blot images. Data are expressed as the mean ± S.D. (n = 3-4). Statistical significance was determined using two-way ANOVA with Tukey–Kramer post-hoc tests. *P*-values are shown in each graph. ANOVA: analysis of variance; sh-*Klf4*, *Klf4* small hairpin RNA; MCS: microcurrent stimulation.

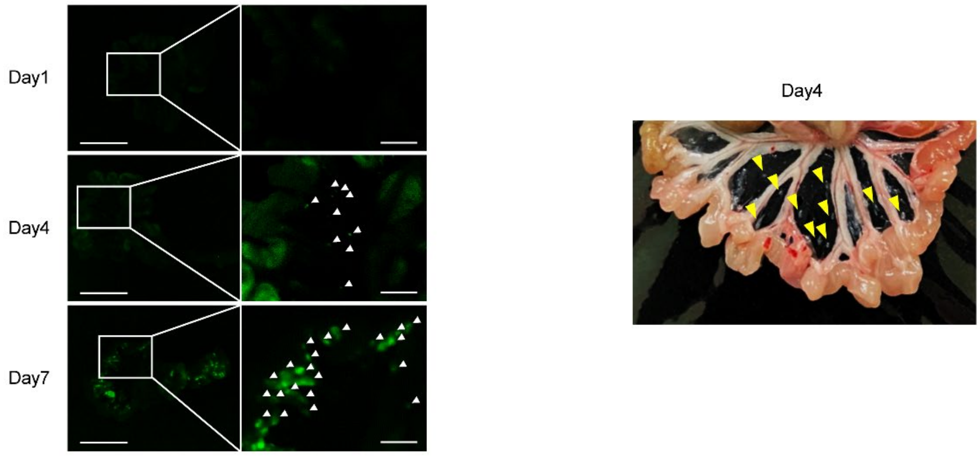

Figure S10. Tumor growth following intraperitoneal implantation of 4T1.

Female BALB/c mice were injected i.p. 5.0×10^5 GFP-expressing 4T1 cells/500 µL PBS/mouse. The left panel shows GFP fluorescence around the small intestine at 1, 4, and 7 days after transplantation; weak GFP fluorescence from the nodular tumor was observed from day 4. The right panel shows brightfield images of the small intestine and periportal area on day 4. Arrows indicate nodular tumors. GFP: green fluorescent protein; i.p.: intraperitoneal; PBS: phosphate-buffered saline.

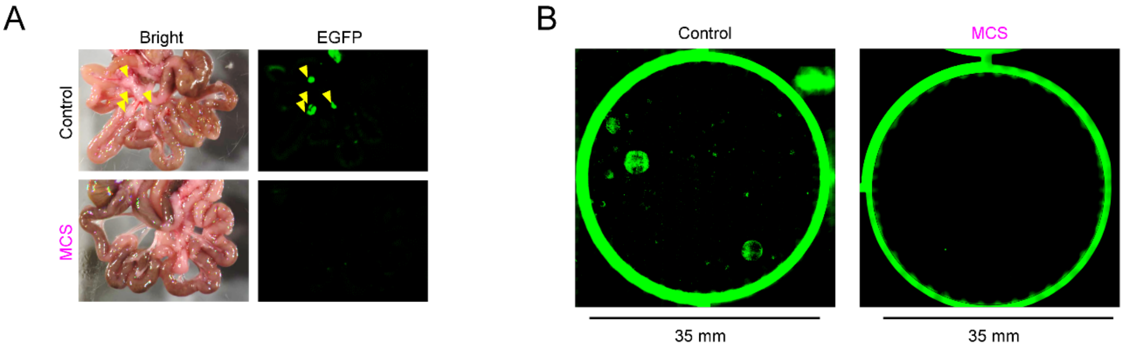

Figure S11. Effect of microcurrent stimulation on the metastatic potential of 4T1 cells.
(**A**) Photographic and GFP-fluorescence images of the removed small intestine prepared from control and MCS-treated mice injected with 4T1 via the tail vein. Yellow arrows indicate nodular tumors formed around the portal vein. The comparison of the quantified GFP-positive area is shown in **Figure 8M**. (**B**) GFP-fluorescence image of metastatic colonies isolated from tumor-bearing mice femora bone marrow. Cells were cultured in a 35 mm dish with 60 µM of 6-thioguanine. Two weeks after culture, colonies derived from metastatic 4T1 cells were stained using Cell Counting Kit-8 for quantification. The comparison of the quantified colonies area is shown in **Figure 8O**. GFP: green fluorescent protein; MCS: microcurrent stimulation.

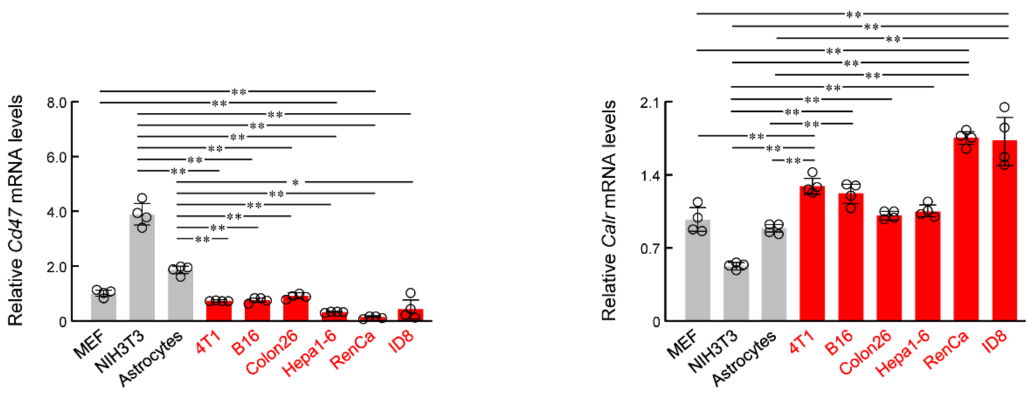

Figure S12. Expression of eat-me and don't-eat-me signaling receptors in cancer cells.

mRNA expression of *Cd47* and *Calr* in tumor-derived (4T1, B16, Hepa1-6, Colon26, RenCa, ID8) and non-tumor-derived (MEF, NIH3T3, Astrocyte) cancer cell lines in mice. Data are shown as the mean ± S.D. (n = 4). The value of 4T1 is set at 1.0. **$P < 0.01$, *$P < 0.05$; significant difference from each group (one-way ANOVA with Tukey–Kramer post-hoc test). ANOVA: analysis of variance; MEFs: mouse embryonic fibroblasts; S.D.: standard deviation.

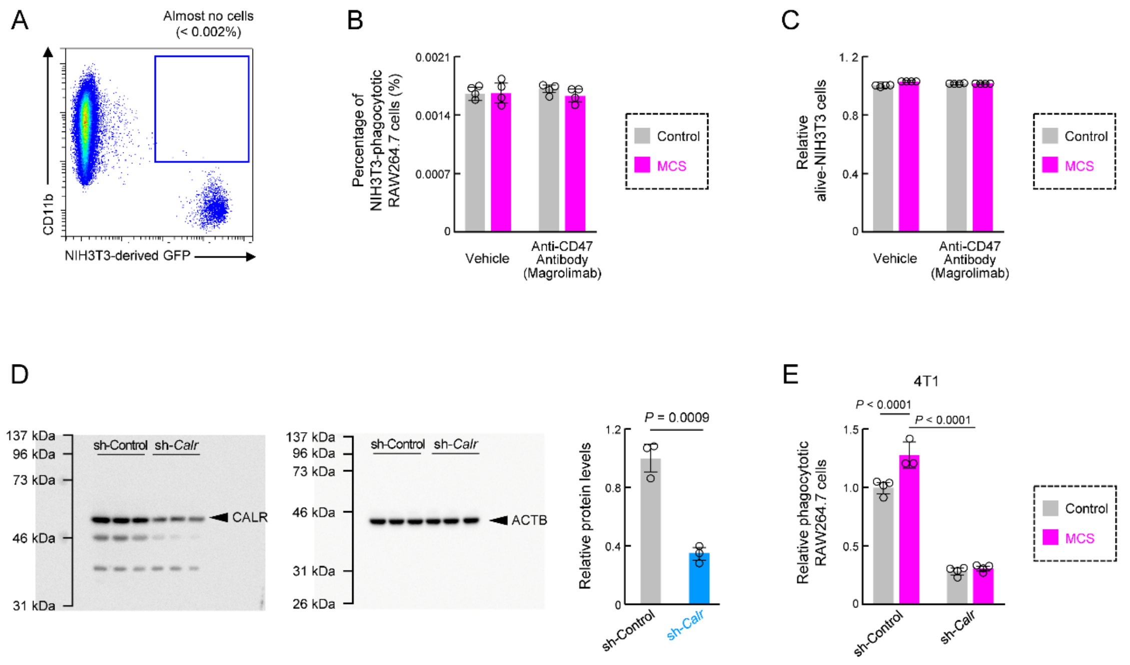

Figure S13. Effect of sh-*Calr* transduction into 4T1 on the activation of RAW264.7 phagocytosis by MCS.

(**A**) Representative flow cytometry panel of RAW264.7 cells and NIH3T3 cells co-cultured for 3 h. The CD11b^{+} cell population in the upper left is RAW264.7 cells, and the GFP^{+} cell population in the lower right is NIH3T3 cells. There were negligible CD11b^{+}/GFP^{+} cells, which are macrophages that have engulfed NIH3T3 cells (<0.002%). (**B,C**) Effects of CD47 inhibitor (magrolimab; 10 µg/mL) exposure of the phagocytic activity (B) and number of surviving NIH3T3 cells (C) measured using NIH3T3 cells of RAW264.7 cells under MCS. Magrolimab was added 11 h and 30 m post-MCS, and 30 m later of the addition of magrolimab, NIH3T3 cells was added. The phagocytic activity and number of surviving NIH3T3 cells were measured immediately 3 h after the addition of NIH3T3 cells. The percentage of RAW264.7 cells that showed phagocytic activity was 0.002% or less in all groups, and neither magrolimab nor MCS affected. (**D**) Protein expression of CALR and ACTB in GFP-4T1 cells transduced sh-Control or sh- *Calr* lentivirus. Left panels indicate uncropped western blot images. (**E**) Relative number of RAW264.7 cells phagocytosing sh-Control or sh-*Calr* transducted GFP-4T1 cells. The phagocytic activity was measured immediately after the addition of each cancer cell 12 h after MCS and co-incubated for another 3 h. Data are expressed as the mean ± S.D (n = 3-5). Statistical significance was determined two-tailed Student's *t*-tests (D) and two-way ANOVA with Tukey–Kramer post-hoc tests (B,C, and E). *P*-values are shown in each graph. ANOVA: analysis of variance; sh-*Calr*, *Calr* small hairpin RNA.

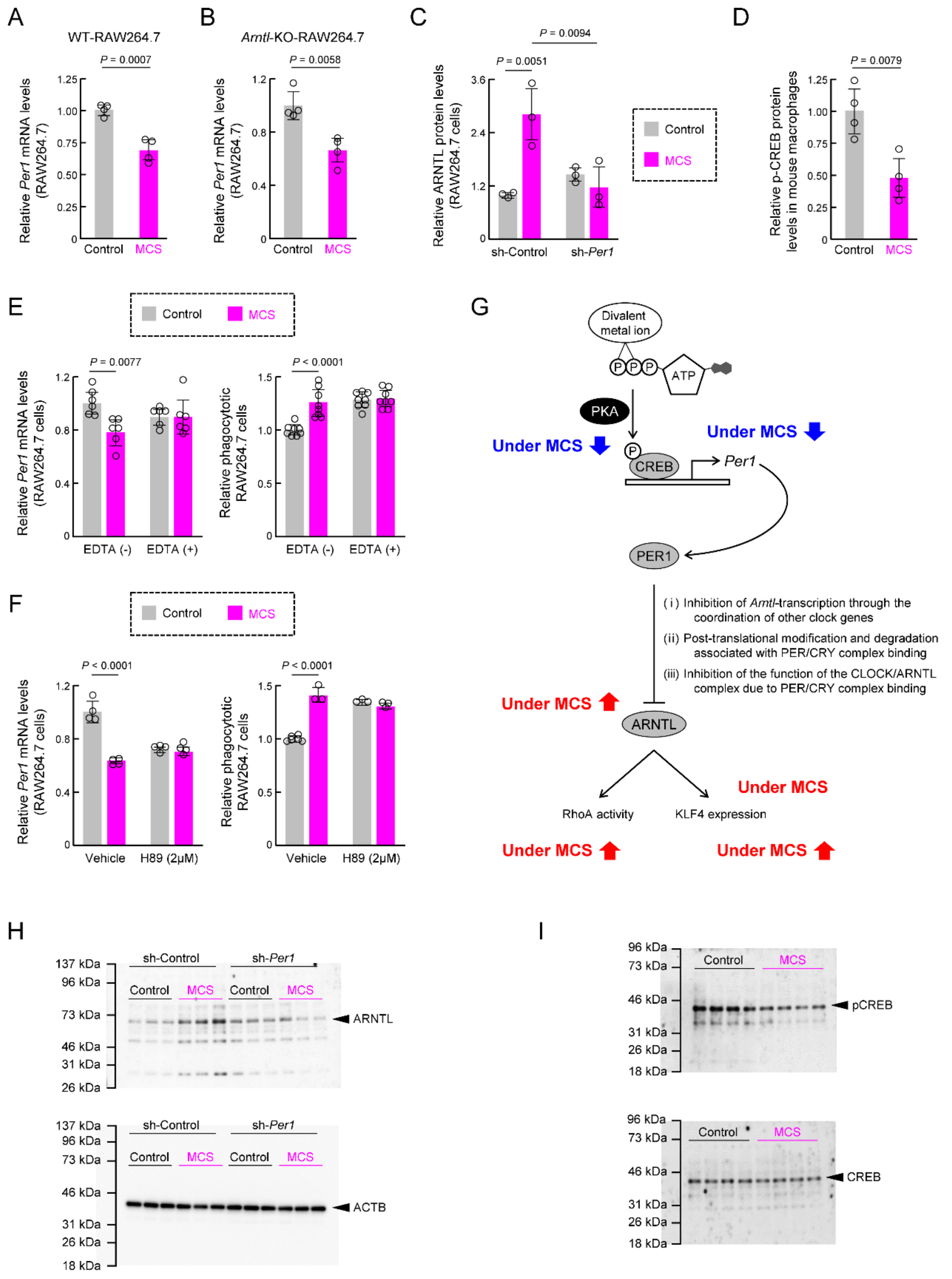

Figure S14. Mechanism underlying *Per1* downregulation by microcurrent stimulation. (**A,B**) Effect of MCS on the expression level of *Per1* mRNA in WT (**A**) and *Arntl*-KO (**B**) RAW264.7 cells. *Per1* mRNA was measured 15 m post-MCS. MCS decreased *Per1* expression in both cell lines. (**C**) Protein expression of ARNTL in RAW264.7 cells transduced sh-Control or sh-*Per1* lentivirus. The

protein levels were measured 12 h after MCS. (**D**) The expression levels of p-CREB protein in intraperitoneal macrophages from Control or MCS-treated female BALB/c mice. Macrophages were collected 15 m post-MCS. (**E**) Effects of PKA inhibitor (H89; 2μM) exposure on the expression of *Per1* mRNA (left) and phagocytic activity measured using opsonized bead (right) of RAW264.7 cells under MCS. H89 was added to the medium 30 m before MCS. *Per1* mRNA was measured 15 m post-MCS. Beads were added 12 h post-MCS and co-incubated for another 3 h. The phagocytic activity was measured immediately after the addition of beads. (**F**) Effects of EDTA (1mM) exposure on the expression of *Per1* mRNA (left) and phagocytic activity measured using opsonized bead (right) of RAW264.7 cells under MCS. EDTA was added to the medium 30 m before the start of MCS. *Per1* mRNA was measured 15 m post-MCS. Beads were added 12 h post-MCS and co-incubated for another 3 h. The phagocytic activity was measured immediately after the addition of beads. (**G**) The pathway by which MCS acts on the ARNTL protein via *Per1*. (**H,I**) Uncropped western blot images using panel C (H) and D (I). Data are expressed as the mean ± S.D (n =3-6). Statistical significance was determined using two-way ANOVA with Tukey–Kramer post-hoc tests. P-CREB: phospho-CREB; sh-*Per1*, *Per1* small hairpin; MCS: microcurrent stimulation; PKA: protein kinase A; EDTA: ethylenediaminetetraacetic acid; WT: Wild-type.

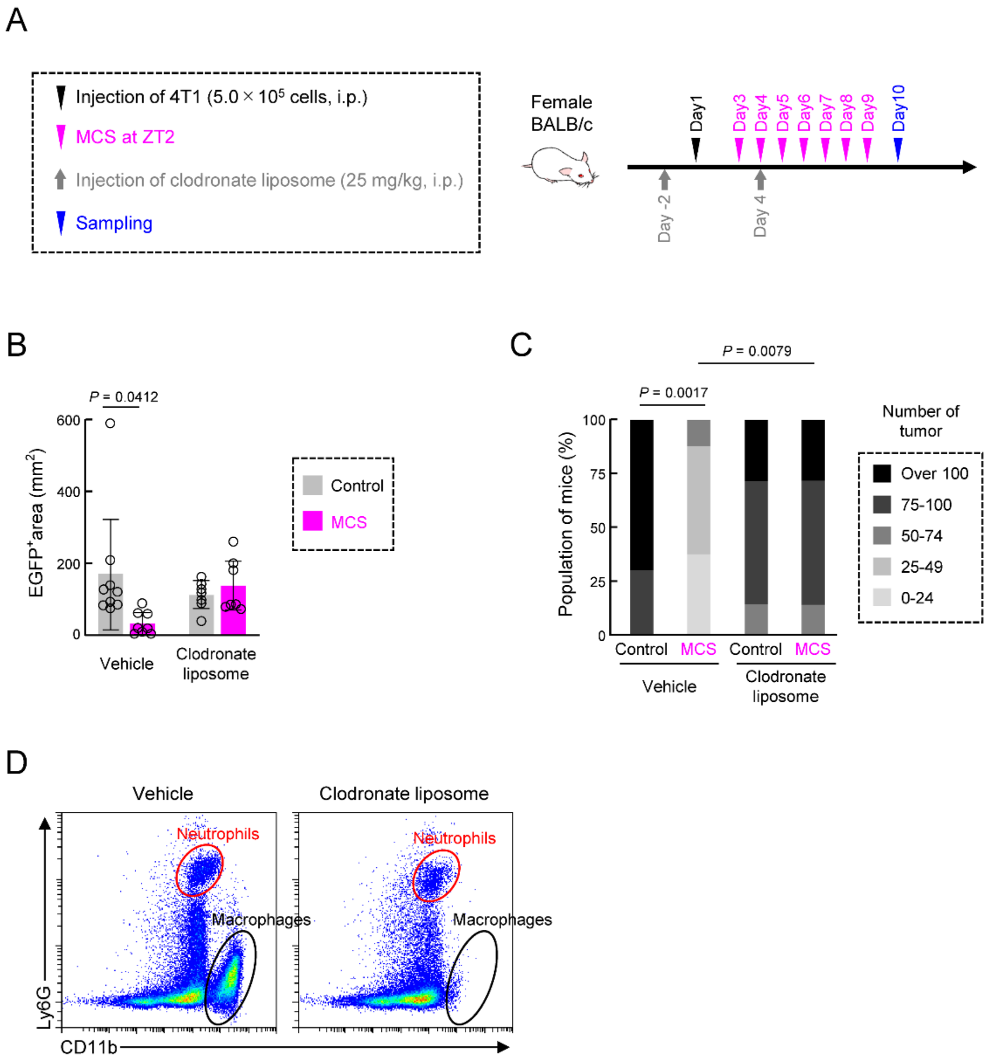

Figure S15. Effect of macrophage depletion on the antitumor effect of microcurrent stimulation. (**A**) Protocol of macrophage removal by clodronate liposome, MCS treatment, and 4T1 cell injection. (**B**) The 4T1-derived GFP-positive area around the portal vein. (**C**) The number of nodular tumors formed in the abdominal cavity. Each mouse was ranked based on tumor count; the percentage of mice in each rank is presented for each group. (**D**) Representative flow cytometry images of CD11b and Ly6G expression in mice administered and not administered clodronate liposomes. CD11b is expressed in macrophages and neutrophils, whereas Ly6G is expressed only in neutrophils. The population of CD11b-positive, Ly6G-negative cells, including macrophages, disappeared as a result of the addition of clodronate liposomes; however, the population of CD11b-positive, Ly6G-positive cells, which are neutrophils, did not disappear. Data are expressed as mean ± S.D. (n = 4–10). Statistical significance was determined using two-way ANOVA with Tukey–Kramer post-hoc tests. *P*-values are shown in each graph. ANOVA: analysis of variance; GFP: green fluorescent protein; MCS: microcurrent stimulation; S.D.: standard deviation.

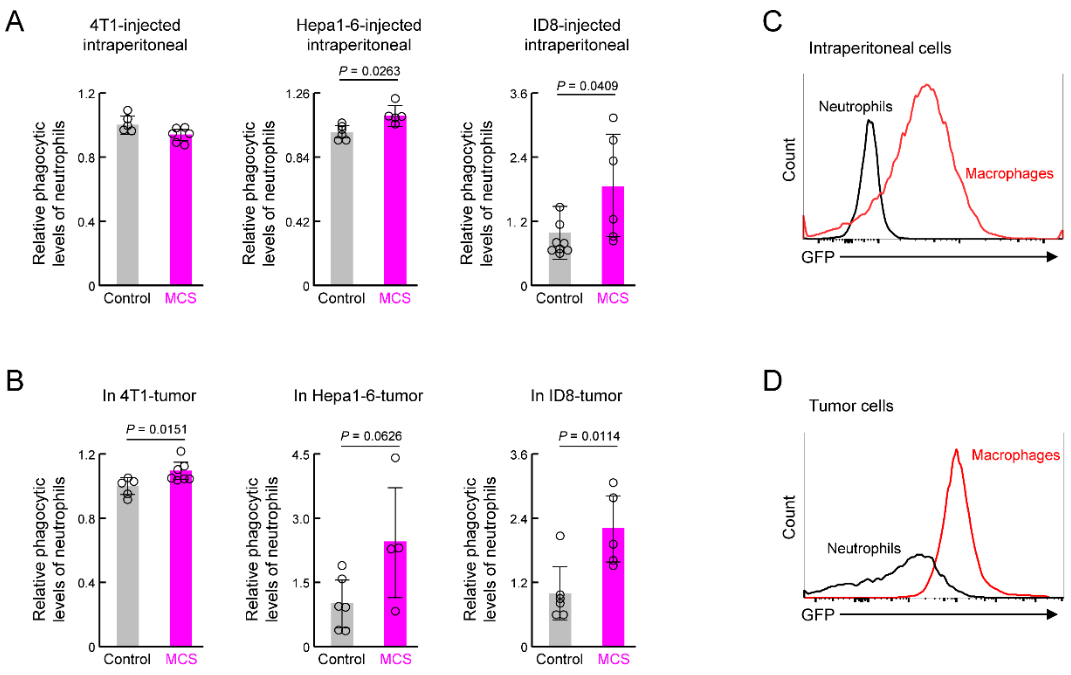

Figure S16. Phagocytic activity of neutrophils in mice transplanted with cancer cells.

(**A**) The number of GFP-positive neutrophils derived from cancer cells in the abdominal cavity of mice transplanted with 4T, Hepa1-6, and ID8. The schedule for the transplantation of cancer cells and MCS for each mouse is shown in **Figures 6A** and **7A**,**I**. (**B**) The number of GFP-positive neutrophils derived from cancer cells in the tumor of mice transplanted with 4T, Hepa1-6, and ID8. The schedule for the transplantation of cancer cells and MCS for each mouse is shown in **Figures 6G** and **7D**,**L**. (**C**,**D**) Comparison of GFP fluorescence intensity derived from 4T1 in macrophages and neutrophils prepared from the abdominal cavity and tumors of MCS-treated mice transplanted with 4T1. Data are expressed as the mean ± S.D. (n = 4–10). Statistical significance was determined using two-tailed Student's *t*-tests. *P*-values are shown in each graph. ANOVA: analysis of variance; GFP: green fluorescent protein; MCS: microcurrent stimulation; S.D.: standard deviation.

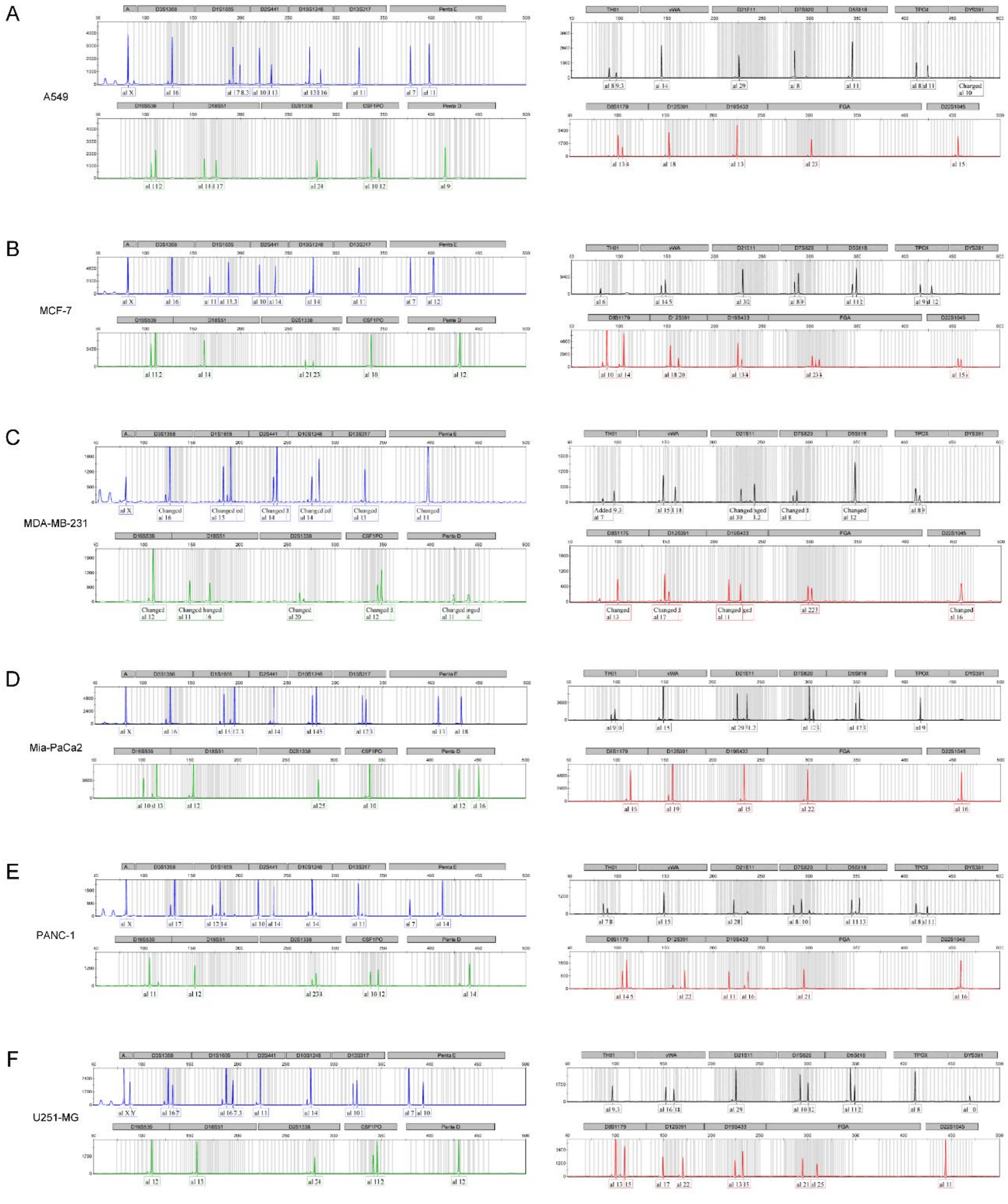
A
A549
B
MCF-7
C
MDA-MB-231
D
Mia-PaCa2
E
PANC-1
F
U251-MG

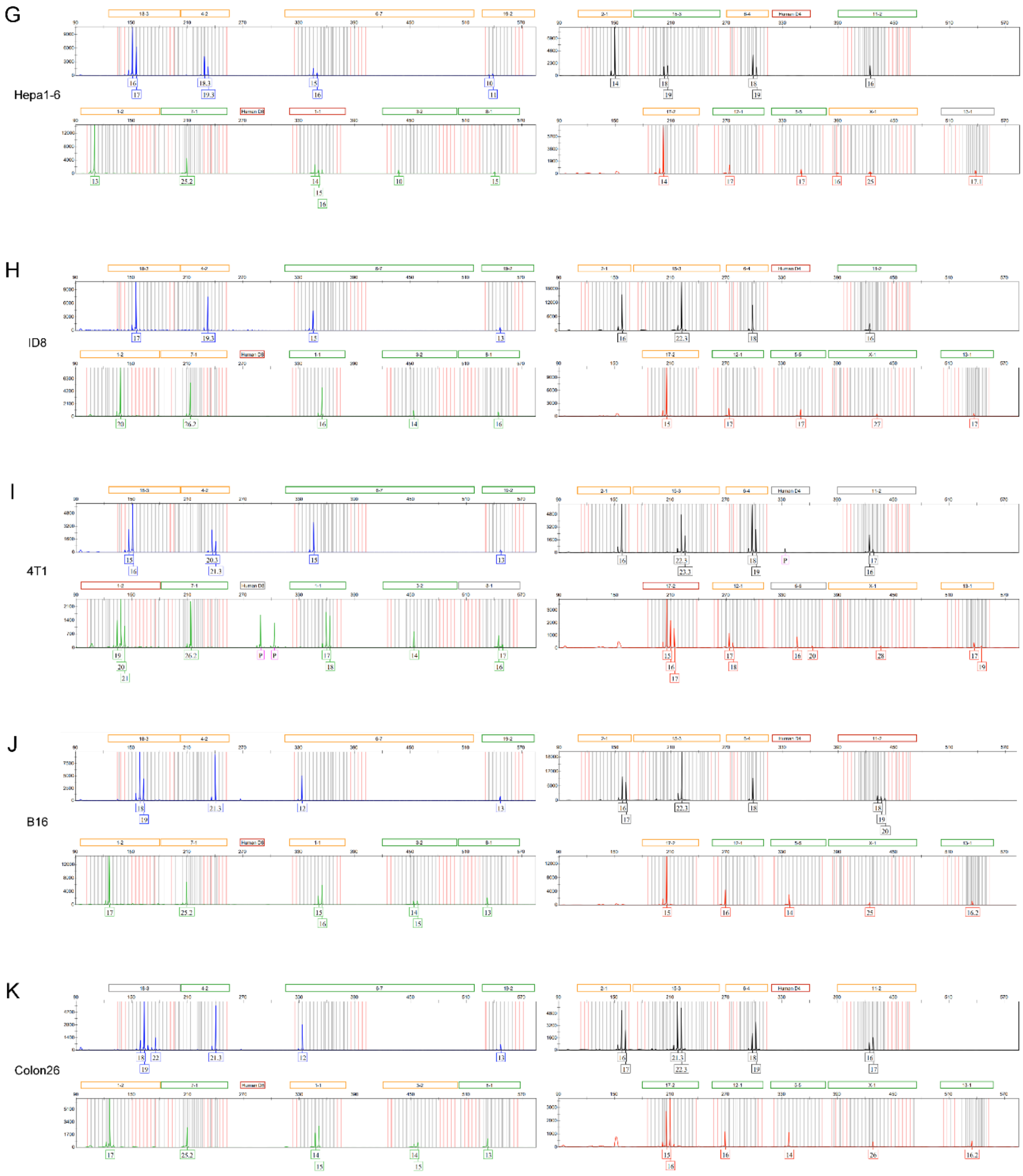
G
Hepa1-6
H
ID8
I
4T1
J
B16
K
Colon26

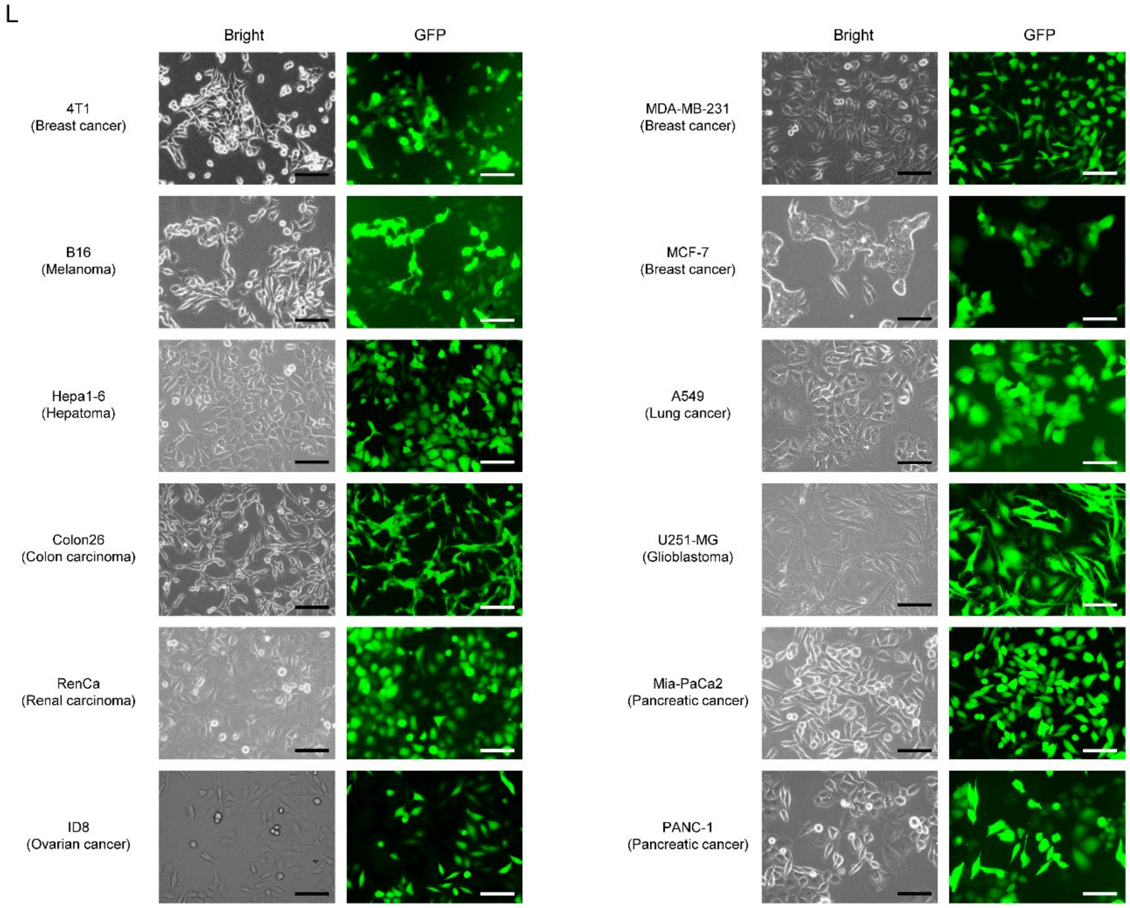

Figure S17. Status of GFP-positive cancer cell lines.

Results of STR analysis of GFP-positive A549 (A), MCF-7 (B), MDA-MB-231 (C), Mia-PaCa2 (D), PANC-1 (E), U251-MG (F), Hepa1-6 (G), ID8 (H), B16 (I), 4T1 (J), and Colon26 (K). STR of all GFP-positive cell lines matched the ATCC and JCRB database. (L) Fluorescence and corresponding bright-field microscope photos of all GFP-expressing cancer cell lines. Scale bar: 50 µm. GFP: green fluorescent protein; STR: The short tandem repeat; JCRB: Japanese Collection of Research Bioresources Cell Bank.

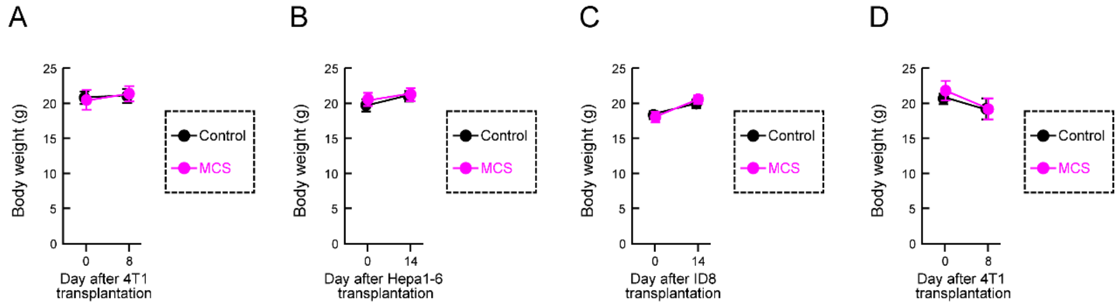

Figure S18. Body weight of mice following transplantation.

Body weight of mice described in **Figure 6 (A), Figure 7 (B, C), and Figure 8 (D)**. Data are presented as the mean ± S.D. (n = 6–8). MCS: microcurrent stimulation.

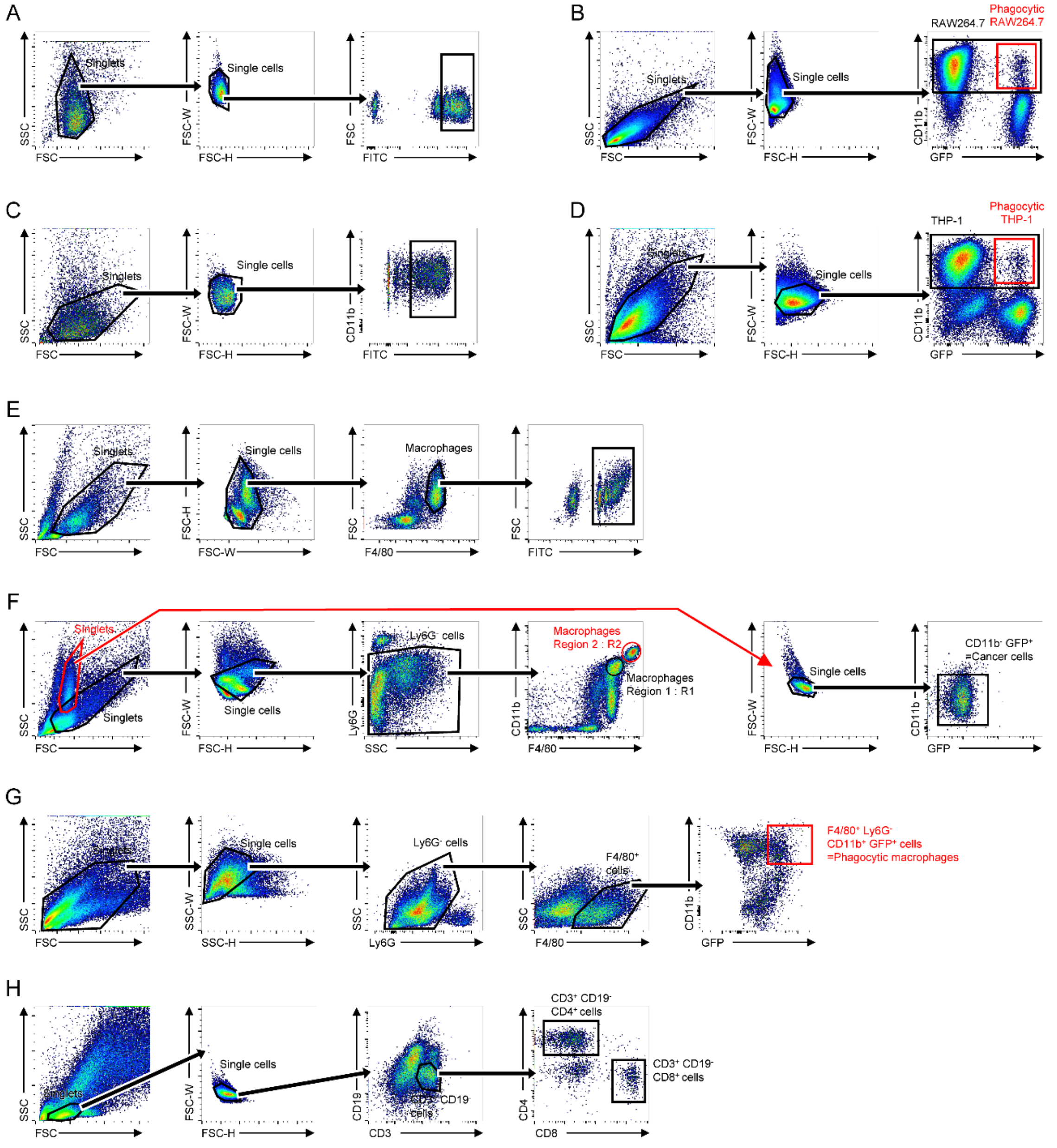

Figure S19. Gating strategies used for flow cytometry analysis.

Gating strategies of (A) RAW254.7 phagocytosed opsonized beads, (B) RAW254.7 phagocytosed GFP-expressing cancer cells, (C) PMA-treated THP-1 phagocytosed opsonized beads, (D) PMA-treated THP-1 phagocytosed GFP-expressing cancer cells, (E) intraperitoneal macrophages cultured with opsonized beads, (F) intraperitoneal F4/80^{+} CD11b^{+} Ly6G^{-} cells and cancer cells of mice implanted with cancer cells in the abdominal cavity, (G) F4/80^{+} CD11b^{+} Ly6G^{-} GFP^{+} cells in tumors collected from the periportal area, and (H) CD3^{+} CD19^{-} CD8^{+} cells and CD3^{+} CD19^{-} CD4^{+} cells in tumors collected from the periportal area. GFP: green fluorescent protein; PMA: phorbol 12-myristate 13-acetate.

Table S1. Gene list using GO analysis in Figure S3 and Figure 2D

Gene Symbol	Genbank accession	Description	Control / MCS	
			log2FC	log2CPM
1110065P20Rik	NM_001142727	RIKEN cDNA 1110065P20 gene	1.920	1.536423
1300017J02Rik	NM_027918	RIKEN cDNA 1300017J02 gene	-1.056	-0.916557
1500011K16Rik	NM_175125	RIKEN cDNA 1500011K16 gene	1.206	3.336269
1600002K03Rik	NM_027207	RIKEN cDNA 1600002K03 gene	1.063	2.113538
1600029I14Rik	NR_028123	RIKEN cDNA 1600029I14 gene	1.171	-0.780272
1700003M07Rik	NR_040647	RIKEN cDNA 1700003M07 gene	1.171	-0.780272
1700007L15Rik	NR_045709	RIKEN cDNA 1700007L15 gene	2.174	0.217760
1700008C04Rik	NR_126468	RIKEN cDNA 1700008C04 gene	-1.188	0.463420
1700019D03Rik	NM_144953	RIKEN cDNA 1700019D03 gene	2.091	-0.918034
1700027J07Rik	NR_040581	RIKEN cDNA 1700027J07 gene	-1.564	0.286776
1700028E10Rik	NR_045699	RIKEN cDNA 1700028E10 gene	-1.600	0.718293
1700028J19Rik	NR_029436	RIKEN cDNA 1700028J19 gene	-1.056	-0.916557
1700034J05Rik	NM_001164236	RIKEN cDNA 1700034J05 gene	1.135	2.599731
1700056E22Rik	NM_028516	RIKEN cDNA 1700056E22 gene	1.141	0.151793
1700066B19Rik	NM_001033168	RIKEN cDNA 1700066B19 gene	-2.202	-0.330362
1700071M16Rik	NR_045444	RIKEN cDNA 1700071M16 gene	1.163	3.879029
1700084J12Rik	NM_028551	ribosomal protein L7-like 1 pseudogene	-1.071	-0.537785
1700099I09Rik	NR_130171	RIKEN cDNA 1700099I09 gene	-1.737	0.808924
1700109K24Rik	NR_108037	RIKEN cDNA 1700109K24 gene	-5.666	-0.915573
1700110C19Rik	NR_045461	RIKEN cDNA 1700110C19 gene	-1.289	-0.430623
2410018L13Rik	NM_028362	RIKEN cDNA 2410018L13 gene	-2.488	-0.149312
2510003B16Rik	NR_131048	RIKEN cDNA 2510003B16 gene	-3.267	-0.329881
2610027K06Rik	NR_077059	RIKEN cDNA 2610027K06 gene	1.044	-0.152647
2610206C17Rik	NR_038175	RIKEN cDNA 2610206C17 gene	-6.074	-0.652047
2610306M01Rik	NR_028298	RIKEN cDNA 2610306M01 gene	1.295	0.459304
2700099C18Rik	NR_024720	NDC80 homolog, kinetochore complex component pseudogene	1.255	0.218690
2900026A02Rik	NM_001347397	RIKEN cDNA 2900026A02 gene	-1.220	0.084442
2900057B20Rik	NR_045365	RIKEN cDNA 2900057B20 gene	-1.976	0.287240
2900076A07Rik	NR_045299	RIKEN cDNA 2900076A07 gene	2.153	-0.240841
3110021A11Rik	NR_030776	RIKEN cDNA 3110021A11 gene	-1.479	-0.330843
3110056K07Rik	NR_045055	RIKEN cDNA 3110056K07 gene	-1.101	3.063772
3110070M22Rik	NM_026084	RIKEN cDNA 3110070M22 gene	-1.533	0.010676
3300005D01Rik	NR_045079	RIKEN cDNA 3300005D01 gene	1.330	0.007372
3830403N18Rik	NM_027510	RIKEN cDNA 3830403N18 gene	-1.955	0.764761
3830432H09Rik	NR_126486	RIKEN cDNA 3830432H09 gene	5.783	-0.781251
4631405J19Rik	NM_001002794	RIKEN cDNA 4631405J19 gene	-1.071	-0.537785
4632428C04Rik	NR_033631	RIKEN cDNA 4632428C04 gene	-1.738	2.565174
4732491K20Rik	NR_045290	RIKEN cDNA 4732491K20 gene	-1.397	-0.067564
4833422M21Rik	NR_130160	RIKEN cDNA 4833422M21 gene	-1.087	0.221948
4921507P07Rik	NM_027564	RIKEN cDNA 4921507P07 gene	-1.369	-0.778803
4921515E04Rik	NR_045711	RIKEN cDNA 4921515E04 gene	1.194	-0.070409
4930405O22Rik	NR_130176	RIKEN cDNA 4930405O22 gene	-1.011	0.669868
4930412C18Rik	NR_030693	RIKEN cDNA 4930412C18 gene	1.855	0.006901
4930417O13Rik	NR_015527	RIKEN cDNA 4930417O13 gene	-1.056	-0.916557
4930486L24Rik	NM_178098	RIKEN cDNA 4930486L24 gene	1.330	0.007372
4930512B01Rik	NR_033573	RIKEN cDNA 4930512B01 gene	-1.079	-0.237971
4930512J16Rik	NR_131021	RIKEN cDNA 4930512J16 gene	-1.369	-0.778803
4930525G20Rik	NR_045194	RIKEN cDNA 4930525G20 gene	-1.845	-0.537299
4930550L24Rik	NM_023774	RIKEN cDNA 4930550L24 gene	1.835	-0.433038
4930562C15Rik	NM_001252142	RIKEN cDNA 4930562C15 gene	-1.458	0.222414
4930564C03Rik	NM_029257	RIKEN cDNA 4930564C03 gene	-2.277	0.156003
4930579K19Rik	NM_175227	RIKEN cDNA 4930579K19 gene	-1.220	0.084442
4931408C20Rik	NM_001033764	RIKEN cDNA 4931408C20 gene	-5.666	-0.915573
4933404K13Rik	NR_131055	RIKEN cDNA 4933404K13 gene	2.091	-0.918034
4933406J10Rik	NR_046004	RIKEN cDNA 4933406J10 gene	2.153	-0.240841
4933416M07Rik	NR_045840	RIKEN cDNA 4933416M07 gene	-2.284	-0.916065
4933417E11Rik	NR_040454	RIKEN cDNA 4933417E11 gene	1.143	1.342511
4933424G05Rik	NR_045372	RIKEN cDNA 4933424G05 gene	1.428	-0.654973
4933433H22Rik	NR_045458	RIKEN cDNA 4933433H22 gene	-1.627	-0.653023
5031425F14Rik	NR_015558	RIKEN cDNA 5031425F14 gene	1.173	3.118620
5031434O11Rik	NR_033624	RIKEN cDNA 5031434O11 gene	-2.202	-0.330362
5430431A17Rik	NR_131001	RIKEN cDNA 5430431A17 gene	1.428	-0.654973
5730405O15Rik	NR_038158	RIKEN cDNA 5730405O15 gene	-2.727	0.011621
5730460C07Rik	NR_045801	RIKEN cDNA 5730460C07 gene	2.091	-0.918034
6330415G19Rik	NR_126484	RIKEN cDNA 6330415G19 gene	-2.171	0.085383
6330418K02Rik	NR_045821	RIKEN cDNA 6330418K02 gene	1.076	0.513996
6430550D23Rik	NM_001145351	RIKEN cDNA 6430550D23 gene	-1.079	-0.237971
9130019P16Rik	NM_198118	RIKEN cDNA 9130019P16 gene	-1.087	0.221948
9230116L04Rik	NR_110486	RIKEN cDNA 9230116L04 gene	1.445	-0.240363
9230116N13Rik	NR_024328	RIKEN cDNA 9230116N13 gene	1.343	0.845579
9330159M07Rik	NR_037982	RIKEN cDNA 9330159M07 gene	-1.260	0.670318
9630028B13Rik	NM_176943	RIKEN cDNA 9630028B13 gene	1.194	-0.070409
9830107B12Rik	NM_001177896	RIKEN cDNA 9830107B12 gene	1.111	1.217317
A630019I02Rik	NR_046182	RIKEN cDNA A630019I02 gene	-1.587	1.289054
A930007I19Rik	NR_015567	RIKEN cDNA A930007I19 gene	-1.344	0.155067
A930019D19Rik	NR_040619	RIKEN cDNA A930019D19 gene	-1.071	-0.537785
AA413626	NR_102683	ribosomal protein L17 pseudogene	-1.056	-0.916557

AA986860	NM_177604	expressed sequence AA986860	1.773	0.282141
AI661453	NM_145489	expressed sequence AI661453	-1.079	-0.237971
AW551984	NM_001199556	expressed sequence AW551984	2.091	-0.918034
Aanat	NM_009591	arylalkylamine N-acetyltransferase	1.639	0.458847
Abca13	NM_178259	ATP-binding cassette, sub-family A (ABC1), member 13	5.783	-0.781251
Abcb4	NM_008830	ATP-binding cassette, sub-family B (MDR/TAP), member 4	-1.087	0.221948
Acer1	NM_175731	alkaline ceramidase 1	-1.056	-0.916557
Acnat1	NM_001164565	acyl-coenzyme A amino acid N-acyltransferase 1	1.171	-0.780272
Acpp	NM_019807	acid phosphatase, prostate	1.183	1.150163
Acr	NM_001205049	acrosin prepropeptide	-6.074	-0.652047
Acrbp	NM_001127340	proacrosin binding protein	-1.019	3.090596
Acyp1	NM_025421	acylphosphatase 1, erythrocyte (common) type	1.189	2.636477
Adamts3	NM_001081401	a disintegrin-like and metallopeptidase (reprolysin type) with thrombospondin type 1 motif, 3	2.091	-0.918034
Adgrd1	NM_001081342	adhesion G protein-coupled receptor D1	1.428	-0.654973
Adgrf3	NM_001014394	adhesion G protein-coupled receptor F3	2.091	-0.918034
Adgrg5	NM_001033468	adhesion G protein-coupled receptor G5	1.350	3.537420
Adm	NM_009627	adrenomedullin	-1.897	1.381209
Agbl1	NM_001199224	ATP/GTP binding protein-like 1	-1.278	3.154374
Ager	NM_001271422	advanced glycosylation end product-specific receptor	1.044	-0.152647
Airn	NR_002853	antisense Igf2r RNA	-1.627	-0.653023
Akap14	NM_001033785	A kinase (PRKA) anchor protein 14	-1.056	-0.916557
Akap5	NM_001101471	A kinase (PRKA) anchor protein 5	-1.457	3.000019
Alyref2	NM_019484	Aly/REF export factor 2	-1.096	1.190298
Amacr	NM_008537	alpha-methylacyl-CoA racemase	1.135	2.599731
Angptl2	NM_011923	angiopoietin-like 2	-1.184	1.519887
Angptl4	NM_020581	angiopoietin-like 4	2.112	0.512632
Angptl7	NM_001039554	angiopoietin-like 7	-1.679	1.573956
Ank3	NM_009670	ankyrin 3, epithelial	2.091	-0.918034
Ankle1	NM_001310502	ankyrin repeat and LEM domain containing 1	1.044	-0.152647
Ankmy1	NM_001347091	ankyrin repeat and MYND domain containing 1	2.348	-0.780762
Ankrd34b	NM_175455	ankyrin repeat domain 34B	-1.369	-0.778803
Ankrd66	NM_001254953	ankyrin repeat domain 66	1.381	0.513541
Apbb2	NM_001201413	amyloid beta (A4) precursor protein-binding, family B, member 2	-1.797	-0.149789
Apcs	NM_011318	serum amyloid P-component	-1.056	-0.916557
Apof	NM_133997	apolipoprotein F	1.171	-0.780272
Apoh	NM_013475	apolipoprotein H	1.017	0.081623
Apol10b	NM_177820	apolipoprotein L 10B	-1.089	0.406607
Apol7c	NM_175391	apolipoprotein L 7c	-5.666	-0.915573
Ar	NM_013476	androgen receptor	1.518	1.248706
Arap3	NM_001205336	ArfGAP with RhoGAP domain, ankyrin repeat and PH domain 3	1.267	4.579340
Arhgap19	NM_001163495	Rho GTPase activating protein 19	1.104	0.344252
Arhgap8	NM_001164627	Rho GTPase activating protein 8	-1.234	1.465457
Asap3	NM_001008232	ArfGAP with SH3 domain, ankyrin repeat and PH domain 3	-1.056	-0.916557
Asb14	NM_001170748	ankyrin repeat and SOCS box-containing 14	-1.201	0.286312
Asgr2	NM_001313925	asialoglycoprotein receptor 2	-2.284	-0.916065
Ass1	NM_007494	argininosuccinate synthetase 1	1.133	4.666438
Atad3aos	NM_175157	ATPase family, AAA domain containing 3A, opposite strand	1.089	-0.432555
Atg16l2	NM_001111111	autophagy related 16-like 2 (S. cerevisiae)	-1.010	3.161139
Atp1a2	NM_178405	ATPase, Na+/K+ transporting, alpha 2 polypeptide	-1.369	-0.778803
Atp1b1	NM_009721	ATPase, Na+/K+ transporting, beta 1 polypeptide	-1.522	1.743977
Atp1b4	NM_001290389	ATPase, (Na+)/K+ transporting, beta 4 polypeptide	1.171	-0.780272
Atp5e	NM_025983	ATP synthase, H+ transporting, mitochondrial F1 complex, epsilon subunit	1.349	5.509464
Atp6v0c-ps2	NR_037854	ATPase, H+ transporting, lysosomal V0 subunit C, pseudogene 2	11.405	4.201016
Atp6v1c2	NM_001159632	ATPase, H+ transporting, lysosomal V1 subunit C2	-1.289	-0.430623
Atp6v1g2	NM_001347351	ATPase, H+ transporting, lysosomal V1 subunit G2	-1.039	1.156142
Auts2	NM_177047	autism susceptibility candidate 2	-2.337	0.809813
Axin2	NM_015732	axin 2	1.361	2.623357
B230216N24Rik	NR_037993	RIKEN cDNA B230216N24 gene	-1.845	-0.537299
B230217O12Rik	NR_040316	RIKEN cDNA B230217O12 gene	-1.150	1.223672
B3gnt4	NM_198611	UDP-GlcNAc:betaGal beta-1,3-N-acetylglucosaminyltransferase 4	2.091	-0.918034
B3gnt6	NM_001081167	UDP-GlcNAc:betaGal beta-1,3-N-acetylglucosaminyltransferase 6 (core 3 synthase)	-1.479	-0.330843
B430212C06Rik	NR_033214	RIKEN cDNA B430212C06 gene	1.552	0.402473
B4galnt4	NM_177897	beta-1,4-N-acetyl-galactosaminyl transferase 4	-1.369	-0.778803
B9d1	NM_001330780	B9 protein domain 1	-5.666	-0.915573
BC002163	NR_002445	NADH dehydrogenase Fe-S protein 5 pseudogene	3.350	1.480885
BC025920	NM_001033363	zinc finger protein pseudogene	-1.280	0.518095
BC030867	NM_153544	cDNA sequence BC030867	1.171	-0.780272
BC037032	NR_028266	cDNA Sequence BC037032	-2.034	-0.430140
BC051226	NR_045146	cDNA sequence BC051226	-1.089	0.406607
BC052688	NR_028430	cDNA sequence BC052688	1.171	-0.780272
Bag2	NM_145392	BCL2-associated athanogene 2	-1.369	-0.778803
Bard1	NM_007525	BRCA1 associated RING domain 1	1.053	2.413427
Bcl2l15	NM_001142959	BCLl2-like 15	-1.056	-0.916557
Best1	NM_011913	bestrophin 1	1.428	-0.654973
Boll	NM_001113367	boule homolog, RNA binding protein	-1.056	-0.916557
Brdt	NM_001079873	bromodomain, testis-specific	1.428	-0.654973
Brsk2	NM_001009929	BR serine/threonine kinase 2	-1.198	1.895304
Btbd11	NM_001017525	BTB (POZ) domain containing 11	-1.079	-0.237971
Bub1b	NM_009773	BUB1B, mitotic checkpoint serine/threonine kinase	1.545	1.613734
C1qtnf5	NM_001040631	C1q and tumor necrosis factor related protein 5	1.460	0.343791
C1rb	NM_001113356	complement component 1, r subcomponent B	-2.284	-0.916065
C1rl	NM_181344	complement component 1, r subcomponent-like	1.386	3.808859
C1s1	NM_001097617	complement component 1, s subcomponent 1	1.017	0.081623

C330013E15Rik	NR_045701	RIKEN cDNA C330013E15 gene	1.044	-0.152647
C430049B03Rik	NR_038184	RIKEN cDNA C430049B03 gene	1.361	0.282604
C4bp	NM_007576	complement component 4 binding protein	1.171	-0.780272
C5ar2	NM_001146005	complement component 5a receptor 2	-1.163	0.892917
Cacna1f	NM_019582	calcium channel, voltage-dependent, alpha 1F subunit	1.646	-0.539725
Cacna1i	NM_001044308	calcium channel, voltage-dependent, alpha 1I subunit	-1.042	6.867565
Cacnb2	NM_001252533	calcium channel, voltage-dependent, beta 2 subunit	1.171	-0.780272
Cacnb3	NM_001044741	calcium channel, voltage-dependent, beta 3 subunit	2.075	0.150859
Capn12	NM_001110807	calpain 12	2.566	-0.655460
Capn3	NM_001109761	calpain 3	-2.202	-0.330362
Car12	NM_001306148	carbonic anhydrase 12	1.044	-0.152647
Car2	NM_001357334	carbonic anhydrase 2	-1.657	0.084912
Car3	NM_007606	carbonic anhydrase 3	-1.397	-0.067564
Car9	NM_139305	carbonic anhydrase 9	1.773	0.282141
Casc1	NM_177222	cancer susceptibility candidate 1	1.445	-0.240363
Caskin1	NM_027937	CASK interacting protein 1	1.343	0.845579
Cbln3	NM_019820	cerebellin 3 precursor protein	-1.369	-0.778803
Ccdc116	NM_001164606	coiled-coil domain containing 116	-1.084	0.010204
Ccdc146	NM_029195	coiled-coil domain containing 146	2.091	-0.918034
Ccdc155	NM_201374	coiled-coil domain containing 155	2.091	-0.918034
Ccdc169	NM_001290138	coiled-coil domain containing 169	1.835	-0.433038
Ccdc18	NM_028481	coiled-coil domain containing 18	2.003	-0.333727
Ccdc28b	NM_025455	coiled coil domain containing 28B	1.019	2.523262
Ccdc80	NM_026439	coiled-coil domain containing 80	1.710	1.924143
Ccdc89	NM_027298	coiled-coil domain containing 89	2.348	-0.780762
Ccl4	NM_013652	chemokine (C-C motif) ligand 4	-3.267	-0.329881
Ccnb2	NM_007630	cyclin B2	1.139	1.614933
Ccpg1os	NM_001198789	cell cycle progression 1, opposite strand	1.295	0.459304
Ccr3	NM_009914	chemokine (C-C motif) receptor 3	4.668	4.170687
Ccs	NM_016892	copper chaperone for superoxide dismutase	1.173	3.313859
Cd163l1	NM_172909	CD163 molecule-like 1	-2.171	0.085383
Cd207	NM_144943	CD207 antigen	-1.071	-0.537785
Cd209g	NM_027343	CD209g antigen	-1.369	-0.778803
Cd3d	NM_013487	CD3 antigen, delta polypeptide	1.014	1.043833
Cd46	NM_010778	CD46 antigen, complement regulatory protein	1.646	-0.539725
Cd8b1	NM_009858	CD8 antigen, beta chain 1	1.133	0.712922
Cdc42ep1	NM_027219	CDC42 effector protein (Rho GTPase binding) 1	1.428	-0.654973
Cdca5	NM_026410	cell division cycle associated 5	2.112	0.512632
Cdh22	NM_174988	cadherin 22	-5.885	-0.777822
Cdh3	NM_001037809	cadherin 3	-1.369	-0.778803
Cdk1	NM_007659	cyclin-dependent kinase 1	1.235	1.183918
Cdk5r1	NM_009871	cyclin-dependent kinase 5, regulatory subunit 1 (p35)	1.235	1.183918
Ceacam2	NM_001113368	carcinoembryonic antigen-related cell adhesion molecule 2	-2.727	0.011621
Cebpe	NM_207131	CCAAT/enhancer binding protein (C/EBP), epsilon	1.227	2.040919
Celf4	NM_001146292	CUGBP, Elav-like family member 4	-1.416	1.288633
Cenpe	NM_173762	centromere protein E	1.069	2.469222
Cenph	NM_021886	centromere protein H	1.835	-0.433038
Cenpm	NM_001080158	centromere protein M	1.171	-0.780272
Cep55	NM_001164362	centrosomal protein 55	1.978	1.113878
Cercam	NM_207298	cerebral endothelial cell adhesion molecule	1.126	1.687559
Ces1c	NM_007954	carboxylesterase 1C	-1.087	0.221948
Cetn4	NM_145825	centrin 4	-1.071	-0.537785
Cfap126	NM_001081275	cilia and flagella associated protein 126	-1.369	-0.778803
Cfhr1	NM_015780	complement factor H-related 1	-2.034	-0.430140
Cfi	NM_001329552	complement component factor i	-2.949	-0.536813
Cgn	NM_001037711	cingulin	1.044	-0.152647
Cgref1	NM_001160149	cell growth regulator with EF hand domain 1	-1.289	-0.430623
Chac1	NM_026929	ChaC, cation transport regulator 1	-1.056	-0.916557
Chadl	NM_001164320	chondroadherin-like	-1.627	-0.653023
Chaf1a	NM_013733	chromatin assembly factor 1, subunit A (p150)	1.530	2.597764
Chaf1b	NM_028083	chromatin assembly factor 1, subunit B (p60)	1.468	0.927644
Chil1	NM_007695	chitinase-like 1	-1.089	0.406607
Chrm4	NM_007699	cholinergic receptor, muscarinic 4	2.091	-0.918034
Chst1	NM_001356552	carbohydrate (keratan sulfate Gal-6) sulfotransferase 1	-1.269	2.337535
Chst13	NM_027928	carbohydrate (chondroitin 4) sulfotransferase 13	3.263	1.940487
Chsy3	NM_001081328	chondroitin sulfate synthase 3	1.245	0.616671
Ckap2l	NM_181589	cytoskeleton associated protein 2-like	1.276	1.864373
Cks1b	NM_016904	CDC28 protein kinase 1b	1.193	1.564262
Cldn22	NM_029383	claudin 22	-1.479	-0.330843
Cldn3	NM_009902	claudin 3	2.566	-0.655460
Clec2g	NM_001168223	C-type lectin domain family 2, member g	-3.024	0.223813
Clec4g	NM_029465	C-type lectin domain family 4, member g	-2.612	-0.066614
Clnk	NM_013748	cytokine-dependent hematopoietic cell linker	-1.056	-0.916557
Clrn1	NM_153384	clarin 1	-1.369	-0.778803
Clu	NM_013492	clusterin	1.025	0.928519
Cmtm1	NM_181990	CKLF-like MARVEL transmembrane domain containing 1	5.565	-0.918525
Col1a1	NM_007742	collagen, type I, alpha 1	1.569	0.151326
Col1a2	NM_007743	collagen, type I, alpha 2	2.353	0.342870
Col3a1	NM_009930	collagen, type III, alpha 1	2.616	0.885444
Col4a4	NM_007735	collagen, type IV, alpha 4	1.171	-0.780272
Col4a5	NM_001163155	collagen, type IV, alpha 5	1.654	1.883820
Col6a1	NM_009933	collagen, type VI, alpha 1	-1.079	-0.237971
Coro2b	NM_175484	coronin, actin binding protein, 2B	-1.397	-0.067564

Cox7a2	NM_009945	cytochrome c oxidase subunit VIIa 2	1.082	4.756301
Cpe	NM_013494	carboxypeptidase E	-1.056	-0.916557
Cpne5	NM_153166	copine V	-1.479	-0.330843
Cpne7	NM_170684	copine VII	-1.369	-0.778803
Cpne9	NM_170673	copine family member IX	-1.627	-0.653023
Crmp1	NM_001136058	collapsin response mediator protein 1	-1.230	3.064642
Cryab	NM_001289782	crystallin, alpha B	1.454	0.081154
Ctsk	NM_007802	cathepsin K	-2.352	-0.237013
Ctsw	NM_009985	cathepsin W	1.111	1.217317
Ctxn1	NM_183315	cortexin 1	-1.369	-0.778803
Cx3cl1	NM_009142	chemokine (C-X3-C motif) ligand 1	1.171	-0.780272
Cxcl1	NM_008176	chemokine (C-X-C motif) ligand 1	-1.140	2.600769
Cxcl13	NM_018866	chemokine (C-X-C motif) ligand 13	-2.047	8.084261
Cxcl3	NM_203320	chemokine (C-X-C motif) ligand 3	-2.715	0.465252
Cyb5r2	NM_001205227	cytochrome b5 reductase 2	-2.034	-0.430140
Cyb5rl	NM_001346552	cytochrome b5 reductase-like	1.212	1.663491
Cyp2c67	NM_001024719	cytochrome P450, family 2, subfamily c, polypeptide 67	2.348	-0.780762
Cyp2d9	NM_010006	cytochrome P450, family 2, subfamily d, polypeptide 9	-1.369	-0.778803
Cyp2j9	NM_028979	cytochrome P450, family 2, subfamily j, polypeptide 9	-2.488	-0.149312
Cyp39a1	NM_001285947	cytochrome P450, family 39, subfamily a, polypeptide 1	-1.309	1.832340
D130040H23Rik	NM_172491	RIKEN cDNA D130040H23 gene	-1.004	3.564427
D5Ertd605e	NR_033625	DNA segment, Chr 5, ERATO Doi 605, expressed	2.348	-0.780762
D630045J12Rik	NM_194061	RIKEN cDNA D630045J12 gene	-1.369	-0.778803
D7Ertd143e	NR_028425	DNA segment, Chr 7, ERATO Doi 143, expressed	-1.056	-0.916557
Dach1	NM_001038610	dachshund family transcription factor 1	3.574	0.800056
Dapk2	NM_010019	death-associated protein kinase 2	2.731	0.150392
Dbp	NM_016974	D site albumin promoter binding protein	-1.600	3.711966
Ddr2	NM_022563	discoidin domain receptor family, member 2	1.646	-0.539725
Depdc1b	NM_178683	DEP domain containing 1B	1.175	0.887639
Dlec1	NM_177117	deleted in lung and esophageal cancer 1	-1.845	-0.537299
Dlg5	NM_001163513	discs large MAGUK scaffold protein 5	-1.359	1.546960
Dnaaf1	NM_026648	dynein, axonemal assembly factor 1	1.171	-0.780272
Dnah5	NM_133365	dynein, axonemal, heavy chain 5	-3.403	-0.236534
Dnah6	NM_001164669	dynein, axonemal, heavy chain 6	-2.284	-0.916065
Dnm3	NM_001038619	dynamin 3	-1.220	0.084442
Dpp6	NM_001136060	dipeptidylpeptidase 6	-1.344	0.155067
Dsg2	NM_007883	desmoglein 2	-2.284	-0.916065
Dsp	NM_023842	desmoplakin	-1.527	0.670768
Dtl	NM_001305233	denticleless E3 ubiquitin protein ligase	1.499	2.230
Dtx1	NM_008052	deltex 1, E3 ubiquitin ligase	-1.487	5.211
Dusp14	NM_019819	dual specificity phosphatase 14	-1.071	-0.538
Dusp19	NM_024438	dual specificity phosphatase 19	1.257	1.312
Dusp8	NM_008748	dual specificity phosphatase 8	-1.247	-0.150
Dynlt1b	NM_009342	dynein light chain Tctex-type 1B	1.007	5.267
Dynlt1f	NM_001166627	dynein light chain Tctex-type 1F	2.592	1.586
E130008D07Rik	NR_045153	RIKEN cDNA E130008D07 gene	-5.666	-0.916
E130311K13Rik	NM_177856	RIKEN cDNA E130311K13 gene	-1.243	1.380
E230029C05Rik	NR_015614	RIKEN cDNA E230029C05 gene	-1.397	-0.068
E330023G01Rik	NR_045332	RIKEN cDNA E330023G01 gene	-1.056	-0.917
Echdc3	NM_024208	enoyl Coenzyme A hydratase domain containing 3	-1.254	1.995
Efhc1	NM_027974	EF-hand domain (C-terminal) containing 1	1.044	-0.153
Efnb3	NM_007911	ephrin B3	1.171	-0.780
Efs	NM_010112	embryonal Fyn-associated substrate	1.447	1.905
Egfl8	NM_152922	EGF-like domain 8	1.646	-0.540
Egr1	NM_007913	early growth response 1	-1.274	4.524
Egr2	NM_001347458	early growth response 2	-1.152	2.824
Egr3	NM_001289925	early growth response 3	-2.217	2.763
Ehf	NM_007914	ets homologous factor	-1.845	-0.537
Ell3	NM_145973	elongation factor RNA polymerase II-like 3	-1.142	2.523
Elovl6	NM_130450	ELOVL family member 6, elongation of long chain fatty acids (yeast)	1.059	1.184
Emid1	NM_080595	EMI domain containing 1	1.171	-0.780
Emp2	NM_007929	epithelial membrane protein 2	1.171	-0.780
Endou	NM_001168693	endonuclease, polyU-specific	-2.202	-0.330
Enpp2	NM_001136077	ectonucleotide pyrophosphatase/phosphodiesterase 2	1.835	-0.433
Entpd2	NM_009849	ectonucleoside triphosphate diphosphohydrolase 2	2.091	-0.918
Epdr1	NM_134065	ependymin related protein 1 (zebrafish)	-1.627	-0.653
Epor	NM_010149	erythropoietin receptor	2.348	-0.781
Eps8l1	NM_001290416	EPS8-like 1	1.044	-0.153
Erbb2	NM_001003817	erb-b2 receptor tyrosine kinase 2	1.039	0.803
Ercc8	NM_028042	excision repaiross-complementing rodent repair deficiency, complementation group 8	1.021	2.248
Esam	NM_027102	endothelial cell-specific adhesion molecule	2.091	-0.918
Esm1	NM_023612	endothelial cell-specific molecule 1	2.348	-0.781
Etv4	NM_001316365	ets variant 4	1.835	-0.433
Evc2	NM_145920	EvC ciliary complex subunit 2	-1.056	-0.917
Evpl	NM_025276	envoplakin	1.171	-0.780
Eya1	NM_001252192	EYA transcriptional coactivator and phosphatase 1	-1.093	0.851
F2r	NM_010169	coagulation factor II (thrombin) receptor	1.507	1.686
F630042J09Rik	NR_033540	RIKEN cDNA F630042J09 gene	1.361	0.283
F730043M19Rik	NR_015602	RIKEN cDNA F730043M19 gene	1.428	-0.655
F9	NM_001305797	coagulation factor IX	-1.056	-0.917
Fabp5	NM_001272097	fatty acid binding protein 5, epidermal	1.694	1.613
Fabp7	NM_021272	fatty acid binding protein 7, brain	1.416	5.659
Fads6	NM_178035	fatty acid desaturase domain family, member 6	1.171	-0.780

Fam124a	NM_001243857	family with sequence similarity 124, member A	-1.369	-0.779
Fam160a1	NM_172682	family with sequence similarity 160, member A1	-1.236	1.854
Fam166b	NM_001162381	family with sequence similarity 166, member B	-1.289	-0.431
Fam198a	NM_001199927	family with sequence similarity 198, member A	-3.403	-0.237
Fam92b	NM_001033980	family with sequence similarity 92, member B	1.428	-0.655
Fbxl15	NM_133694	F-box and leucine-rich repeat protein 15	1.210	1.281
Fbxo36	NM_025386	F-box protein 36	-1.056	-0.917
Fbxw13	NM_177598	F-box and WD-40 domain protein 13	-1.417	1.494
Fcgr4	NM_144559	Fc receptor, IgG, low affinity IV	1.127	5.855
Fcmr	NM_026976	Fc fragment of IgM receptor	-1.416	6.130
Fcrlb	NM_001029984	Fc receptor-like B	2.566	-0.655
Fdx1l	NM_001039824	ferredoxin 1-like	1.227	2.041
Fer1l5	NM_001277076	fer-1-like 5 (C. elegans)	-1.289	-0.431
Fetub	NM_001083904	fetuin beta	1.428	-0.655
Ffar1	NM_194057	free fatty acid receptor 1	-1.416	1.289
Fgf1	NM_010197	fibroblast growth factor 1	-1.247	-0.150
Fgf11	NM_001291104	fibroblast growth factor 11	-1.614	1.721
Fgfr3	NM_001163215	fibroblast growth factor receptor 3	-1.247	-0.150
Fgl1	NM_145594	fibrinogen-like protein 1	1.445	-0.240
Fhit	NM_001308285	fragile histidine triad gene	-1.289	-0.431
Fibcd1	NM_178887	fibrinogen C domain containing 1	-1.845	-0.537
Flt4	NM_008029	FMS-like tyrosine kinase 4	-1.028	0.933
Foxc1	NM_008592	forkhead box C1	1.428	-0.655
Foxc2	NM_013519	forkhead box C2	-1.845	-0.537
Foxd2	NM_008593	forkhead box D2	-1.397	-0.068
Foxd2os	NR_030721	forkhead box D2, opposite strand	1.171	-0.780
Frmpd4	NM_001033330	FERM and PDZ domain containing 4	-1.797	-0.150
Fsbp	NM_001256142	fibrinogen silencer binding protein	5.972	-0.656
Fsd2	NM_172904	fibronectin type III and SPRY domain containing 2	-1.845	-0.537
Fxyd6	NM_022004	FXYD domain-containing ion transport regulator 6	2.008	0.712
G0s2	NM_008059	G0/G1 switch gene 2	2.003	-0.334
G730013B05Rik	NR_040379	RIKEN cDNA G730013B05 gene	1.278	-0.333
Gabbr2	NM_001081141	gamma-aminobutyric acid (GABA) B receptor, 2	-1.620	1.050
Gad2	NM_008078	glutamic acid decarboxylase 2	-1.369	-0.779
Gadd45g	NM_011817	growth arrest and DNA-damage-inducible 45 gamma	1.089	0.968
Gal3st3	NM_001024717	galactose-3-O-sulfotransferase 3	-1.188	0.463
Galr2	NM_010254	galanin receptor 2	2.091	-0.918
Gas6	NM_019521	growth arrest specific 6	-1.620	1.050
Gdpd3	NM_024228	glycerophosphodiester phosphodiesterase domain containing 3	1.445	-0.240
Gdpd5	NM_201352	glycerophosphodiester phosphodiesterase domain containing 5	1.460	0.344
Ggn	NM_182694	gametogenetin	1.466	0.758
Ggnbp2os	NR_131197	gametogenetin binding protein 2, opposite strand	-1.280	0.518
Gh	NM_008117	growth hormone	-2.034	-0.430
Gins1	NM_001163476	GINS complex subunit 1 (Psf1 homolog)	1.731	-0.071
Gins2	NM_178856	GINS complex subunit 2 (Psf2 homolog)	1.526	0.967
Gja1	NM_010288	gap junction protein, alpha 1	1.445	-0.240
Gjb1	NM_001302496	gap junction protein, beta 1	-1.056	-0.917
Gli1	NM_010296	GLI-Kruppel family member GLI1	1.014	1.044
Glis3	NM_001305671	GLIS family zinc finger 3	-1.479	-0.331
Gm10046	NR_033484	predicted gene 10046	1.646	-0.540
Gm10638	NR_027829	predicted gene 10638	2.091	-0.918
Gm10677	NR_046048	predicted gene 10677	-2.284	-0.916
Gm10768	NR_033472	predicted gene 10768	2.091	-0.918
Gm11346	NR_024599	X-linked lymphocyte-regulated 5 pseudogene	-1.067	1.936
Gm11545	NM_001105561	predicted gene 11545	-1.079	-0.238
Gm11944	NR_045708	predicted gene 11944	-2.171	0.085
Gm12159	NR_045100	predicted gene 12159	2.091	-0.918
Gm12505	NR_040674	predicted gene 12505	1.171	-0.780
Gm12522	NR_040560	predicted gene 12522	2.091	-0.918
Gm128	NM_001024841	predicted gene 128	1.381	0.514
Gm13830	NR_131932	predicted gene 13830	-1.369	-0.779
Gm13889	NM_001145034	predicted gene 13889	-1.369	-0.779
Gm13986	NR_126479	predicted gene 13986	1.865	0.343
Gm14308	NM_001099349	predicted gene 14308	-1.644	4.434
Gm14327	NR_038101	predicted gene 14327	-1.316	1.224
Gm14405	NR_040256	predicted gene 14405	-1.369	-0.779
Gm14430	NM_001100415	predicted gene 14430	5.725	3.654
Gm14434	NM_001101804	predicted gene 14434	7.542	0.563
Gm15441	NR_040409	predicted gene 15441	2.266	0.282
Gm15612	NR_045880	predicted gene 15612	-1.498	0.464
Gm15708	NR_040432	predicted gene 15708	-2.193	2.055
Gm16062	NR_045686	predicted gene 16062	1.017	0.082
Gm16223	NR_131130	predicted gene 16223	1.104	0.344
Gm16367	NM_001031622	predicted gene 16367	-1.627	-0.653
Gm16386	NR_030709	zinc finger protein 946 pseudogene	-1.152	2.824
Gm1653	NR_040591	predicted gene 1653	-3.117	-0.430
Gm16701	NR_037988	predicted gene, 16701	3.073	-0.334
Gm16712	NR_108021	predicted gene, 16712	-1.797	-0.150
Gm16845	NR_040406	predicted gene, 16845	-1.243	1.380
Gm16998	NR_038016	predicted gene, 16998	-1.369	-0.779
Gm17757	NR_040453	GTPase, very large interferon inducible 1 pseudogene	2.239	5.903
Gm17769	NR_027377	predicted gene, 17769	-1.369	-0.779
Gm18853	NR_040456	GTPase, very large interferon inducible 1 pseudogene	-12.831	5.589

Gm19345	NM_001270489	predicted gene, 19345	-1.071	-0.538
Gm20187	NR_045052	predicted gene, 20187	-2.202	-0.330
Gm20324	NR_045068	predicted gene, 20324	-1.797	-0.150
Gm20337	NR_045057	predicted gene, 20337	-1.627	-0.653
Gm21992	NM_001290127	predicted gene 21992	-5.666	-0.916
Gm2848	NR_046069	predicted gene 2848	-1.035	1.915
Gm30505	NR_110508	predicted gene, 30505	1.089	-0.433
Gm3704	NR_131166	predicted gene 3704	-1.289	-0.431
Gm38426	NR_103491	predicted gene, 38426	-1.089	0.407
Gm4371	NR_028311	eukaryotic translation initiation factor 3, subunit I pseudogene	-1.289	-0.431
Gm4532	NR_030674	predicted gene 4532	-3.267	-0.330
Gm4791	NM_001243258	predicted gene 4791	-1.056	-0.917
Gm5088	NR_002862	poly(A)-binding protein, cytoplasmic pseudogene	-1.010	2.014
Gm5424	NR_002687	argininosuccinate synthase pseudogene	-1.564	4.677
Gm5434	NM_001014396	ubiquitin-conjugating enzyme E2F (putative) pseudogene	1.044	-0.153
Gm5464	NM_001034881	predicted gene 5464	-1.585	0.519
Gm5512	NR_002891	required for meiotic nuclear division 1 pseudogene	-2.034	-0.430
Gm5523	NR_004447	glyceraldehyde-3-phosphate dehydrogenase pseudogene	-2.277	0.156
Gm6093	NR_131140	predicted gene 6093	3.333	-0.154
Gm6377	NM_001037917	predicted gene 6377	-1.726	1.123
Gm6644	NR_028277	Akr1b3 pseudogene	12.240	5.022
Gm8221	NR_033577	apolipoprotein L 7c pseudogene	-2.113	2.618
Gm8369	NM_001164202	predicted gene 8369	-1.066	1.874
Gm9054	NR_045872	predicted gene 9054	2.185	0.565
Gm9079	NR_004052	transmembrane emp24 domain trafficking protein 2 pseudogene	-1.280	0.518
Gm973	NM_001013771	predicted gene 973	-1.845	-0.537
Gm9733	NM_001076679	predicted gene 9733	1.202	0.403
Gm9958	NR_045618	predicted gene 9958	-1.044	1.256
Gnao1	NM_001113384	guanine nucleotide binding protein, alpha O	-1.797	-0.150
Gnat2	NM_008141	guanine nucleotide binding protein, alpha transducing 2	1.445	-0.240
Gnb5	NM_010313	guanine nucleotide binding protein (G protein), beta 5	1.111	1.217
Gng11	NM_025331	guanine nucleotide binding protein (G protein), gamma 11	2.731	0.150
Gpbar1	NM_174985	G protein-coupled bile acid receptor 1	5.565	-0.919
Gpc6	NM_001079844	glypican 6	1.637	1.043
Gpr1	NM_001357045	G protein-coupled receptor 1	2.091	-0.918
Gpr15	NM_001162955	G protein-coupled receptor 15	1.428	-0.655
Gpr152	NM_206973	G protein-coupled receptor 152	-1.071	-0.538
Gpr176	NM_201367	G protein-coupled receptor 176	-1.479	-0.331
Gpr182	NM_007412	G protein-coupled receptor 182	-1.243	0.808
Gpr52	NM_001146330	G protein-coupled receptor 52	-1.468	1.789
Gpx2-ps1	NR_033563	glutathione peroxidase 2, pseudogene 1	-1.479	-0.331
Grasp	NM_019518	GRP1 (general receptor for phosphoinositides 1)-associated scaffold protein	-1.397	-0.068
Grhl1	NM_001161406	grainyhead like transcription factor 1	-3.117	-0.430
Grin3b	NM_130455	glutamate receptor, ionotropic, NMDA3B	-1.079	-0.238
Gsdmcl-ps	NR_029414	gasdermin C-like, pseudogene	2.322	0.664
Gsg1	NM_001080552	germ cell associated 1	1.133	0.713
Gstm4	NM_001160411	glutathione S-transferase, mu 4	1.055	1.926
Gucy2f	NM_001007576	guanylate cyclase 2f	-2.034	-0.430
Gvin1	NM_001039160	GTPase, very large interferon inducible 1	-1.073	9.145
H2-Q7	NM_001198560	histocompatibility 2, Q region locus 7	2.491	1.981
H2-T9	NM_010399	histocompatibility 2, T region locus 9	1.158	3.767
H2afy2	NM_207000	H2A histone family, member Y2	-1.348	1.350
Hal	NM_010401	histidine ammonia lyase	-1.183	7.875
Hap1	NM_010404	huntingtin-associated protein 1	-1.487	2.090
Hapln3	NM_178255	hyaluronan and proteoglycan link protein 3	1.428	-0.655
Haus8	NM_001163042	4HAUS augmin-like complex, subunit 8	1.328	2.454
Hba-a1	NM_008218	hemoglobin alpha, adult chain 1	6.398	3.367
Hba-a2	NM_001083955	hemoglobin alpha, adult chain 2	11.868	4.656
Hbb-b1	NM_001278161	hemoglobin, beta adult major chain	7.328	4.268
Hbb-b2	NM_016956	hemoglobin, beta adult minor chain	7.048	0.149
Hbb-bt	NM_008220	hemoglobin, beta adult t chain	7.048	0.149
Hcn3	NM_008227	hyperpolarization-activated, cyclic nucleotide-gated K+ 3	-2.202	-0.330
Hcst	NM_011827	hematopoietic cell signal transducer	1.047	3.229
Hddc2	NM_027168	HD domain containing 2	1.079	1.734
Hddc3	NM_026812	HD domain containing 3	1.017	2.295
Hgf	NM_001289458	hepatocyte growth factor	1.567	3.719
Hic1	NM_001098203	hypermethylated in cancer 1	-1.163	0.893
Hid1	NM_001346774	HID1 domain containing	1.171	-0.780
Hist1h1b	NM_020034	histone cluster 1, H1b	1.056	2.743
Hist1h1c	NM_015786	histone cluster 1, H1c	1.115	6.451
Hist1h1d	NM_145713	histone cluster 1, H1d	1.049	4.610
Hist1h2ah	NM_175659	histone cluster 1, H2ah	1.336	1.905
Hist1h2an	NM_178184	histone cluster 1, H2an	1.626	1.863
Hist1h2ao	NM_001177544	histone cluster 1, H2ao	1.069	3.900
Hist1h2ap	NM_178185	histone cluster 1, H2ap	-1.434	1.917
Hist1h2bh	NM_178197	histone cluster 1, H2bh	1.842	1.341
Hist1h3b	NM_178203	histone cluster 1, H3b	1.000	2.182
Hist1h4j	NM_178210	histone cluster 1, H4j	1.637	1.043
Hist2h2ab	NM_178213	histone cluster 2, H2ab	-1.280	0.518
Hist2h3c1	NM_178216	histone cluster 2, H3c1	1.528	2.647
Hnf1a	NM_009327	HNF1 homeobox A	1.194	-0.070
Hnf4a	NM_001312906	hepatic nuclear factor 4, alpha	1.089	-0.433
Hoxa5	NM_010453	homeobox A5	5.783	-0.781

Hoxb3	NM_001079869	homeobox B3	-1.010	1.492
Hrh4	NM_153087	histamine receptor H4	1.089	-0.433
Hsd17b1	NM_010475	hydroxysteroid (17-beta) dehydrogenase 1	-2.284	-0.916
Hsf3	NM_001310754	heat shock transcription factor 3	-1.091	0.570
Hsf5	NM_001045527	heat shock transcription factor family member 5	1.428	-0.655
Hspa12b	NM_028306	heat shock protein 12B	-1.761	1.410
Htr5b	NM_010483	5-hydroxytryptamine (serotonin) receptor 5B	1.171	-0.780
Hyal5	NM_028957	hyaluronoglucosaminidase 5	1.295	0.459
Hyal6	NM_028920	hyaluronoglucosaminidase 6	1.278	-0.333
Id1	NM_001355113	inhibitor of DNA binding 1	1.039	3.300
Ido1	NM_001293690	indoleamine 2,3-dioxygenase 1	-2.284	-0.916
Ido2	NM_145949	indoleamine 2,3-dioxygenase 2	-5.666	-0.916
Ifi27	NM_026790	interferon, alpha-inducible protein 27	1.023	5.079
Ifit1bl2	NM_053217	interferon induced protein with tetratricopeptide repeats 1B like 2	-2.949	-0.537
Ifitm10	NM_001347541	interferon induced transmembrane protein 10	-1.079	-0.238
Igsf3	NM_207205	immunoglobulin superfamily, member 3	1.031	2.148
Igsf9b	NM_001033323	immunoglobulin superfamily, member 9B	-2.237	0.465
Il1rl2	NM_001356478	interleukin 1 receptor-like 2	2.756	-0.540
Il23r	NM_144548	interleukin 23 receptor	-1.627	-0.653
Il4	NM_021283	interleukin 4	1.855	0.007
Ildr2	NM_001164528	immunoglobulin-like domain containing receptor 2	-5.885	-0.778
Impg2	NM_174876	interphotoreceptor matrix proteoglycan 2	-1.202	1.256
Ino80dos	NR_045914	INO80 complex subunit D, opposite strand	-1.195	1.350
Inpp5j	NM_172439	inositol polyphosphate 5-phosphatase J	6.567	2.638
Insl3	NM_013564	insulin-like 3	-7.965	0.855
Insm2	NM_020287	insulinoma-associated 2	-1.479	-0.331
Iqsec3	NM_001033354	IQ motif and Sec7 domain 3	-1.737	0.809
Isg15	NM_015783	ISG15 ubiquitin-like modifier	1.039	0.803
Islr2	NM_001161535	immunoglobulin superfamily containing leucine-rich repeat 2	-3.117	-0.430
Itgae	NM_008399	integrin alpha E, epithelial-associated	-1.141	1.493
Itih5	NM_172471	inter-alpha (globulin) inhibitor H5	-1.548	0.852
Itm2a	NM_008409	integral membrane protein 2A	2.289	-0.154
Jph3	NM_020605	junctophilin 3	-1.220	0.084
Kazn	NM_001109684	kazrin, periplakin interacting protein	1.595	-0.153
Kcnc1	NM_001112739	potassium voltage gated channel, Shaw-related subfamily, member 1	-1.247	-0.150
Kcnj1	NM_001168354	potassium inwardly-rectifying channel, subfamily J, member 1	-2.034	-0.430
Kcnj13	NM_001110227	potassium inwardly-rectifying channel, subfamily J, member 13	1.800	1.311
Kcnj5	NM_010605	potassium inwardly-rectifying channel, subfamily J, member 5	-2.047	1.352
Kcnmb1	NM_031169	potassium large conductance calcium-activated channel, subfamily M, beta member 1	-1.458	0.222
Kcnmb3	NM_001195074	potassium large conductance calcium-activated channel, subfamily M, beta member 3	-1.647	-0.237
Kcnn1	NM_032397	potassium intermediate/small conductance calcium-activated channel, subfamily N, member 1	3.333	-0.154
Kcnt1	NM_001145403	potassium channel, subfamily T, member 1	-1.056	-0.917
Khdc3	NM_001311106	KH domain containing 3, subcortical maternal complex member	1.162	1.250
Kif14	NM_001081258	kinesin family member 14	1.014	1.044
Kif17	NM_001190978	kinesin family member 17	5.565	-0.919
Kif4	NM_008446	kinesin family member 4	1.472	1.457
Kirrel	NM_001170985	kirre like nephrin family adhesion molecule 1	1.295	0.459
Kiss1r	NM_053244	KISS1 receptor	1.194	-0.070
Klc3	NM_001286038	kinesin light chain 3	-2.284	-0.916
Klf11	NM_178357	Kruppel-like factor 11	1.447	3.631
Klf5	NM_009769	Kruppel-like factor 5	1.171	-0.780
Klhl14	NM_001081403	kelch-like 14	-1.063	3.196
Klk13	NM_001039042	kallikrein related-peptidase 13	-1.071	-0.538
Klk4	NM_019928	kallikrein related-peptidase 4 (prostase, enamel matrix, prostate)	2.348	-0.781
Klrd1	NM_010654	killer cell lectin-like receptor, subfamily D, member 1	2.034	0.458
Krt8	NM_031170	keratin 8	-1.091	0.570
LOC106740	NR_027905	uncharacterized LOC106740	-1.147	1.319
Lag3	NM_008479	lymphocyte-activation gene 3	1.202	0.403
Lamb1	NM_008482	laminin B1	-2.057	0.011
Lamb2	NM_008483	laminin, beta 2	1.062	3.212
Lbx2	NM_010692	ladybird homeobox 2	1.428	-0.655
Lepr	NM_001122899	leptin receptor	-1.280	0.518
Lhfp	NM_175386	lipoma HMGIC fusion partner	-2.468	0.288
Liph	NM_001083894	lipase, member H	1.773	0.282
Lnx1	NM_001159577	ligand of numb-protein X 1	-2.506	0.936
Lox	NM_001286181	lysyl oxidase	-1.289	-0.431
Lrrc19	NM_001356281	leucine rich repeat containing 19	1.171	-0.780
Lrrc27	NM_001143755	leucine rich repeat containing 27	3.447	-0.072
Lta	NM_010735	lymphotoxin A	-1.498	0.464
Ltk	NM_008523	leukocyte tyrosine kinase	-1.458	0.222
Ly6c1	NM_001252055	lymphocyte antigen 6 complex, locus C1	1.194	-0.070
Ly6c2	NM_001099217	lymphocyte antigen 6 complex, locus C2	-1.479	-0.331
Lyrm7	NM_029327	LYR motif containing 7	-1.458	0.222
Lyve1	NM_053247	lymphatic vessel endothelial hyaluronan receptor 1	-1.306	4.445
Maged2	NM_001199246	melanoma antigen, family D, 2	1.026	2.199
Mak	NM_001145802	male germ cell-associated kinase	-5.666	-0.916
Mamdc4	NM_001081199	MAM domain containing 4	2.008	0.712
Mansc4	NM_001034903	MANSC domain containing 4	1.445	-0.240
Masp2	NM_001003893	mannan-binding lectin serine peptidase 2	-1.158	1.012
Mboat1	NM_153546	membrane bound O-acyltransferase domain containing 1	1.450	2.058
Mcm6	NM_001313695	minichromosome maintenance complex component 6	1.042	4.138
Mcoln3	NM_134160	mucolipin 3	-1.344	0.155
Mctp1	NM_030174	multiple C2 domains, transmembrane 1	1.542	4.059

Mefv	NM_001161790	Mediterranean fever	-1.842	0.464
Melk	NM_010790	maternal embryonic leucine zipper kinase	1.463	0.566
Meox1	NM_010791	mesenchyme homeobox 1	1.428	-0.655
Met	NM_008591	met proto-oncogene	1.044	-0.153
Mfrp	NM_001190314	membrane frizzled-related protein	1.089	-0.433
Mgll	NM_001166249	monoglyceride lipase	3.902	1.585
Mgmt	NM_008598	O-6-methylguanine-DNA methyltransferase	1.020	1.539
Mid1	NM_001290504	midline 1	-1.145	2.440
Mkx	NM_177595	mohawk homeobox	7.207	0.280
Mmp12	NM_001320076	matrix metallopeptidase 12	-1.369	-0.779
Mmp14	NM_008608	matrix metallopeptidase 14 (membrane-inserted)	-1.647	-0.237
Mmp24	NM_010808	matrix metallopeptidase 24	1.005	1.151
Mmp25	NM_001033339	matrix metallopeptidase 25	2.528	1.535
Mmp8	NM_008611	matrix metallopeptidase 8	-1.188	0.463
Mmrn2	NM_153127	multimerin 2	5.783	-0.781
Mogat1	NM_026713	monoacylglycerol O-acyltransferase 1	1.127	1.430
Morn4	NM_198108	MORN repeat containing 4	-1.056	-0.917
Mrgpra4	NM_153524	MAS-related GPR, member A4	1.017	0.082
Mrgprx1	NM_207540	MAS-related GPR, member X1	-5.666	-0.916
Mroh2a	NM_001177364	maestro heat-like repeat family member 2A	2.756	-0.540
Ms4a7	NM_001025610	membrane-spanning 4-domains, subfamily A, member 7	-2.760	-0.653
Msln	NM_001356286	mesothelin	1.278	-0.333
Msrb3	NM_177092	methionine sulfoxide reductase B3	1.171	-0.780
Mt2	NM_008630	metallothionein 2	-1.955	0.765
Mtcp1	NM_001039373	mature T cell proliferation 1	-1.069	1.995
Mtfp1	NM_026443	mitochondrial fission process 1	1.533	0.802
Mtfr2	NM_027930	mitochondrial fission regulator 2	2.566	-0.655
Mtss1l	NM_001310591	metastasis suppressor 1-like	-1.877	0.223
Mtus2	NM_029920	microtubule associated tumor suppressor candidate 2	1.739	1.114
Mup19	NM_001135127	major urinary protein 19	8.042	1.002
Mup21	NM_001009550	major urinary protein 21	-1.056	-0.917
Mustn1	NM_181390	musculoskeletal, embryonic nuclear protein 1	1.044	-0.153
Mutyh	NM_001159581	mutY DNA glycosylase	1.209	1.043
Mybpc3	NM_008653	myosin binding protein C, cardiac	-2.057	0.011
Myh6	NM_001164171	myosin, heavy polypeptide 6, cardiac muscle, alpha	2.091	-0.918
Myh7	NM_080728	myosin, heavy polypeptide 7, cardiac muscle, beta	2.184	2.749
Mylk	NM_139300	myosin, light polypeptide kinase	4.091	1.751
Mylk3	NM_001297612	myosin light chain kinase 3	1.952	0.402
Myo5b	NM_008661	myosin VB	-1.670	0.764
Myzap	NM_001033208	myocardial zonula adherens protein	-1.546	1.673
Naaladl1	NM_001009546	N-acetylated alpha-linked acidic dipeptidase-like 1	6.770	-0.072
Nanog	NM_001289828	Nanog homeobox	-1.219	1.049
Nap1l5	NM_021432	nucleosome assembly protein 1-like 5	-1.057	1.598
Nat14	NM_201355	N-acetyltransferase 14	-1.071	-0.538
Ncapg	NM_019438	non-SMC condensin I complex, subunit G	1.607	1.311
Ndufaf6	NM_001085493	NADH dehydrogenase (ubiquinone) complex I, assembly factor 6	1.150	1.006
Ndufb2	NM_026612	NADH dehydrogenase (ubiquinone) 1 beta subcomplex, 2	1.272	2.561
Neto2	NM_001081324	neuropilin (NRP) and tolloid (TLL)-like 2	-1.842	0.464
Neurod4	NM_001329489	neurogenic differentiation 4	-1.454	1.697
Ngp	NM_008694	neutrophilic granule protein	-6.652	-0.236
Nhsl1	NM_001163592	NHS-like 1	-1.071	-0.538
Nkapl	NM_025719	NFKB activating protein-like	5.565	-0.919
Nlrp4f	NM_175290	NLR family, pyrin domain containing 4F	2.923	-0.434
Nmral1	NM_001290761	NmrA-like family domain containing 1	1.115	2.148
Nphp1	NM_001291012	nephronophthisis 1 (juvenile) homolog (human)	1.365	1.925
Nptxr	NM_030689	neuronal pentraxin receptor	-5.666	-0.916
Nr1d1	NM_145434	nuclear receptor subfamily 1, group D, member 1	-1.633	4.113
Nr2f2	NM_009697	nuclear receptor subfamily 2, group F, member 2	-1.055	1.519
Nr4a2	NM_001139509	nuclear receptor subfamily 4, group A, member 2	-1.288	1.409
Nrap	NM_001286552	nebulin-related anchoring protein	1.230	1.756
Nrg2	NM_001167891	neuregulin 2	-1.201	0.286
Nsg1	NM_010942	neuron specific gene family member 1	-1.280	0.518
Ntmt1	NM_001356433	N-terminal Xaa-Pro-Lys N-methyltransferase 1	1.074	2.427
Ntn3	NM_010947	netrin 3	-1.089	0.407
Nubpl	NM_029760	nucleotide binding protein-like	1.048	1.985
Nudt12	NM_026497	nudix (nucleoside diphosphate linked moiety X)-type motif 12	1.295	0.459
Nxpe2	NM_030069	neurexophilin and PC-esterase domain family, member 2	-1.220	0.084
Nyap1	NM_001347505	neuronal tyrosine-phosphorylated phosphoinositide 3-kinase adaptor 1	-1.260	0.670
Olfml2b	NM_177068	olfactomedin-like 2B	2.731	0.150
Olfr164	NM_146451	olfactory receptor 164	-1.505	0.974
Olfr173	NM_147000	olfactory receptor 173	2.348	-0.781
Oosp2	NM_001037634	oocyte secreted protein 2	-1.056	-0.917
Ophn1	NM_001313754	oligophrenin 1	-1.627	-0.653023
Orc1	NM_001014425	origin recognition complex, subunit 1	1.104	0.344252
Osbpl6	NM_001290733	oxysterol binding protein-like 6	-1.047	2.192468
Pabpc1l	NM_001114079	poly(A) binding protein, cytoplasmic 1-like	-1.056	-0.916557
Pacsin3	NM_001289677	protein kinase C and casein kinase substrate in neurons 3	1.855	0.006901
Panct2	NR_131964	pluripotency-associated noncoding transcript 2	-1.134	1.787863
Paqr5	NM_028748	progestin and adipoQ receptor family member V	-3.267	-0.329881
Paqr8	NM_001355122	progestin and adipoQ receptor family member VIII	-1.028	0.933337
Pard6b	NM_021409	par-6 family cell polarity regulator beta	-1.458	0.222414
Pbk	NM_023209	PDZ binding kinase	1.104	0.344252
Pbld2	NM_026085	phenazine biosynthesis-like protein domain containing 2	2.348	-0.780762

Pcbd1	NM_025273	pterin 4 alpha carbinolamine dehydratase/dimerization cofactor of hepatocyte nuclear factor 1 alpha (TCF1) 1	2.153	-0.240841
Pcdh1	NM_029357	protocadherin 1	1.674	0.218224
Pcdhac2	NM_001003672	protocadherin alpha subfamily C, 2	-2.760	-0.652535
Pcdhb16	NM_053141	protocadherin beta 16	1.278	-0.333247
Pcdhga6	NM_033589	protocadherin gamma subfamily A, 6	-1.585	0.518551
Pcdhga8	NM_033591	protocadherin gamma subfamily A, 8	-1.123	2.381819
Pcgf2	NM_001163307	polycomb group ring finger 2	-1.089	0.406607
Pcsk4	NM_008793	proprotein convertase subtilisin/kexin type 4	-1.289	-0.430623
Pcsk9	NM_153565	proprotein convertase subtilisin/kexin type 9	2.153	-0.240841
Pcyt1b	NM_177546	phosphate cytidylyltransferase 1, choline, beta isoform	-1.976	0.287240
Pdk4	NM_013743	pyruvate dehydrogenase kinase, isoenzyme 4	9.394	2.253892
Peg10	NM_001040611	paternally expressed 10	1.445	-0.240363
Perm1	NM_172417	PPARGC1 and ESRR induced regulator, muscle 1	-1.585	0.518551
Pex11a	NM_011068	peroxisomal biogenesis factor 11 alpha	1.692	1.370829
Pfn4	NM_028376	profilin family, member 4	-2.284	-0.916065
Pgf	NM_001271705	placental growth factor	5.972	-0.655947
Pglyrp1	NM_009402	peptidoglycan recognition protein 1	1.305	3.018613
Phex	NM_011077	phosphate regulating endopeptidase homolog, X-linked	1.835	-0.433038
Piezo2	NM_001039485	piezo-type mechanosensitive ion channel component 2	-2.760	-0.652535
Pigr	NM_011082	polymeric immunoglobulin receptor	-1.038	1.975049
Pira1	NM_011087	paired-Ig-like receptor A1	1.455	5.640761
Pitpnm2os1	NR_045369	phosphatidylinositol transfer protein, membrane-associated 2, opposite strand 1	-1.056	-0.916557
Pkhd1l1	NM_138674	polycystic kidney and hepatic disease 1-like 1	1.361	0.282604
Pla2g3	NM_172791	phospholipase A2, group III	7.207	0.280291
Pla2r1	NM_008867	phospholipase A2 receptor 1	1.428	-0.654973
Plagl1	NM_009538	pleiomorphic adenoma gene-like 1	-2.284	-0.916065
Platr26	NM_201366	pluripotency associated transcript 26	1.646	-0.539725
Plet1	NM_029639	placenta expressed transcript 1	1.428	-0.654973
Plin2	NM_007408	perilipin 2	1.572	7.557100
Plvap	NM_032398	plasmalemma vesicle associated protein	1.646	-0.539725
Pmel	NM_021882	premelanosome protein	1.044	-0.152647
Pnpla1	NM_001034885	patatin-like phospholipase domain containing 1	-1.202	1.256299
Pnpla3	NM_054088	patatin-like phospholipase domain containing 3	2.003	-0.333727
Pon1	NM_011134	paraoxonase 1	-2.284	-0.916065
Postn	NM_001198765	periostin, osteoblast specific factor	1.418	2.411712
Pparg	NM_001127330	peroxisome proliferator activated receptor gamma	-1.087	0.221948
Ppic	NM_008908	peptidylprolyl isomerase C	2.091	-0.918034
Ppm1j	NM_027982	protein phosphatase 1J	-1.307	0.347944
Ppp1r26	NM_001005420	protein phosphatase 1, regulatory subunit 26	1.143	1.342511
Ppp1r32	NM_133689	protein phosphatase 1, regulatory subunit 32	-2.034	-0.430140
Pramel5	NM_001085418	preferentially expressed antigen in melanoma like 5	-1.627	-0.653023
Prc1	NM_001285997	protein regulator of cytokinesis 1	1.093	2.294520
Prdm1	NM_007548	PR domain containing 1, with ZNF domain	-1.185	2.509293
Prdm16	NM_001177995	PR domain containing 16	-3.403	-0.236534
Prickle4	NM_001290337	prickle planar cell polarity protein 4	-1.056	-0.916557
Prkag3	NM_153744	protein kinase, AMP-activated, gamma 3 non-catatlytic subunit	1.171	-0.780272
Prob1	NM_001270646	proline rich basic protein 1	-1.709	1.957897
Proser2	NM_001159657	proline and serine rich 2	1.674	0.218224
Prrg1	NM_001164275	proline rich Gla (G-carboxyglutamic acid) 1	-2.488	-0.149312
Prss23	NM_029614	protease, serine 23	-2.760	-0.652535
Prss30	NM_013921	protease, serine 30	-1.056	-0.916557
Prss35	NM_178738	protease, serine 35	-1.099	2.013879
Prtg	NM_175485	protogenin	-1.289	-0.430623
Prx	NM_019412	periaxin	1.463	0.565801
Psmb11	NM_175204	proteasome (prosome, macropain) subunit, beta type, 11	2.091	-0.918034
Psmc3ip	NM_008949	proteasome (prosome, macropain) 26S subunit, ATPase 3, interacting protein	-1.289	-0.430623
Ptgdr2	NM_009962	prostaglandin D2 receptor 2	1.194	-0.070409
Ptk6	NM_001356304	PTK6 protein tyrosine kinase 6	-1.071	-0.537785
Ptprd	NM_001014288	protein tyrosine phosphatase, receptor type, D	-1.535	1.573551
Ptpru	NM_001083119	protein tyrosine phosphatase, receptor type, U	-1.380	0.893358
R74862	NM_133790	expressed sequence R74862	-1.172	1.742795
Rab20	NM_011227	RAB20, member RAS oncogene family	-1.257	1.648200
Rab30	NM_029494	RAB30, member RAS oncogene family	-1.002	3.228400
Rab34	NM_001159482	RAB34, member RAS oncogene family	-1.444	0.934214
Rab36	NM_029781	RAB36, member RAS oncogene family	1.089	-0.432555
Rab37	NM_001163753	RAB37, member RAS oncogene family	1.006	2.427837
Rab3b	NM_023537	RAB3B, member RAS oncogene family	-2.715	0.465252
Rab40b	NM_139147	Rab40B, member RAS oncogene family	5.783	-0.781251
Rab44	NM_001002786	RAB44, member RAS oncogene family	1.085	4.311332
Rab6b	NM_173781	RAB6B, member RAS oncogene family	-1.479	-0.330843
Rad51	NM_011234	RAD51 recombinase	1.407	0.887200
Rad54b	NM_001039556	RAD54 homolog B (S. cerevisiae)	-1.079	-0.237971
Radil	NM_001289588	Ras association and DIL domains	-5.666	-0.915573
Ramp1	NM_001168392	receptor (calcitonin) activity modifying protein 1	-1.463	1.319860
Rbfox3	NM_001024931	RNA binding protein, fox-1 homolog (C. elegans) 3	2.149	2.846396
Rcor2	NM_001320554	REST corepressor 2	1.141	0.151793
Rdh5	NM_134006	retinol dehydrogenase 5	1.646	-0.539725
Rdh7	NM_001150749	retinol dehydrogenase 7	1.171	-0.780272
Rec114	NM_028598	REC114 meiotic recombination protein	1.595	-0.153123
Reep1	NM_178608	receptor accessory protein 1	-1.011	0.669868
Reep2	NM_001204914	receptor accessory protein 2	-1.188	0.463420
Rell2	NM_153793	RELT-like 2	-1.289	-0.430623
Rem1	NM_009047	rad and gem related GTP binding protein 1	-1.141	1.492724

Retnlg	NM_181596	resistin like gamma	1.427	2.112074
Rfc4	NM_145480	replication factor C (activator 1) 4	1.316	1.964864
Rftn2	NM_001356287	raftlin family member 2	-1.173	3.055008
Rgcc	NM_025427	regulator of cell cycle	1.639	0.458847
Rgs13	NM_153171	regulator of G-protein signaling 13	-1.406	0.407067
Rgs7bp	NM_029879	regulator of G-protein signalling 7 binding protein	2.091	-0.918034
Rhbdd3	NM_001290491	rhomboid domain containing 3	1.031	2.399218
Rhebl1	NM_026967	Ras homolog enriched in brain like 1	1.014	1.043833
Rhobtb3	NM_028493	Rho-related BTB domain containing 3	-1.056	-0.916557
Rimbp3	NM_001033338	RIMS binding protein 3	-1.056	-0.916557
Rln3	NM_173184	relaxin 3	-1.056	-0.916557
Rmi2	NM_001033278	RecQ mediated genome instability 2	1.150	1.006123
Rmrp	NR_001460	RNA component of mitochondrial RNAase P	1.120	8.192853
Rnaset2b	NM_026611	ribonuclease T2B	-1.110	3.906453
Rnf148	NM_027754	ring finger protein 148	-2.541	-0.778313
Rnf180	NM_027934	ring finger protein 180	-1.011	0.669868
Rnf39	NM_001099632	ring finger protein 39	-1.220	0.084442
Rnf43	NM_172448	ring finger protein 43	-1.024	2.928075
Ropn1l	NM_145852	ropporin 1-like	1.194	-0.070409
Ror1	NM_001312690	receptor tyrosine kinase-like orphan receptor 1	-1.247	-0.150266
Rpl14-ps1	NR_110499	ribosomal protein L14, pseudogene 1	1.784	1.562650
Rpl22l1	NM_001347226	ribosomal protein L22 like 1	1.934	4.613764
Rpl26	NM_009080	ribosomal protein L26	1.679	3.677328
Rpl39	NM_026055	ribosomal protein L39	1.060	7.657849
Rprl3	NR_024198	ribonuclease P RNA-like 3	-1.330	8.980465
Rps15a-ps4	NR_036572	ribosomal protein S15A, pseudogene 4	1.309	3.152171
Rragb	NM_001004154	Ras-related GTP binding B	-1.158	1.011769
Rsad2	NM_021384	radical S-adenosyl methionine domain containing 2	1.454	0.081154
Rsph3b	NM_001083945	radial spoke 3B homolog (Chlamydomonas)	-1.028	1.787472
Rwdd2a	NM_001145968	RWD domain containing 2A	1.175	0.887639
Rwdd3	NM_025637	RWD domain containing 3	-1.092	0.717397
S100a5	NM_011312	S100 calcium binding protein A5	1.428	-0.654973
S100a9	NM_001281852	S100 calcium binding protein A9 (calgranulin B)	-2.897	2.417844
S1pr3	NM_010101	sphingosine-1-phosphate receptor 3	-1.793	1.548179
Saa1	NM_001357493	serum amyloid A 1	1.020	1.538851
Sag	NM_009118	S-antigen, retina and pineal gland (arrestin)	-1.670	0.764314
Sall2	NM_001244916	spalt like transcription factor 2	2.003	-0.333727
Samd4	NM_001037221	sterile alpha motif domain containing 4	-2.284	-0.916065
Sapcd2	NM_001081085	suppressor APC domain containing 2	1.171	-0.780272
Scarna6	NR_028519	small Cajal body-specific RNA 6	2.371	1.586809
Sccpdh	NM_178653	saccharopine dehydrogenase (putative)	1.133	2.041290
Scn3b	NM_001083917	sodium channel, voltage-gated, type III, beta	-1.674	1.087055
Scn4a	NM_133199	sodium channel, voltage-gated, type IV, alpha	-1.324	3.762057
Scnn1a	NM_011324	sodium channel, nonvoltage-gated 1 alpha	-2.284	-0.916065
Sdc2	NM_008304	syndecan 2	2.091	-0.918034
Sdk1	NM_177879	sidekick cell adhesion molecule 1	1.141	0.151793
Sdsl	NM_133902	serine dehydratase-like	1.322	0.665374
Sec16b	NM_001159986	SEC16 homolog B (S. cerevisiae)	1.881	1.215626
Sema3g	NM_001025379	sema domain, immunoglobulin domain (Ig), short basic domain, secreted, (semaphorin) 3G	1.017	0.081623
Sema4g	NM_011976	sema domain, immunoglobulin domain (Ig), transmembrane domain (TM) and short cytoplasmic domain, (semaphorin)	-1.737	0.808924
Serpina10	NM_001301404	serine (or cysteine) peptidase inhibitor, clade A (alpha-1 antiproteinase, antitrypsin), member 10	1.044	-0.152647
Serpinb6b	NM_011454	serine (or cysteine) peptidase inhibitor, clade B, member 6b	-1.087	0.221948
Serpinb7	NM_027548	serine (or cysteine) peptidase inhibitor, clade B, member 7	-1.056	-0.916557
Serpinf1	NM_011340	serine (or cysteine) peptidase inhibitor, clade F, member 1	-2.284	-0.916065
Setd4	NM_145482	SET domain containing 4	1.422	1.183493
Sez6l2	NM_001252566	seizure related 6 homolog like 2	-1.034	1.049023
Sh2b2	NM_001302938	SH2B adaptor protein 2	-1.048	3.435303
Sh2d1a	NM_001313688	SH2 domain containing 1A	1.428	-0.654973
Shcbp1	NM_011369	Shc SH2-domain binding protein 1	2.824	0.217295
Shf	NM_001013829	Src homology 2 domain containing F	-1.458	0.222414
Shisa2	NM_145463	shisa family member 2	6.140	-0.540695
Shisa7	NM_001290291	shisa family member 7	-3.024	0.223813
Siglecf	NM_001271019	sialic acid binding Ig-like lectin F	4.393	2.951597
Skida1	NM_028317	SKI/DACH domain containing 1	5.565	-0.918525
Slain1os	NR_045148	SLAIN motif family, member 1, opposite strand	-1.564	1.012639
Slc16a8	NM_020516	solute carrier family 16 (monocarboxylic acid transporters), member 8	2.348	-0.780762
Slc18a1	NM_153054	solute carrier family 18 (vesicular monoamine), member 1	2.348	-0.780762
Slc22a4	NM_001330304	solute carrier family 22 (organic cation transporter), member 4	1.460	0.343791
Slc25a18	NM_001081048	solute carrier family 25 (mitochondrial carrier), member 18	1.025	0.928519
Slc25a31	NM_178386	solute carrier family 25 (mitochondrial carrier; adenine nucleotide translocator), member 31	1.171	-0.780272
Slc25a47	NM_001012310	solute carrier family 25, member 47	-1.877	0.222881
Slc27a5	NM_009512	solute carrier family 27 (fatty acid transporter), member 5	1.835	-0.433038
Slc29a4	NM_146257	solute carrier family 29 (nucleoside transporters), member 4	-1.585	0.518551
Slc2a10	NM_130451	solute carrier family 2 (facilitated glucose transporter), member 10	1.445	-0.240363
Slc2a5	NM_019741	solute carrier family 2 (facilitated glucose transporter), member 5	2.153	-0.240841
Slc39a12	NM_001012305	solute carrier family 39 (zinc transporter), member 12	-1.627	-0.653023
Slc43a3	NM_021398	solute carrier family 43, member 3	1.059	1.184343
Slc46a2	NM_021053	solute carrier family 46, member 2	-1.056	-0.916557
Slc4a10	NM_001242378	solute carrier family 4, sodium bicarbonate cotransporter-like, member 10	-1.533	0.010676
Slc6a9	NM_001355175	solute carrier family 6 (neurotransmitter transporter, glycine), member 9	1.089	-0.432555
Slco1b2	NM_020495	solute carrier organic anion transporter family, member 1b2	-1.056	-0.916557
Slco4c1	NM_172658	solute carrier organic anion transporter family, member 4C1	5.783	-0.781251
Slfnl1	NM_177570	schlafen like 1	-1.056	-0.916557

Slit2	NM_001291227	slit guidance ligand 2	-2.949	-0.536813
Smim5	NM_183259	small integral membrane protein 5	-1.627	-0.653023
Snap91	NM_001277982	synaptosomal-associated protein 91	-2.541	-0.778313
Snhg10	NR_003145	small nucleolar RNA host gene 10	5.972	-0.655947
Snhg18	NR_038186	small nucleolar RNA host gene 18	-1.481	0.808480
Snora23	NR_033336	small nucleolar RNA, H/ACA box 23	3.209	-0.241319
Snord15a	NR_002172	small nucleolar RNA, C/D box 15A	1.428	-0.654973
Snord15b	NR_002173	small nucleolar RNA, C/D box 14B	-1.397	-0.067564
Sorbs2	NM_001205219	sorbin and SH3 domain containing 2	-2.155	0.407987
Sox4	NM_009238	SRY (sex determining region Y)-box 4	1.835	-0.433038
Sox5os3	NR_040519	SRY (sex determining region Y)-box 5, opposite strand 3	-1.470	1.086624
Sox6	NM_001025559	SRY (sex determining region Y)-box 6	-1.715	1.599678
Sp3os	NM_183265	trans-acting transcription factor 3, opposite strand	-1.873	1.225370
Spag6	NM_001001334	sperm associated antigen 6	-5.666	-0.915573
Spam1	NM_001079875	sperm adhesion molecule 1	1.835	-0.433038
Sparc	NM_001290817	secreted acidic cysteine rich glycoprotein	1.460	0.343791
Spata7	NM_001289572	spermatogenesis associated 7	-1.417	1.493543
Spc25	NM_001199123	SPC25, NDC80 kinetochore complex component, homolog (S. cerevisiae)	1.109	0.846021
Spp1	NM_001204201	secreted phosphoprotein 1	1.391	3.770646
Sptbn4	NM_001199234	spectrin beta, non-erythrocytic 4	1.278	-0.333247
Srd5a1	NM_175283	steroid 5 alpha-reductase 1	2.266	0.281679
Ssc4d	NM_001160366	scavenger receptor cysteine rich family, 4 domains	1.541	0.616221
Sstr4	NM_009219	somatostatin receptor 4	-1.178	0.621185
Stag3	NM_016964	stromal antigen 3	1.270	3.028127
Stard10	NM_019990	START domain containing 10	-1.041	2.032539
Stc1	NM_009285	stanniocalcin 1	1.428	-0.654973
Stc2	NM_011491	stanniocalcin 2	-1.369	-0.778803
Steap4	NM_054098	STEAP family member 4	-3.219	1.747918
Stil	NM_001304551	Scl/Tal1 interrupting locus	1.463	0.565801
Stmn1	NM_019641	stathmin 1	1.647	3.044369
Sult4a1	NM_001356515	sulfotransferase family 4A, member 1	2.153	-0.240841
Syde1	NM_027875	synapse defective 1, Rho GTPase, homolog 1 (C. elegans)	1.595	-0.153123
Syne1	NM_001079686	spectrin repeat containing, nuclear envelope 1	1.211	5.960392
Syt12	NM_134164	synaptotagmin XII	-2.541	-0.778313
Sytl1	NM_031393	synaptotagmin-like 1	1.124	2.095628
Sytl4	NM_001290717	synaptotagmin-like 4	1.646	-0.539725
Tagln3	NM_019754	transgelin 3	2.756	-0.540210
Tarm1	NM_177363	T cell-interacting, activating receptor on myeloid cells 1	-1.087	0.221948
Tat	NM_146214	tyrosine aminotransferase	-1.079	-0.237971
Tbx2	NM_009324	T-box 2	-1.056	-0.916557
Tc2n	NM_001082976	tandem C2 domains, nuclear	-1.071	-0.537785
Tcaf2	NM_146174	TRPM8 channel-associated factor 2	-1.220	0.084442
Tceal1	NM_001356367	transcription elongation factor A (SII)-like 1	2.091	-0.918034
Tcerg1l	NM_183289	transcription elongation regulator 1-like	1.646	-0.539725
Tcf23	NM_053085	transcription factor 23	1.751	0.757631
Tdrkh	NM_028307	tudor and KH domain containing protein	1.123	2.263104
Tead1	NM_001166584	TEA domain family member 1	-2.760	-0.652535
Teddm1a	NM_178244	transmembrane epididymal protein 1A	1.646	-0.539725
Teddm1b	NM_001008426	transmembrane epididymal protein 1B	-1.369	-0.778803
Tesc	NM_021344	tescalcin	1.055	0.665823
Tex11	NM_001167997	testis expressed gene 11	1.171	-0.780272
Tex15	NM_031374	testis expressed gene 15	-1.247	-0.150266
Tex22	NM_029381	testis expressed gene 22	2.923	-0.433520
Tgfb3	NM_009368	transforming growth factor, beta 3	1.194	-0.070409
Thbs1	NM_001313914	thrombospondin 1	1.438	7.101035
Themis	NM_001305663	thymocyte selection associated	1.126	1.687559
Ticrr	NM_029835	TOPBP1-interacting checkpoint and replication regulator	1.047	1.281488
Tigd3	NM_198634	tigger transposable element derived 3	1.017	0.081623
Tigit	NM_001146325	T cell immunoreceptor with Ig and ITIM domains	1.089	-0.432555
Tlr5	NM_016928	toll-like receptor 5	1.171	-0.780272
Tm6sf2	NM_001293795	transmembrane 6 superfamily member 2	1.445	-0.240363
Tmc4	NM_181820	transmembrane channel-like gene family 4	-1.097	1.379130
Tmem117	NM_178789	transmembrane protein 117	-1.627	-0.653023
Tmem14a	NM_001290679	transmembrane protein 14A	1.278	-0.333247
Tmem160	NM_026938	transmembrane protein 160	1.081	4.473179
Tmem17	NM_153596	transmembrane protein 17	-1.647	-0.237492
Tmem200b	NM_001201367	transmembrane protein 200B	1.089	-0.432555
Tmem205	NM_001253867	transmembrane protein 205	1.007	2.883393
Tmem216	NM_001277860	transmembrane protein 216	1.176	1.639419
Tmem221	NM_001100462	transmembrane protein 221	-1.071	-0.537785
Tmem229a	NM_177013	transmembrane protein 229A	2.011	2.738187
Tmem240	NM_001101506	transmembrane protein 240	-1.533	0.010676
Tmem254a	NM_025311	transmembrane protein 254a	6.426	-0.334687
Tmem254b	NM_001270495	transmembrane protein 254b	-8.305	1.161709
Tmem254c	NM_001270498	transmembrane protein 254c	9.018	1.898420
Tmem38a	NM_001357278	transmembrane protein 38A	-1.449	0.621637
Tmem81	NM_029025	transmembrane protein 81	1.056	2.742923
Tmem91	NM_001290497	transmembrane protein 91	-2.541	-0.778313
Tmem98	NM_029537	transmembrane protein 98	-1.056	-0.916557
Tmod4	NM_016712	tropomodulin 4	-1.020	0.807591
Tmsb15b1	NM_001081983	thymosin beta 15b1	1.428	-0.654973
Tnf	NM_001278601	tumor necrosis factor	-1.281	2.243155
Tnfrsf19	NM_001164155	tumor necrosis factor receptor superfamily, member 19	1.162	1.249548

Tnfsf14	NM_019418	tumor necrosis factor (ligand) superfamily, member 14	1.761	1.661904
Tnfsf18	NM_183391	tumor necrosis factor (ligand) superfamily, member 18	-1.289	-0.430623
Tnfsf9	NM_009404	tumor necrosis factor (ligand) superfamily, member 9	-1.071	-0.537785
Tnni3	NM_009406	troponin I, cardiac 3	-1.369	-0.778803
Tnnt3	NM_001163664	troponin T3, skeletal, fast	-1.079	-0.237971
Tnr	NM_022312	tenascin R	-1.139	1.572338
Top2a	NM_011623	topoisomerase (DNA) II alpha	1.311	4.504489
Tpbg	NM_001164792	trophoblast glycoprotein	-1.079	-0.237971
Tpx2	NM_001141975	TPX2, microtubule-associated	1.382	2.440062
Trim12a	NM_023835	tripartite motif-containing 12A	2.566	-0.655460
Trim36	NM_001170855	tripartite motif-containing 36	1.153	1.538446
Trim43b	NM_001170884	tripartite motif-containing 43B	-1.056	-0.916557
Trim6	NM_001013616	tripartite motif-containing 6	5.565	-0.918525
Trim72	NM_001079932	tripartite motif-containing 72	1.475	2.002644
Troap	NM_001162506	trophinin associated protein	1.454	0.081154
Trp53cor1	NR_036469	tumor protein p53 pathway corepressor 1	1.044	-0.152647
Trpc2	NM_001109897	transient receptor potential cation channel, subfamily C, member 2	6.961	0.079277
Trpm3	NM_001035239	transient receptor potential cation channel, subfamily M, member 3	2.348	-0.780762
Trpt1	NM_153597	tRNA phosphotransferase 1	-1.657	0.084912
Tspan33	NM_001301407	tetraspanin 33	3.073	-0.334207
Tspo	NM_009775	translocator protein	1.089	6.158546
Tssk4	NM_001253888	testis-specific serine kinase 4	-1.071	-0.537785
Tst	NM_009437	thiosulfate sulfurtransferase, mitochondrial	2.112	0.512632
Ttc39a	NM_001145948	tetratricopeptide repeat domain 39A	1.330	0.007372
Ttc9	NM_001033149	tetratricopeptide repeat domain 9	-2.284	-0.916065
Ttk	NM_001110265	Ttk protein kinase	1.276	0.802712
Ttll11	NM_028921	tubulin tyrosine ligase-like family, member 11	1.595	-0.153123
Ttll7	NM_001302957	tubulin tyrosine ligase-like family, member 7	-2.541	-0.778313
Ttyh1	NM_001001454	tweety family member 1	-1.289	-0.430623
Tub	NM_021885	tubby bipartite transcription factor	-1.141	2.562177
Tubb3	NM_023279	tubulin, beta 3 class III	1.133	0.713
Tube1	NM_028006	epsilon-tubulin 1	1.129	1.116
Ubap1l	NM_001111145	ubiquitin-associated protein 1-like	1.089	-0.433
Ube2c	NM_026785	ubiquitin-conjugating enzyme E2C	1.276	1.429
Ube4bos3	NR_131918	ubiquitination factor E4B, opposite strand 3	1.055	0.666
Ugt1a9	NM_201644	UDP glucuronosyltransferase 1 family, polypeptide A9	-6.392	-0.429
Ugt2b5	NM_009467	UDP glucuronosyltransferase 2 family, polypeptide B5	2.091	-0.918
Uox	NM_009474	urate oxidase	2.566	-0.655
Vangl2	NM_033509	VANGL planar cell polarity 2	1.569	0.151
Vmn2r-ps129	NR_033648	vomeronasal 2, receptor, pseudogene 129	-1.247	-0.150
Vmn2r26	NM_019917	vomeronasal 2, receptor 26	1.291	4.694
Vmn2r84	NM_001081448	vomeronasal 2, receptor 84	-2.637	0.408
Whrn	NM_001008791	whirlin	-1.021	3.332
Wipf3	NM_001167860	WAS/WASL interacting protein family, member 3	-1.481	0.808
Wnt16	NM_053116	wingless-type MMTV integration site family, member 16	-1.079	-0.238
Wnt8b	NM_011720	wingless-type MMTV integration site family, member 8B	-5.666	-0.916
Wtip	NM_207212	WT1-interacting protein	1.064	2.820
Wwc1	NM_170779	WW, C2 and coiled-coil domain containing 1	-1.369	-0.779
Xkrx	NM_183319	X-linked Kx blood group related, X-linked	1.461	3.490
Xlr3b	NM_001081643	X-linked lymphocyte-regulated 3B	-1.071	-0.538
Xlr4a	NM_001081642	X-linked lymphocyte-regulated 4A	-1.845	-0.537
Xlr4b	NM_001293676	X-linked lymphocyte-regulated 4B	-1.004	1.408
Xlr4c	NM_183094	X-linked lymphocyte-regulated 4C	-2.555	0.349
Xpnpep2	NM_001289729	X-prolyl aminopeptidase (aminopeptidase P) 2, membrane-bound	1.017	0.082
Xrra1	NM_001164258	X-ray radiation resistance associated 1	1.428	-0.655
Zbtb16	NM_001033324	zinc finger and BTB domain containing 16	1.365	5.059
Zdhhc2	NM_001357249	zinc finger, DHHC domain containing 2	1.176	2.131
Zfa-ps	NM_009540	zinc finger protein, autosomal, pseudogene	1.731	-0.071
Zfhx2os	NR_004444	zinc finger homeobox 2, opposite strand	-2.314	0.519
Zfp30	NM_013705	zinc finger protein 30	-1.170	0.763
Zfp473	NM_001289836	zinc finger protein 473	1.171	-0.780
Zfp493	NM_028402	zinc finger protein 493	-1.066	1.874
Zfp503	NM_145459	zinc finger protein 503	-1.600	0.718
Zfp57	NM_001013745	zinc finger protein 57	5.972	-0.656
Zfp612	NM_175480	zinc finger protein 612	-1.089	0.407
Zfp72	NM_001081680	zinc finger protein 72	-1.163	0.893
Zfp827	NM_001294279	zinc finger protein 827	-1.026	3.771
Zfp867	NM_178417	zinc finger protein 867	-1.149	2.888
Zfp938	NM_001105557	zinc finger protein 938	-1.156	2.106
Zfp940	NM_173738	zinc finger protein 940	-1.253	1.288
Zfp941	NM_001001180	zinc finger protein 941	2.153	-0.241
Zfr2	NM_001034895	zinc finger RNA binding protein 2	1.206	0.759
Zkscan16	NM_001099323	zinc finger with KRAB and SCAN domains 16	-1.071	-0.538
Znhit3	NM_001005223	zinc finger, HIT type 3	1.018	1.966

Table S2. Gene list using GO analysis in Figure 3A

Gene Symbol	Genbank accession	Description	Control / MCS	
			log2FC	log2CPM
1500011K16Rik	NM_175125	RIKEN cDNA 1500011K16 gene	1.206	3.336269
1700071M16Rik	NR_045444	RIKEN cDNA 1700071M16 gene	1.163	3.879029
3110056K07Rik	NR_045055	RIKEN cDNA 3110056K07 gene	-1.101	3.063772
5031425F14Rik	NR_015558	RIKEN cDNA 5031425F14 gene	1.173	3.118620
Acrbp	NM_001127340	proacrosin binding protein	-1.019	3.090596
Adgrg5	NM_001033468	adhesion G protein-coupled receptor G5	1.350	3.537420
Agbl1	NM_001199224	ATP/GTP binding protein-like 1	-1.278	3.154374
Akap5	NM_001101471	A kinase (PRKA) anchor protein 5	-1.457	3.000019
Arap3	NM_001205336	ArfGAP with RhoGAP domain, ankyrin repeat and PH domain 3	1.267	4.579340
Ass1	NM_007494	argininosuccinate synthetase 1	1.133	4.666438
Atg16l2	NM_001111111	autophagy related 16-like 2 (S. cerevisiae)	-1.010	3.161139
Atp5e	NM_025983	ATP synthase, H+ transporting, mitochondrial F1 complex, epsilon subunit	1.349	5.509464
Atp6v0c-ps2	NR_037854	ATPase, H+ transporting, lysosomal V0 subunit C, pseudogene 2	11.405	4.201016
C1rl	NM_181344	complement component 1, r subcomponent-like	1.386	3.808859
Cacna1i	NM_001044308	calcium channel, voltage-dependent, alpha 1I subunit	-1.042	6.867565
Ccr3	NM_009914	chemokine (C-C motif) receptor 3	4.668	4.170687
Ccs	NM_016892	copper chaperone for superoxide dismutase	1.173	3.313859
Cox7a2	NM_009945	cytochrome c oxidase subunit VIIa 2	1.082	4.756301
Crmp1	NM_001136058	collapsin response mediator protein 1	-1.230	3.064642
Cxcl13	NM_018866	chemokine (C-X-C motif) ligand 13	-2.047	8.084261
D130040H23Rik	NM_172491	RIKEN cDNA D130040H23 gene	-1.004	3.564427
Dbp	NM_016974	D site albumin promoter binding protein	-1.600	3.711966
Dtx1	NM_008052	deltex 1, E3 ubiquitin ligase	-1.487	5.210563
Dynlt1b	NM_009342	dynein light chain Tctex-type 1B	1.007	5.267176
Egr1	NM_007913	early growth response 1	-1.274	4.523850
Fabp7	NM_021272	fatty acid binding protein 7, brain	1.416	5.659176
Fcgr4	NM_144559	Fc receptor, IgG, low affinity IV	1.127	5.854742
Fcmr	NM_026976	Fc fragment of IgM receptor	-1.416	6.130246
Gm14308	NM_001099349	predicted gene 14308	-1.644	4.433517
Gm14430	NM_001100415	predicted gene 14430	5.725	3.653902
Gm17757	NR_040453	GTPase, very large interferon inducible 1 pseudogene	2.239	5.902816
Gm18853	NR_040456	GTPase, very large interferon inducible 1 pseudogene	-12.831	5.588670
Gm5424	NR_002687	argininosuccinate synthase pseudogene	-1.564	4.676945
Gm6644	NR_028277	Akr1b3 pseudogene	12.240	5.022312
Gvin1	NM_001039160	GTPase, very large interferon inducible 1	-1.073	9.144623
H2-T9	NM_010399	histocompatibility 2, T region locus 9	1.158	3.766972
Hal	NM_010401	histidine ammonia lyase	-1.183	7.875363
Hba-a1	NM_008218	hemoglobin alpha, adult chain 1	6.398	3.366547
Hba-a2	NM_001083955	hemoglobin alpha, adult chain 2	11.868	4.656449
Hbb-b1	NM_001278161	hemoglobin, beta adult major chain	7.328	4.268307
Hcst	NM_011827	hematopoietic cell signal transducer	1.047	3.228529
Hgf	NM_001289458	hepatocyte growth factor	1.567	3.718730
Hist1h1c	NM_015786	histone cluster 1, H1c	1.115	6.450653
Hist1h1d	NM_145713	histone cluster 1, H1d	1.049	4.609709
Hist1h2ao	NM_001177544	histone cluster 1, H2ao	1.069	3.900243
Id1	NM_001355113	inhibitor of DNA binding 1	1.039	3.299522
Ifi27	NM_026790	interferon, alpha-inducible protein 27	1.023	5.078674
Klf11	NM_178357	Kruppel-like factor 11	1.447	3.631074
Klhl14	NM_001081403	kelch-like 14	-1.063	3.195577
Lamb2	NM_008483	laminin, beta 2	1.062	3.212174
Lyve1	NM_053247	lymphatic vessel endothelial hyaluronan receptor 1	-1.306	4.444605
Mcm6	NM_001313695	minichromosome maintenance complex component 6	1.042	4.138040
Mctp1	NM_030174	multiple C2 domains, transmembrane 1	1.542	4.058785
Nr1d1	NM_145434	nuclear receptor subfamily 1, group D, member 1	-1.633	4.112793
Pglyrp1	NM_009402	peptidoglycan recognition protein 1	1.305	3.018613
Pira1	NM_011087	paired-Ig-like receptor A1	1.455	5.640761
Plin2	NM_007408	perilipin 2	1.572	7.557100
Rab30	NM_029494	RAB30, member RAS oncogene family	-1.002	3.228400
Rab44	NM_001002786	RAB44, member RAS oncogene family	1.085	4.311332
Rftn2	NM_001356287	raftlin family member 2	-1.173	3.055008
Rmrp	NR_001460	RNA component of mitochondrial RNAase P	1.120	8.192853
Rnaset2b	NM_026611	ribonuclease T2B	-1.110	3.906453
Rpl22l1	NM_001347226	ribosomal protein L22 like 1	1.934	4.613764
Rpl26	NM_009080	ribosomal protein L26	1.679	3.677328
Rpl39	NM_026055	ribosomal protein L39	1.060	7.657849
Rprl3	NR_024198	ribonuclease P RNA-like 3	-1.330	8.980465
Rps15a-ps4	NR_036572	ribosomal protein S15A, pseudogene 4	1.309	3.152171
Scn4a	NM_133199	sodium channel, voltage-gated, type IV, alpha	-1.324	3.762057
Sh2b2	NM_001302938	SH2B adaptor protein 2	-1.048	3.435303
Spp1	NM_001204201	secreted phosphoprotein 1	1.391	3.770646
Stag3	NM_016964	stromal antigen 3	1.270	3.028127
Stmn1	NM_019641	stathmin 1	1.647	3.044369
Syne1	NM_001079686	spectrin repeat containing, nuclear envelope 1	1.211	5.960392
Thbs1	NM_001313914	thrombospondin 1	1.438	7.101035
Tmem160	NM_026938	transmembrane protein 160	1.081	4.473179
Top2a	NM_011623	topoisomerase (DNA) II alpha	1.311	4.504489
Tspo	NM_009775	translocator protein	1.089	6.158546

Vmn2r26	NM_019917	vomeronasal 2, receptor 26	1.291	4.694216
Whrn	NM_001008791	whirlin	-1.021	3.331669
Xkrx	NM_183319	X-linked Kx blood group related, X-linked	1.461	3.490385
Zbtb16	NM_001033324	zinc finger and BTB domain containing 16	1.365	5.059115
Zfp827	NM_001294279	zinc finger protein 827	-1.026	3.770834

Table S3. Phagocytosis-related gene expression shown in Figure 5E

Gene Symbol	Genbank accession	Description	log2(ZT2/ZT14)	
			Control	MCS
Actb	NM_007393	actin, beta	0.071	0.115982
Actg1	NM_001313923	actin, gamma, cytoplasmic 1	0.228	0.012930
Atp6ap1	NM_018794	ATPase, H+ transporting, lysosomal accessory protein 1	0.128	0.025068
Atp6v0a1	NM_001243049	ATPase, H+ transporting, lysosomal V0 subunit A1	0.091	0.124081
Atp6v0a2	NM_011596	ATPase, H+ transporting, lysosomal V0 subunit A2	-0.201	0.216269
Atp6v0b	NM_033617	ATPase, H+ transporting, lysosomal V0 subunit B	-0.234	0.212740
Atp6v0c	NM_009729	ATPase, H+ transporting, lysosomal V0 subunit C	-0.053	-0.003881
Atp6v0d1	NM_013477	ATPase, H+ transporting, lysosomal V0 subunit D1	0.061	0.296234
Atp6v0e	NM_025272	ATPase, H+ transporting, lysosomal V0 subunit E	0.079	0.316161
Atp6v0e2	NM_001347164	ATPase, H+ transporting, lysosomal V0 subunit E2	-0.293	0.451371
Atp6v1a	NM_007508	ATPase, H+ transporting, lysosomal V1 subunit A	0.254	0.288856
Atp6v1b2	NM_007509	ATPase, H+ transporting, lysosomal V1 subunit B2	0.167	0.251854
Atp6v1c1	NM_025494	ATPase, H+ transporting, lysosomal V1 subunit C1	-0.105	-0.333098
Atp6v1c2	NM_001159632	ATPase, H+ transporting, lysosomal V1 subunit C2	-0.735	-0.919055
Atp6v1d	NM_023721	ATPase, H+ transporting, lysosomal V1 subunit D	-0.216	0.056981
Atp6v1e1	NM_007510	ATPase, H+ transporting, lysosomal V1 subunit E1	0.058	0.281697
Atp6v1f	NM_025381	ATPase, H+ transporting, lysosomal V1 subunit F	0.044	0.301869
Atp6v1g1	NM_024173	ATPase, H+ transporting, lysosomal V1 subunit G1	-0.002	0.392225
Atp6v1g2	NM_001347351	ATPase, H+ transporting, lysosomal V1 subunit G2	0.072	-1.435846
Atp6v1h	NM_001310442	ATPase, H+ transporting, lysosomal V1 subunit H	-0.036	0.393856
C1ra	NM_023143	complement component 1, r subcomponent A	0.019	0.784136
C1rb	NM_001113356	complement component 1, r subcomponent B	-0.250	-3.352015
C3	NM_009778	complement component 3	-0.032	0.000190
Calr	NM_007591	calreticulin	0.170	-0.224425
Canx	NM_001110499	calnexin	0.182	0.131213
Cd14	NM_009841	CD14 antigen	0.414	0.175229
Cd209a	NM_133238	CD209a antigen	0.539	-0.881988
Cd209b	NM_001037800	CD209b antigen	-0.447	-0.238992
Cd209c	NM_130903	CD209c antigen	0.213	-0.709337
Cd209d	NM_130904	CD209d antigen	0.072	-0.423098
Cd209g	NM_027343	CD209g antigen	-1.250	-1.182090
Cd36	NM_001159555	CD36 molecule	0.385	0.046878
Clec7a	NM_001309637	C-type lectin domain family 7, member a	-0.609	-0.429426
Colec12	NM_130449	collectin sub-family member 12	-0.008	-0.258131
Comp	NM_016685	cartilage oligomeric matrix protein	0.394	1.817910
Coro1a	NM_001301374	coronin, actin binding protein 1A	-0.088	0.584560
Ctsl	NM_009984	cathepsin L	1.025	-0.164970
Ctss	NM_001267695	cathepsin S	0.109	0.078287
Cyba	NM_001301284	cytochrome b-245, alpha polypeptide	0.060	0.490081
Cybb	NM_007807	cytochrome b-245, beta polypeptide	0.032	0.408418
Dync1h1	NM_030238	dynein cytoplasmic 1 heavy chain 1	-0.102	0.064359
Dync1i2	NM_001198872	dynein cytoplasmic 1 intermediate chain 2	0.081	-0.166915
Dync1li1	NM_146229	dynein cytoplasmic 1 light intermediate chain 1	-0.083	-0.162854
Dync1li2	NM_001013380	dynein, cytoplasmic 1 light intermediate chain 2	-0.325	0.076683
Dync2h1	NM_029851	dynein cytoplasmic 2 heavy chain 1	0.232	-0.502552
Eea1	NM_001001932	early endosome antigen 1	0.171	-0.460352
Fcgr1	NM_010186	Fc receptor, IgG, high affinity I	0.011	0.664105
Fcgr2b	NM_001077189	Fc receptor, IgG, low affinity IIb	-0.507	-0.032891
Fcgr3	NM_001356511	Fc receptor, IgG, low affinity III	0.132	0.021691
Fcgr4	NM_144559	Fc receptor, IgG, low affinity IV	0.230	0.979915
H2-Aa	NM_010378	histocompatibility 2, class II antigen A, alpha	0.019	-0.207204
H2-Ab1	NM_207105	histocompatibility 2, class II antigen A, beta 1	0.144	-0.430641
H2-Bl	NM_008199	histocompatibility 2, blastocyst	0.563	0.579750
H2-D1	NM_010380	histocompatibility 2, D region locus 1	0.063	0.877035
H2-DMa	NM_010386	histocompatibility 2, class II, locus DMa	0.102	0.062918
H2-DMb1	NM_010387	histocompatibility 2, class II, locus Mb1	-0.029	-0.541305
H2-DMb2	NM_010388	histocompatibility 2, class II, locus Mb2	-0.095	-0.131516
H2-Eb1	NM_010382	histocompatibility 2, class II antigen E beta	0.129	-0.052332
H2-Eb2	NM_001033978	histocompatibility 2, class II antigen E beta2	-0.665	0.509788
H2-K1	NM_001001892	histocompatibility 2, K1, K region	-0.004	0.450812
H2-L	NM_001267808	histocompatibility 2, D region locus L	0.620	-0.182090
H2-M3	NM_013819	histocompatibility 2, M region locus 3	0.124	1.021002
H2-Oa	NM_008206	histocompatibility 2, O region alpha locus	0.424	-0.589265
H2-Ob	NM_010389	histocompatibility 2, O region beta locus	-0.031	-0.134261
H2-Q1	NM_010390	histocompatibility 2, Q region locus 1	0.182	0.414013
H2-Q10	NM_010391	histocompatibility 2, Q region locus 10	-0.099	0.760577
H2-Q2	NM_010392	histocompatibility 2, Q region locus 2	0.353	0.596620
H2-Q4	NM_001143689	histocompatibility 2, Q region locus 4	0.246	0.525274
H2-Q7	NM_001198560	histocompatibility 2, Q region locus 7	0.558	0.841757
H2-Q9	NM_001201460	histocompatibility 2, Q region locus 9	0.110	0.760408
H2-T10	NM_010395	histocompatibility 2, T region locus 10	-0.552	0.129371
H2-T22	NM_001347382	histocompatibility 2, T region locus 22	0.378	0.952889
H2-T23	NM_010398	histocompatibility 2, T region locus 23	0.152	0.632918
H2-T24	NM_008207	histocompatibility 2, T region locus 24	0.308	-0.136091
Hgs	NM_001159328	HGF-regulated tyrosine kinase substrate	-0.138	-0.186883
Itga2	NM_008396	integrin alpha 2	1.526	-1.691103
Itga5	NM_001314041	integrin alpha 5 (fibronectin receptor alpha)	-0.132	0.046936
Itgam	NM_001082960	integrin alpha M	0.293	0.021581

Itgav	NM_008402	integrin alpha V	-0.075	0.219202
Itgb1	NM_010578	integrin beta 1 (fibronectin receptor beta)	0.116	0.310404
Itgb2	NM_008404	integrin beta 2	0.133	0.249882
Itgb3	NM_016780	integrin beta 3	0.718	-0.386104
Itgb5	NM_001145884	integrin beta 5	0.394	-1.082554
Lamp1	NM_001317353	lysosomal-associated membrane protein 1	-0.021	0.108735
Lamp2	NM_001017959	lysosomal-associated membrane protein 2	0.089	0.194078
M6pr	NM_010749	mannose-6-phosphate receptor, cation dependent	0.081	0.141530
Marco	NM_010766	macrophage receptor with collagenous structure	-0.732	0.073749
Mbl2	NM_010776	mannose-binding lectin (protein C) 2	0.657	-1.182090
Mrc1	NM_008625	mannose receptor, C type 1	0.713	-0.666169
Msr1	NM_001113326	macrophage scavenger receptor 1	0.015	0.568547
Ncf1	NM_001286037	neutrophil cytosolic factor 1	-0.110	0.032240
Ncf2	NM_010877	neutrophil cytosolic factor 2	0.099	0.328914
Ncf4	NM_008677	neutrophil cytosolic factor 4	-0.140	0.274824
Olr1	NM_001301094	oxidized low density lipoprotein (lectin-like) receptor 1	0.417	0.545831
Pik3c3	NM_181414	phosphatidylinositol 3-kinase catalytic subunit type 3	-0.197	-0.134110
Pikfyve	NM_001310624	phosphoinositide kinase, FYVE type zinc finger containing	-0.273	0.128686
Pla2r1	NM_008867	phospholipase A2 receptor 1	1.072	0.817910
Rab5a	NM_025887	RAB5A, member RAS oncogene family	-0.147	-0.058971
Rab5b	NM_011229	RAB5B, member RAS oncogene family	-0.066	0.078798
Rab5c	NM_001305003	RAB5C, member RAS oncogene family	-0.036	0.080130
Rab7	NM_001293652	RAB7, member RAS oncogene family	0.021	-0.270604
Rab7b	NM_001311096	RAB7B, member RAS oncogene family	-0.275	0.197554
Rac1	NM_001347530	RAS-related C3 botulinum substrate 1	-0.066	0.083768
Rilp	NM_001029938	Rab interacting lysosomal protein	0.072	-0.120689
Scarb1	NM_001205082	scavenger receptor class B, member 1	-0.650	0.790016
Sec22b	NM_011342	SEC22 homolog B, vesicle trafficking protein	-0.008	0.472352
Sec61a1	NM_016906	Sec61 alpha 1 subunit (S. cerevisiae)	0.029	0.425560
Sec61a2	NM_001356411	Sec61, alpha subunit 2 (S. cerevisiae)	-0.452	-0.524482
Sec61b	NM_024171	Sec61 beta subunit	0.121	1.035008
Sec61g	NM_001109971	SEC61, gamma subunit	0.245	0.542946
Stx12	NM_133887	syntaxin 12	0.072	0.291207
Stx18	NM_001289535	syntaxin 18	0.304	0.104214
Stx7	NM_016797	syntaxin 7	0.174	-0.241796
Tap1	NM_001161730	transporter 1, ATP-binding cassette, sub-family B (MDR/TAP)	0.220	0.134145
Tap2	NM_011530	transporter 2, ATP-binding cassette, sub-family B (MDR/TAP)	0.056	0.591344
Tcirg1	NM_001136091	T cell, immune regulator 1, ATPase, H+ transporting, lysosomal V0 protein A3	-0.458	0.370076
Tfrc	NM_001357298	transferrin receptor	-0.506	0.087999
Thbs1	NM_001313914	thrombospondin 1	4.220	-2.229074
Thbs3	NM_013691	thrombospondin 3	0.481	-0.221618
Tlr2	NM_011905	toll-like receptor 2	-0.420	0.337399
Tlr4	NM_021297	toll-like receptor 4	-0.022	0.413188
Tlr6	NM_011604	toll-like receptor 6	0.212	-0.163474
Tuba1a	NM_011653	tubulin, alpha 1A	-0.403	0.489584
Tuba1b	NM_011654	tubulin, alpha 1B	-0.399	0.610642
Tuba1c	NM_009448	tubulin, alpha 1C	-0.799	1.418716
Tuba4a	NM_001313723	tubulin, alpha 4A	-0.892	0.282360
Tuba8	NM_017379	tubulin, alpha 8	0.857	-0.597127
Tubb2a	NM_009450	tubulin, beta 2A class IIA	-0.492	0.827309
Tubb2b	NM_023716	tubulin, beta 2B class IIB	-0.013	0.501437
Tubb3	NM_023279	tubulin, beta 3 class III	0.979	0.895913
Tubb4a	NM_009451	tubulin, beta 4A class IVA	-0.080	-0.264552
Tubb4b	NM_146116	tubulin, beta 4B class IVB	-0.801	0.843197
Tubb5	NM_011655	tubulin, beta 5 class I	-0.449	0.418266
Tubb6	NM_026473	tubulin, beta 6 class V	-0.799	0.643286
Vamp3	NM_009498	vesicle-associated membrane protein 3	-0.109	0.485591

Table S4. Genes with more than 2-fold difference in expression between ZT2 and ZT14 in macrophages of control mice and more than 2-fold increase in expression by MCS in ZT14. Those genes were used Enrichment analysis of transcriptional factor using ChIP-Atlas in Figure S8A.

Gene Symbol	Genbank accession	Description	Control log2(ZT2/ZT14)	ZT14 log2(MCS/Control)
1110065P20Rik	NM_001142727	RIKEN cDNA 1110065P20 gene	-1.439	1.919500
1700007L15Rik	NR_045709	RIKEN cDNA 1700007L15 gene	-2.141	2.174114
1700071M16Rik	NR_045444	RIKEN cDNA 1700071M16 gene	-1.983	1.162904
2610306M01Rik	NR_028298	RIKEN cDNA 2610306M01 gene	-1.056	1.294584
Acpp	NM_019807	acid phosphatase, prostate	-1.357	1.183248
Ankrd66	NM_001254953	ankyrin repeat domain 66	-1.857	1.381365
Apoh	NM_013475	apolipoprotein H	-1.313	1.016634
Ar	NM_013476	androgen receptor	-1.324	1.517609
B430212C06Rik	NR_033214	RIKEN cDNA B430212C06 gene	-1.189	1.551948
BC002163	NR_002445	NADH dehydrogenase Fe-S protein 5 pseudogene	-4.239	3.349782
Bub1b	NM_009773	BUB1B, mitotic checkpoint serine/threonine kinase	-1.853	1.544959
Ccdc80	NM_026439	coiled-coil domain containing 80	-1.159	1.709616
Ccnb2	NM_007630	cyclin B2	-1.110	1.138619
Ccpg1os	NM_001198789	cell cycle progression 1, opposite strand	-1.171	1.294584
Ccr3	NM_009914	chemokine (C-C motif) receptor 3	-1.999	4.668483
Cd3d	NM_013487	CD3 antigen, delta polypeptide	-1.127	1.014183
Cdca5	NM_026410	cell division cycle associated 5	-2.206	2.111509
Cdk1	NM_007659	cyclin-dependent kinase 1	-1.509	1.235469
Cenpe	NM_173762	centromere protein E	-1.069	1.068766
Chaf1a	NM_013733	chromatin assembly factor 1, subunit A (p150)	-1.172	1.529838
Chaf1b	NM_028083	chromatin assembly factor 1, subunit B (p60)	-1.380	1.467763
Chsy3	NM_001081328	chondroitin sulfate synthase 3	-1.059	1.244893
Clu	NM_013492	clusterin	-1.063	1.025297
Col1a1	NM_007742	collagen, type I, alpha 1	-2.284	1.568521
Col1a2	NM_007743	collagen, type I, alpha 2	-2.894	2.353233
Col3a1	NM_009930	collagen, type III, alpha 1	-2.678	2.615573
Cryab	NM_001289782	crystallin, alpha B	-1.366	1.454229
Ctsw	NM_009985	cathepsin W	-1.565	1.111242
Depdc1b	NM_178683	DEP domain containing 1B	-1.139	1.175392
Dtl	NM_001305233	denticleless E3 ubiquitin protein ligase	-1.068	1.498953
Dynlt1f	NM_001166627	dynein light chain Tctex-type 1F	-2.635	2.592245
Elovl6	NM_130450	ELOVL family member 6, elongation of long chain fatty acids (yeast)	-1.179	1.059020
Erbb2	NM_001003817	erb-b2 receptor tyrosine kinase 2	-1.283	1.038732
F630042J09Rik	NR_033540	RIKEN cDNA F630042J09 gene	-1.313	1.360956
Fabp7	NM_021272	fatty acid binding protein 7, brain	-1.425	1.415953
Gadd45g	NM_011817	growth arrest and DNA-damage-inducible 45 gamma	-1.199	1.089060
Gdpd5	NM_201352	glycerophosphodiester phosphodiesterase domain containing 5	-1.313	1.459610
Gins2	NM_178856	GINS complex subunit 2 (Psf2 homolog)	-1.450	1.526347
Gm13986	NR_126479	predicted gene 13986	-2.494	1.865411
Gm14434	NM_001101804	predicted gene 14434	-10.481	7.541956
Gm17757	NR_040453	GTPase, very large interferon inducible 1 pseudogene	-2.603	2.239031
Gm6644	NR_028277	Akr1b3 pseudogene	-11.505	12.239549
Gpc6	NM_001079844	glypican 6	-1.700	1.636828
Gstm4	NM_001160411	glutathione S-transferase, mu 4	-1.033	1.054595
Hba-a1	NM_008218	hemoglobin alpha, adult chain 1	-2.803	6.398126
Hgf	NM_001289458	hepatocyte growth factor	-2.136	1.566606
Hist1h1b	NM_020034	histone cluster 1, H1b	-1.479	1.055702
Hist1h1d	NM_145713	histone cluster 1, H1d	-1.031	1.048995
Hist1h2bh	NM_178197	histone cluster 1, H2bh	-1.746	1.842105
Hist1h3b	NM_178203	histone cluster 1, H3b	-1.826	1.000186
Hist1h4j	NM_178210	histone cluster 1, H4j	-1.450	1.636828
Inpp5j	NM_172439	inositol polyphosphate 5-phosphatase J	-4.299	6.566967
Kcnj13	NM_001110227	potassium inwardly-rectifying channel, subfamily J, member 13	-1.589	1.800441
Klf11	NM_178357	Kruppel-like factor 11	-2.020	1.446732
Klrd1	NM_010654	killer cell lectin-like receptor, subfamily D, member 1	-2.038	2.034049
Lag3	NM_008479	lymphocyte-activation gene 3	-1.375	1.202246
Liph	NM_001083894	lipase, member H	-1.502	1.773074
Mamdc4	NM_001081199	MAM domain containing 4	-1.534	2.008217
Melk	NM_010790	maternal embryonic leucine zipper kinase	-1.171	1.463222
Mup19	NM_001135127	major urinary protein 19	-6.856	8.042178
Mylk	NM_139300	myosin, light polypeptide kinase	-2.607	4.090987
Ncapg	NM_019438	non-SMC condensin I complex, subunit G	-1.383	1.607443
Olfml2b	NM_177068	olfactomedin-like 2B	-2.582	2.731481
Orc1	NM_001014425	origin recognition complex, subunit 1	-1.056	1.103592
Pbk	NM_023209	PDZ binding kinase	-1.375	1.103592
Pcdh1	NM_029357	protocadherin 1	-1.741	1.674420
Pdk4	NM_013743	pyruvate dehydrogenase kinase, isoenzyme 4	-9.653	9.394320
Pira1	NM_011087	paired-Ig-like receptor A1	-1.170	1.454939
Pkhd1l1	NM_138674	polycystic kidney and hepatic disease 1-like 1	-1.632	1.360956
Plin2	NM_007408	perilipin 2	-1.530	1.572348
Proser2	NM_001159657	proline and serine rich 2	-2.734	1.674420
Rad51	NM_011234	RAD51 recombinase	-1.517	1.406699
Rcor2	NM_001320554	REST corepressor 2	-1.053	1.140765
Rgcc	NM_025427	regulator of cell cycle	-1.632	1.638729
Rmi2	NM_001033278	RecQ mediated genome instability 2	-1.692	1.150124
Rsad2	NM_021384	radical S-adenosyl methionine domain containing 2	-1.366	1.454229
Sccpdh	NM_178653	saccharopine dehydrogenase (putative)	-1.001	1.133325
Sdsl	NM_133902	serine dehydratase-like	-1.059	1.322353
Setd4	NM_145482	SET domain containing 4	-1.439	1.421807
Slc22a4	NM_001330304	solute carrier family 22 (organic cation transporter), member 4	-1.632	1.459610
Sparc	NM_001290817	secreted acidic cysteine rich glycoprotein	-2.889	1.459610
Spc25	NM_001199123	SPC25, NDC80 kinetochore complex component, homolog (S. cerevisiae)	-1.139	1.108680
Stil	NM_001304551	Scl/Tal1 interrupting locus	-1.636	1.463222

Stmn1	NM_019641	stathmin 1	-1.694	1.646594
Tcf23	NM_053085	transcription factor 23	-1.857	1.750854
Tdrkh	NM_028307	tudor and KH domain containing protein	-1.012	1.123296
Thbs1	NM_001313914	thrombospondin 1	-4.220	1.437821
Themis	NM_001305663	thymocyte selection associated	-1.024	1.125547
Tnfsf14	NM_019418	tumor necrosis factor (ligand) superfamily, member 14	-2.061	1.760902
Top2a	NM_011623	topoisomerase (DNA) II alpha	-1.218	1.310762
Tpx2	NM_001141975	TPX2, microtubule-associated	-1.742	1.381880
Trim72	NM_001079932	tripartite motif-containing 72	-1.961	1.475401
Ube2c	NM_026785	ubiquitin-conjugating enzyme E2C	-1.270	1.275804
Zbtb16	NM_001033324	zinc finger and BTB domain containing 16	-2.258	1.364748
Zdhhc2	NM_001357249	zinc finger, DHHC domain containing 2	-1.004	1.176196
Zfr2	NM_001034895	zinc finger RNA binding protein 2	-1.450	1.206276

Table S5. Primer sets for PCR analysis of gene expression

Gene		Primers
**Mouse**	***Per1***	
	Forward	5'-CCAGATTGGTGGAGGTTACTGAGT-3'
	Reverse	5'-GCGAGAGTCTTCTTGGAGCAGTAG-3'
**Mouse**	***Per2***	
	Forward	5'-GACTGCGACGACAATGGGAA-3'
	Reverse	5'-TTTGGCAGACTGCTCACTACT-3'
**Mouse**	***Cry1***	
	Forward	5'-AAGTCATCGTGCGCATTTCA3'
	Reverse	5'-TCATCATGGTCATCAGACAGA-3'
**Mouse**	***Cry2***	
	Forward	5'-CACTGGTTCCGCAAAGGACTA-3'
	Reverse	5'-CCACGGGTCGAGGATGTAGA-3'
**Mouse**	***Clock***	
	Forward	5'-TTGCTCCACGGGAATCCTT-3'
	Reverse	5'-GGAGGGAAAGTGCTCTGTTGTAG-3'
**Mouse**	***Arntl***	
	Forward	5'-GGACTTCGCCTCTACCTGTTCA-3'
	Reverse	5'-AACCATGTGCGAGTGCAGGCGC-3'
**Mouse**	***Calr***	
	Forward	5'-AAGATGCCCGATTTTACGCAC-3'
	Reverse	5'-CCCACAGTCGATATTCTGCTC-3'
**Mouse**	***Cd47***	
	Forward	5'-CACGGCCTTCAACACTGAC-3'
	Reverse	5'-ACAGGAGTATAGCCAAAATTGGG-3'
**Mouse**	***Klf4***	
	Forward	5'-GGCGAGTCTGACATGGCTG-3'
	Reverse	5'-GCTGGACGCAGTGTCTTCTC-3'
**Mouse**	***Tgf β***	
	Forward	5'-CTTCAATACGTCAGACATTCGGG-3'
	Reverse	5'-GTAACGCCAGGAATTGTTGCTA-3'
**Mouse**	***Tnfa***	
	Forward	5'-CTGAACTTGGGGGTGATCGG-3'
	Reverse	5'-GGCTTGTCACTCGAATTTTGAG-3'
**Mouse**	***Mki67***	
	Forward	5'-GCGATCCCGCAAGGTCCCTG-3'
	Reverse	5'-GCTGCCTCCTGCTGCCAGTT-3'
**Mouse**	***Gapdh***	
	Forward	5'-AAGAGGGATGCTGCCCTTAC-3'
	Reverse	5'-CGGGACGAGGAAACACTCTC-3'
**Mouse**	***E2f1***	
	Forward	5'-TGCAGAAACGGCGCATCTAT-3'
	Reverse	5'-CCGCTTACCAATCCCCACC-3'
**Mouse**	***E2f3***	
	Forward	5'-CACTACGGAGTCCCGATAGTC-3'
	Reverse	5'-GCTGCCTTGTTCAGATCCAGG-3'
**Mouse**	***E2f4***	
	Forward	5'-CTCACCACCAAGTTCGTGTC-3'
	Reverse	5'-TCTCGATCAGACCGATGCCTT-3'
**Mouse**	***Gata1***	
	Forward	5'-TGTCCTCACCATCAGATTCCA-3'
	Reverse	5'-TCCCTCCATACTGTTGAGCAG-3'
**Mouse**	***Gfi1***	
	Forward	5'-AGAAGGCGCACAGCTATCAC-3'
	Reverse	5'-GGCTCCATTTTCGACTCGC-3'
**Mouse**	***Klf5***	
	Forward	5'-GGCTCTCCCCGAGTTCACTA-3'
	Reverse	5'-ATTACTGCCGTCTGGTTTGTC-3'
**Mouse**	***Mef2c***	
	Forward	5'-ACGAGGATAATGGATGAGCGT-3'
	Reverse	5'-ATCAGTGCAATCTCACAGTCG-3'
**Mouse**	***Neurod2***	
	Forward	5'-AAGCCAGTGTCTCTTCGTGG-3'
	Reverse	5'-GCCTTGGTCATCTTGCGTTT-3'
**Mouse**	***Nfyc***	
	Forward	5'-GGATAACAAGCGTCGTACTCTTC-3'
	Reverse	5'-GTGGTCGTAGAACTGGTGGT-3'
**Mouse**	***Pax3***	
	Forward	5'-CATCCGACCTGGTGCCATC-3'
	Reverse	5'-ATTTCCCAGCTAAACATGCCC-3'
**Mouse**	***Rorc***	
	Forward	5'-CGCGGAGCAGACACACTTA-3'
	Reverse	5'-CCCTGGACCTCTGTTTTGGC-3'
**Mouse**	***Srf***	
	Forward	5'-CCAGGTGTCGGAATCTGACAG-3'
	Reverse	5'-GCTGACTTGCATGGTGGTAGA-3'
**Mouse**	***Gba***	
	Forward	5'-GCCAGGCTCATCGGATTCTTC-3'
	Reverse	5'-GAGTGCTCTCGTAACGGCT-3'
**Mouse**	***Rab3d***	
	Forward	5'-GTCAAGACGGTCTACCGACAT-3'
	Reverse	5'-CATAGCTCCGCGATAGTAGGC-3'
